# *Poria cum Radix Pini* Rescues Barium Chloride-Induced Arrhythmia by Regulating the cGMP-PKG Signalling Pathway Involving ADORA1 in Zebrafish

**DOI:** 10.3389/fphar.2021.688746

**Published:** 2021-07-30

**Authors:** Ning-Juan Yang, Yan-Ru Liu, Zhi-Shu Tang, Jin-Ao Duan, Ya-Feng Yan, Zhong-Xing Song, Ming-Geng Wang, Yu-Ru Zhang, Bai-Jin Chang, Meng-Li Zhao, Yan-Ting Zhao

**Affiliations:** ^1^ Shaanxi Province Key Laboratory of New Drugs and Chinese Medicine Foundation Research, Shaanxi Collaborative Innovation Center Medicinal Resources Industrialization, Shaanxi University of Chinese Medicine, Xianyang, China; ^2^ Key Laboratory for High Technology Research of TCM Formulae and Jiangsu Collaborative Innovation Center of Chinese Medicinal Resources Industrialization, Nanjing University of Chinese Medicine, Nanjing, China; ^3^ Shandong Buchang Pharmaceutical Co. Ltd, Xi'an, China; ^4^ Changchun University of Chinese Medicine, Changchun, China

**Keywords:** arrhythmia, *Poria cum Radix Pini*, ADORA1, cGMP-PKG signalling pathway, zebrafish

## Abstract

The traditional Chinese medicine *Poria cum Radix Pini* (PRP) is a fungal medicinal material that has been proven to play an important role in the treatment of arrhythmia. However, the mechanism of its effect on arrhythmia is still unclear. In this study, network pharmacology and metabolomics correlation analysis methods were used to determine the key targets, metabolites and potential pathways involved in the effects of PRP on arrhythmia. The results showed that PRP can significantly improve cardiac congestion, shorten the SV-BA interval and reduce the apoptosis of myocardial cells induced by barium chloride in zebrafish. By upregulating the expression of the ADORA1 protein and the levels of adenosine and cGMP metabolites in the cGMP-PKG signalling pathway, PRP can participate in ameliorating arrhythmia. Therefore, we believe that PRP shows great potential for the treatment of arrhythmia.

## Introduction

Arrhythmia is one of the most serious diseases of the cardiovascular system. Its negative effects include not only aggravating original heart disease and affecting the quality of life of patients but also inducing sudden cardiac death, which seriously threatens the life of patients (Krittayaphong et al., 2016). Approximately 600,000 people die from sudden cardiac death in China every year and approximately 390,000 people die from malignant arrhythmia every year in the United States (Wang et al., 2018; Kusumoto et al., 2018). The pathogenesis of arrhythmia is complicated. Molecular biology studies have revealed cardiac autonomic nerve dysfunction, such as excitement of the vagus nerve and inhibition of sympathetic nerves (de Araújo et al., 2018; Janssens and Michels, 2019). Abnormal structure and function of ion channels can cause arrhythmia (Torrente et al., 2020). The clinical treatment of arrhythmia is based mainly on Western medicine, such as propafenone, calcium channel blockers, and β-receptor blockers, but medical research has shown that antiarrhythmic drugs also have arrhythmic effects and that their improper use can cause more serious adverse reactions. Although the long-term use of these drugs can relieve symptoms, it results in an unsatisfactory prognosis and may even increase mortality, so there is an urgent need to explore new anti-arrhythmia treatment strategies (Brugada et al., 2020).

*Poria cum Radix Pini* (PRP) is a traditional Chinese medicine with a long history that was first referenced in the “Famous Doctors”. PRP consists of the white part of *Poria cocos (Schw.) Wolf* with pine roots in the middle (Ping, Guixin, Spleen Channel). Research shows that PRP has significant antitumour, spleen and stomach invigoration, swelling relief, mind-calming and tranquilization, immunity-enhancing and bacterium-inhibiting effects (Changhe, 2009).Pharmacological and clinical medicine research has shown that PRP can prolong sleep time and shows a synergistic effect with pentobarbital sodium. It has a good effect on most people who exhibit insomnia and difficulty falling asleep or who wake up easily (Shah et al., 2014). In addition, it can also inhibit tumour growth and exert antitumour effects by enhancing the body’s immune function. Triterpenes and polysaccharide compounds are the main active components of PRP (Xu et al., 2020; Yi et al., 2020). Studies have shown that triterpenes (poria cocoic acid) can inhibit the secretion of IL-2 and IFN-γ and affect the killing of target cells by cytotoxic T cells and the apoptosis of cardiomyocytes (Wu et al., 2014). *Poria cocos* polysaccharides can inhibit the occurrence of cardiac hypertrophy, improve haemodynamics, enhance myocardial systolic function, improve myocardial diastolic function and increase cardiac output in rats (Zhao et al., 2020b). Ergosterol compounds also exhibit good curative antitumour effects and prevent cardiovascular diseases (Kim et al., 2019), which shows that PRP presents great potential for the treatment of arrhythmia. However, the mechanism and core pathways involved in the PRP-based treatment of arrhythmia have not yet been reported, and further investigation is needed.

Databases such as the Encyclopedia of Traditional Chinese Medicine (ETCM) and the Bioinformatics Analysis Tool for Molecular Mechanisms of Traditional Chinese Medicine (BATMAN-TCM) are commonly used for network pharmacological analysis (Gong et al., 2019; Xu et al., 2019). This study is based on the use of these databases to predict the potential targets and biological pathways of PRP-based treatment of arrhythmia. Ultra-performance liquid chromatography/quadrupole time-of-flight mass spectrometry (UPLC/Q-TOF-MS) positive and negative detection modes were used to analyse alcohol extracts of PRP, and the main active ingredients were determined (Zhang et al., 2020). In addition, since zebrafish heart have a very similar electrophysiological behaviour to the human heart, thus, it has become a very interesting model for human heart system pathologies researches, like cardiomyopathy, arrhythmia, heart failure, structural and congenital heart disease etc (Gut et al., 2017; Cassar et al., 2020; Echeazarra et al., 2020). Specially, the advantage of zebrafish embryos is transparency which offer a possibility to observe heart physiology and pathology characteristics directly (Matrone et al., 2015; Crowcombe et al., 2016). Therefore, in our experiments, we prefer to use the BaCl2-induced arrhythmia zebrafish model to explore PRP treatment functions (Khisatmutdinova et al., 2006; Huang et al., 2013). A biological arrhythmia model in zebrafish was established with barium chloride (Liu et al., 2014). Then, through the correlation analysis of network pharmacology and metabolomics, the main pathway involved in the PRP-based treatment of arrhythmia was identified, and the mechanism of action of the PRP-based treatment of arrhythmia was further clarified through the quantitative analysis of key targets and metabolites in the pathway by reverse transcription polymerase chain reaction (RT-PCR) and metabolomics (Shi et al., 2020).

## Materials and Methods

### Chemicals and Reagents

Verapamil hydrochloride was purchased from Shanghai Hefeng Pharmaceutical Co., Ltd. (Shanghai, China). Barium chloride was obtained from Chongqing Maoye Chemical Reagent Co., Ltd. Optimal LC grade acetonitrile was obtained from Merck (Merck, Darmstadt, Germany). Isopropanol, formic acid and nucleotides for metabolite analyses were purchased from Sigma-Aldrich (Spruce St., St Louis, MO, United States).

### Preparation of *Poria cum Radix Pini* Extracts

PRP was obtained from Shaanxi Xingshengde Co., Ltd. (Shannxi, China) and identified by Prof. Ji-Qing Bai. The specimens were deposited at Shaanxi University of Chinese Medicine (Shaanxi, China). PRP was prepared via the following steps: precise weighing of 0.8 g PRP in a 50 ml conical bottle, dilution with 25 ml methanol, weighing, soaking for 30 min, ultrasonic processing for 60 min, cooling, and weighing again. Next, the solution was brought up to the desired weight with methanol and filtered. A 15 ml aliquot was then absorbed by continuous filtration, evaporated to approximately 1 ml in an evaporation dish in a water bath, diluted with methanol in a 5 ml capacity bottle, shaken, evaporated in an evaporating pan in a water bath, and then dissolved in deionized water for animal experiments.

### Zebrafish Embryos and Treatment

Zebrafish embryos 72 h post fertilization (hpf) were purchased from Shanghai Fish Biotechnology Co., Ltd. and maintained in Holt buffer solution (15 mM NaCl, 0.5 mM KCl, 1 mM MgSO_4_, 1 mM CaCl_2_, 0.15 mM KH_2_PO_4_, 0.05 mM Na_2_HPO_4_, 0.7 mM NaHCO_3_, 5% methylene blue; pH 7.5) on a 14 h light/10 h dark cycle at 28 ± 1°C and pH 7.5 ± 0.5.

Zebrafish embryos were randomized into six groups and placed on 48-well cell culture plates (ten embryos per group, five embryos per well): control, model (barium chloride sterile saline solution 2.1 µg/ml, 2 ml), positive control (model + 0.45 µg/ml verapamil hydrochloride), and three treatment groups (PRP-H: model + PRP extract 162.60 µg/ml, PRP-M: model + PRP extract 121.95 µg/ml, PRP-H: model + PRP extract 97.56 µg/ml). Technical details of the establishment protocol for the barium chloride-induced arrhythmia model establishment protocol and the PRP treatment dosage design are shown in Supplementary File S1. After 24 h of incubation with sterile saline solution of barium chloride, verapamil hydrochloride, or PRP extract, the morphology of the zebrafish embryo hearts was observed. All animal handling and experimental conditions were approved by the Laboratory Animal Care and Use Committee of Shaanxi University of Chinese Medicine.

### Identification of the Chemical Components of *Poria cum Radix Pini*

The concentration of PRP extract was 9.6 mg/ml. The PRP extract solution was analysed on a Waters Acquity H-Class UltraPerformance LC (Waters, MA, USA)/tandem 5600^+^ triple-quadruple time-of-flight mass spectrometer (QTOF-MS) system (AB SCIEX, MA, United States) using a Waters BEH C_18_ column (50 mm × 2.1 mm, 1.7 µm) with a gradient mobile phase of 0.1% formic acid aqueous solution (A) and 0.1% formic acid-acetonitrile solution (B) at a flow rate of 0.3 ml/min and a 30°C column temperature. The gradient program was as follows: 0–1 min, 2% B; 1–42 min, 2–100% B; 42–44 min, 100% B; 44–48 min, 100–2% B; 48–50 min, 2% B. The full scan and MS/MS experiments were performed under positive and negative modes by using an electrospray ionization (ESI) ion source. Information-dependent data (IDA) acquisition mode was used to switch automatically between MS and MS/MS acquisition with the following parameters: ion spray voltage floating: +5500 or −4500 V, declustering potential (DP): 40 V, ion source gas 1 and ion source gas 2 both set as nitrogen: 50 psi, curtain gas: 35 psi, source temperature: 500°C. The scanning range of parent ions (TOF-MS) was 100–2000 m/z. The eight strongest peaks exceeding 100 cps were collected by MS^2^, and the scanning range was 100–2000 m/z. Data were acquired using Analyst TF software (version 1.7.1, AB SCIEX). Component identification was performed by using PeakView™ software (version 2.2, AB SCIEX) and MasterView™ software (version 1.1, AB SCIEX) coupled with the TCM library database (version 1.0, AB SCIEX).

### Network Pharmacology Prediction for *Poria cum Radix Pini* Therapy

According to the UPLC-TOF-MS/MS identification results, the screening for active components of PRP and the PRP target prediction for arrhythmia treatment were performed by network pharmacology analysis on ETCM (http://www.tcmip.cn/ETCM/index.php/Home/Index/), BATMAN-TCM (http://bionet.ncpsb.org.cn/batman-tcm/index.php) , Traditional Chinese Medicine Systems Pharmacology Database and Analysis Platform (TCMSP, http://tcmspw.com/tcmsp.php), Therapeutic Targets Database (TTD, http://db.idrblab.net/ttd/) and Genecards (https://www.genecards.org/) (Liu et al., 2016).

Common targets were identified between the screened active component-related targets and disease targets (‘arrhythmia’) by VENNY 2.1.0 (https://bioinfogp.cnb.csic.es/tools/venny/), and a relationship among these targets was generated by the STRING 11.0 database (https://string-db.org/). Then, the component-disease-target association network was constructed by Cytoscape software (version 3.7.2). Finally, the enrichment of Gene Ontology (GO) functions and pathways of selected key targets were examined by using the clusterProfiler package of R software (version 3.6.1) for RPR treatment function prediction (Yu et al., 2012).

### Cardiovascular Morphological Observation and Sinus Venosus-Bulbus Arteriosus Distance Measurement

After incubation with Holt buffer solution from 60 to 72 hpf, zebrafish embryos were randomly selected (*n* = 10 each group) for the observation of cardiac morphology. The zebrafish embryos were fixed with 3% methylcellulose (AlfaAesar, Shanghai, China), and cardiac morphology observations (pericardial oedema, abnormal circulation, thrombosis, haemorrhage, etc.) and sinus venosus-bulbus arteriosus (SV-BA) distance measurement for heart tube looping quantification were performed on an ABX51 electron microscope (Olympus, Tokyo, Japan), and images were acquired under a DD71 digital camera (Olympus, Tokyo, Japan). The pericardial congestion area and the SV-BA straight line distance were calculated with ImageJ software (version 1.8.0, NIH, USA). Each experimental treatment was repeated three times (Lin et al., 2007; Gut et al., 2017; Echeazarra et al., 2020).

### Acridine Orange Staining for Apoptosis Evaluation

Acridine orange (AO) is a nucleic acid-selective metachromatic dye: AO-stained apoptotic cells show yellow-green fragments, while normal cells show uniform green or yellow-green fluorescence (Antonio et al., 2017). To evaluate the cardiomyocyte apoptosis of zebrafish embryos after barium sodium exposure and rescue treatment, five live zebrafish embryos at 96 hpf from each group were randomly selected and washed with phosphate-buffered saline (PBS, pH 7.4) to remove exposure residues and then incubated in 2.5 mg/L AO solution at 28°C in the dark for 30 min. Next, the embryos were rinsed thoroughly in PBS twice and fixed in 3% methyl cellulose solution. Images of the dorsal side of fixed zebrafish were acquired under a BX51 push-around-type electronic fluorescence microscope (Tokyo, Japan) coupled with a DD71 digital camera under a green light source. The average fluorescence density of the particle number was calculated by Image J software (Jin et al., 2019).

### Untargeted Metabolomic Analysis

#### Sample Preparation

For significant endometabolite selection, 30 live 72 hpf zebrafish embryos from each group were randomly selected and washed with PBS (pH 7.4) to remove exposure residues, placed in 1.5 ml centrifuge tubes, mixed with 1 ml of PBS solution, and centrifuged at 12,000 r/min for 10 min. Next, the PBS solution was aspirated, 1 ml of 80% methanol solution was added, and the mixture was ground and centrifuged at 12,000 r/min at 4°C for 10 min. Finally, the supernatant was extracted, concentrated and freeze-dried until use.

#### Untargeted Metabolomics UPLC-Q/TOF-MS Analysis

The freeze-dried zebrafish embryo sample was mixed with 250 µl of acetonitrile:water (1:1) solution prior to untargeted metabolomics analysis. The prepared samples were separated on a UPLC-QTOF-MS/MS system using a Waters UPLC^®^HSS T3 C_18_ column (2.1 × 100 mm, 1.7 µm), with a gradient mobile phase of aqueous solution +2 mM ammonium acetate+0.05% aqueous formic acid (A)-acetonitrile: isopropanol = 1:1 + 2 mM ammonium acetate-0.05% aqueous formic acid (B), at a flow rate of 0.3 ml/min and a column temperature of 40°C. The gradient program was as follows: 0–1.5 min, 2% B; 1.5–23 min, 1–60% B; 23–24 min, 60–98% B; 24–27 min, 98% B; 27–27.1 min, 27.1–33 min, 1% B. Full scans and MS/MS experiments with information-dependent data (IDA) acquisition mode were performed by using an ESI ion source with the following parameters: ion spray voltage floating: +5500or −4500 V, declustering potential (DP): 40 V, ion source gas 1 and ion source gas 2 both set as nitrogen: 50 psi, curtain gas: 35 psi, source temperature: 500°C. The scanning range of parent ions was 70–1200 m/z. The twelve strongest peaks exceeding 100 cps were collected by MS^2^, and the scanning range was 70–1200 m/z. Data were acquired using Analyst TF software (version 1.7.1, AB SCIEX). Metabolites were identified by using PeakView™ software (version 2.2, AB SCIEX) and MasterView™ software (version 1.1, AB SCIEX) coupled with “the Accurate Mass Metabolite Spectral Library” (version 1.0, AB SCIEX). The combined score of confidence setting parameters were as follows: mass error 40%, retention time 0%, isotope 20%, library hit 40%, and formula finder 0%.

#### Untargeted Differential Metabolome Screening

To identify targeted endo-metabolites, metabolite database matching data were extracted and converted to Excel files (*.xls) for matrix formation. Then, the pretreatment matrix was subjected to SIMCA-P 14.0 (Umetrics, Sweden) software for partial least squares (PLS) analysis between the control and model groups to identify the differential metabolome that corresponded to a statistical *p* value of < 0.05 and a variable importance in projection (VIP) value of > 1.0 (Liu et al., 2019). To select the therapeutic-related endo-metabolites among the control, model, positive control, PRP-H, PRP-M and PRP-L groups, the differential metabolome was next distinguished by PCA on the MetaboAnalyst 4.0 online platform (http://www.metaboanalyst.ca) (Xia et al., 2009).

#### Metabolic Pathway Enrichment

Further analyses were conducted to identify and visualize the effect of the PRP extract on the metabolic pathways related to arrhythmia. The differential metabolites screened by metabolomics and the key targets predicted on the basis of network pharmacology were input into the MetaboAnalyst database, with *p* <0.05 as the card value, and the pathway library (Kyoto Encyclopedia of Genes and Genomes (KEGG)) was selected for pathway enrichment analysis.

### Targeted Metabolites Determination

To analyse the targeted metabolomic selection results, potential metabolite markers were quantitated by comparison to the areas of the peaks for the external standards (method validation is shown in Supplementary File S2, Supplementary Table S1).

### Quantitative Reverse Transcriptase Polymerase Chain Reaction Analysis

After treatment at 96 hpf, zebrafish embryos were randomly selected (*n* = 30 each group) for qRT-PCR analysis. Thirty milligrams of zebrafish embryos was homogenized in TRIzol reagent (Servicebio Technology Co., Ltd., Wuhan, China) to extract total RNA. The extracted RNA was used as a template, and cDNA was synthesized by reverse transcription with appropriate primers according to the instructions of the Servicebio® RT First Strand cDNA Synthesis Kit (G3330, Servicebio, Wuhan, China). qRT-PCR was performed in an ABI 7900HT Fast Real-Time PCR system (Bio-Rad Bole Gradient, California, America). The primer sequences were as follows (ref. NM_001128584.1, 242 bp, 60°C, Servicebio): TTC​TGA​CCC​AAA​GTT​CCA​TCC​T (forward) and CTC​AAA​CTG​GCA​GGT​GAC​GAT (reverse). Levels of the Adenosine A1 Receptor (ADORA1) mRNAs were normalized to the GAPDH mRNA level and determined using the 2^−ΔΔCt^ method, and all reactions were performed in triplicate.

### Statistical Analysis

Statistical Program for the Social Sciences (SPSS) 26.0 was used for statistical analysis. All the data are expressed as the mean ± SD. One-way analysis of variance (ANOVA) was used to compare the data among the groups. GraphPad Prism 8 software was used to generate all graphics, and *p* < 0.05 was considered statistically significant.

## Results

### UPLC/Q-TOF-MS Components Identification of *Poria cum Radix Pini*

**FIGURE 1:**
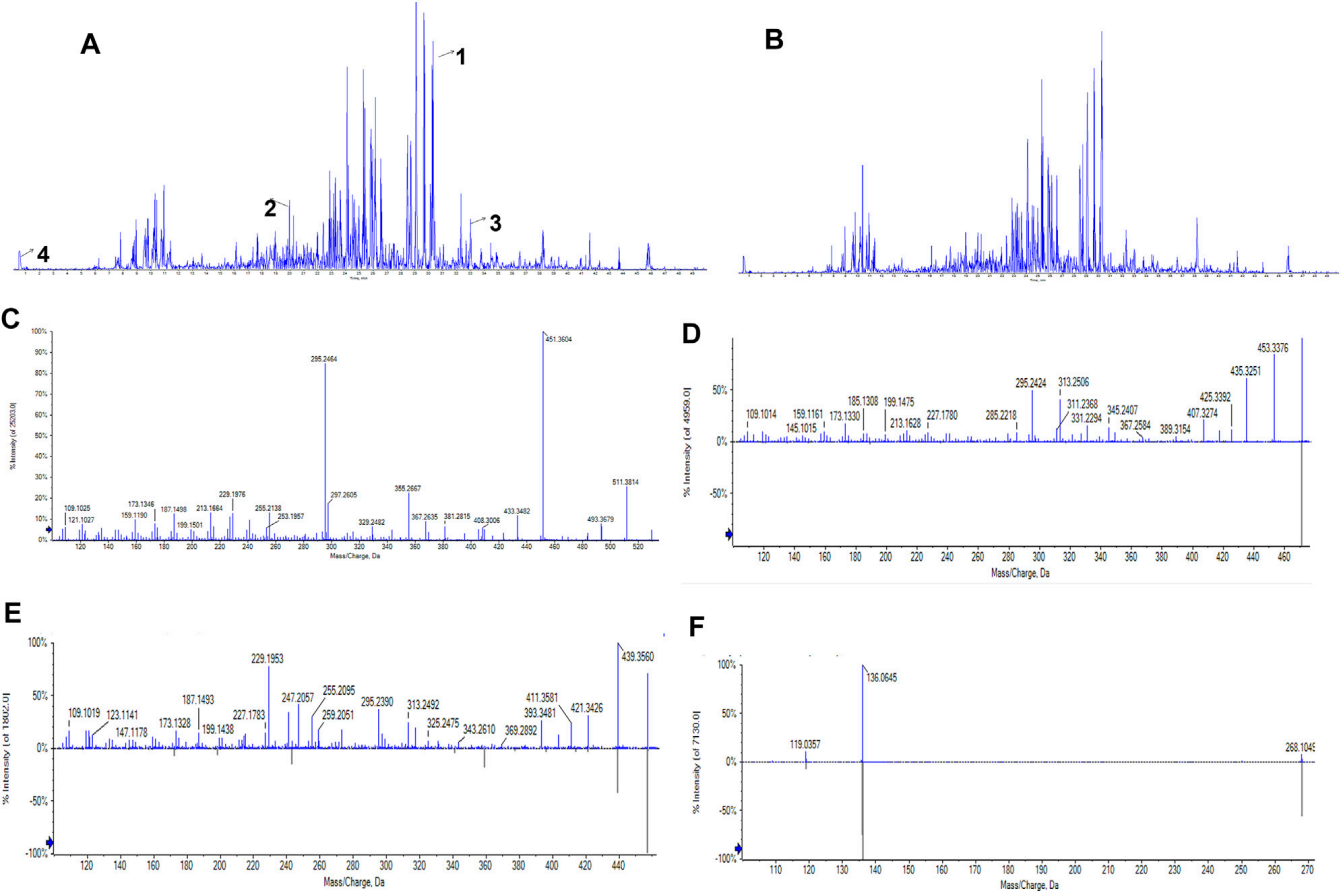
UPLC/Q-TOF-MS total ion current chromatogram of the PRP alcohol extract. **(A)** Total ion current chromatogram in positive ion mode. Numbers represent the following compounds:1. Pachymic acid, 2. Glycyrrhetinic acid, 3. Oleanolic acid, 4. Adenosine. **(B)** Total ion current chromatogram in negative mode. The retention times of the four compounds were 30.317, 19.867, 33.012, and 0.55 min, respectively. **(C)** MS/MS spectrum of pachymic acid; **(D)** MS/MS spectrum of glycyrrhetinic acid; **(E)** MS/MS spectrum of oleanolic acid; **(F)** MS/MS spectrum of adenosine.

Under the optimized chromatographic and mass spectrometry conditions, UPLC/Q-TOF-MS in positive and negative detection modes was used to analyse the PRP extracts (Figures 1A,B). The results showed that 31 compounds were identified in positive ion mode and 5 in negative ion mode (Supplementary File S3, Supplementary Table S2). According to the molecular weight, retention time, and MS/MS data, four compounds were identified: pachymic acid, glycyrrhetinic acid, oleanolic acid and adenosine (Figures 1C–F) (Supplementary File S3, Supplementary Table S3) (Qian et al., 2018).

### Network Pharmacological Analysis for *Poria cum Radix Pini* Therapy

**FIGURE 2:**
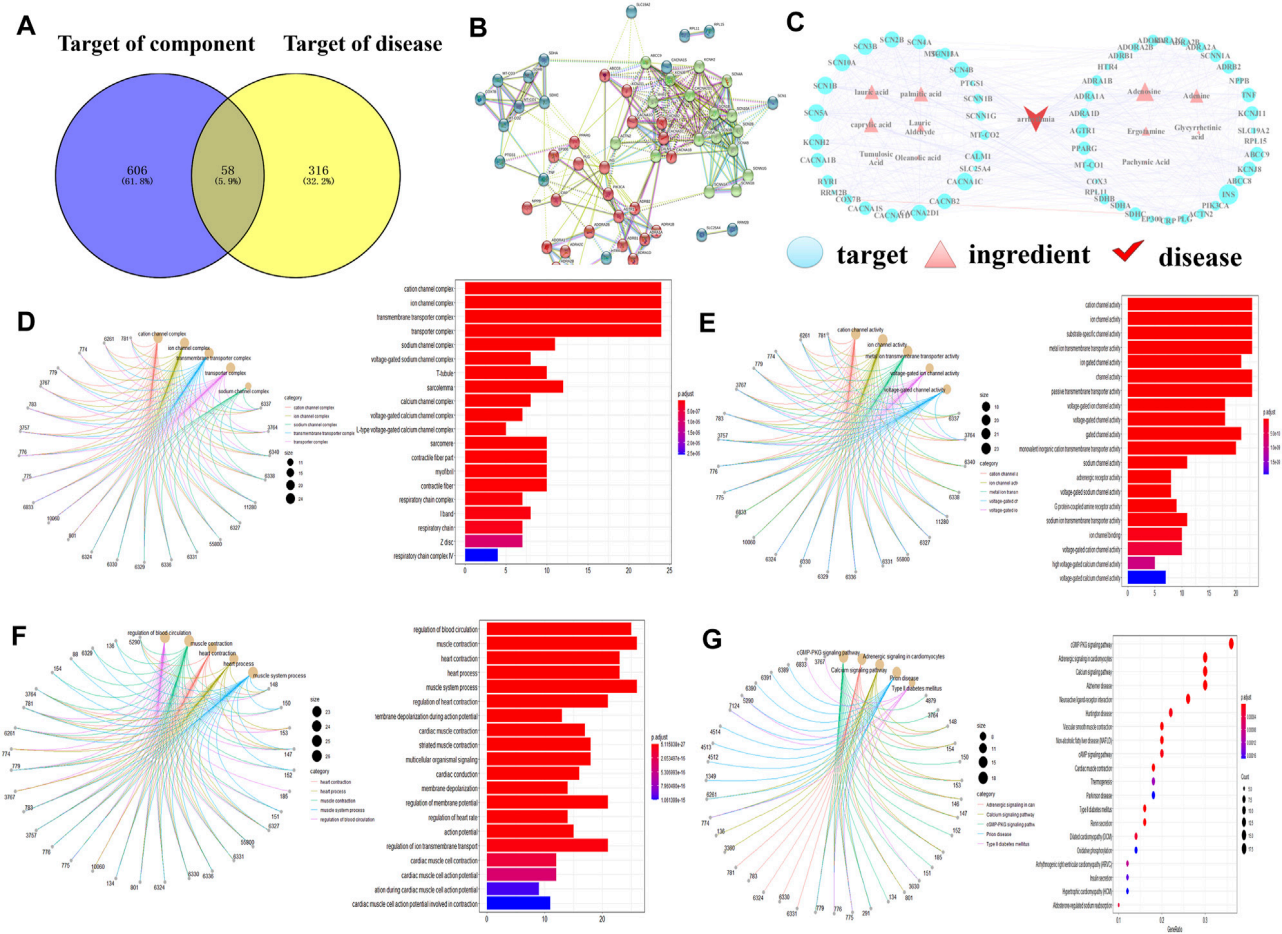
Network pharmacology prediction results. **(A)** ‘Component-disease-target’ intersection Venn diagram. **(B)** Intersection target PPI diagram. The closer the nodes were, the more likely they were to appear red. The larger the nodes, the closer the relationship was between the corresponding targets and other targets in the network. **(C)** ‘Component-disease-target’ network correlation diagram. **(D)** GO enrichment analysis - cellular component (CC). **(E)** GO enrichment analysis - molecular function (MF). **(F)** GO enrichment analysis - biological process (BP). **(G)** KEGG metabolic pathway analysis. Gene symbols were presented by PubMed Entrez ID.

By combining UPLC/Q-TOF-MS identification and database search results, 11 compounds were identified and selected (Table 1). For these compounds, 606 putative targets were predicted from the ETCM, TCMSP and Batman-TCM databases (Supplementary File S4, Supplementary Table S4). A total of 316 targets related to arrhythmia were obtained against the ETCM, TTD, and GeneCards databases (Supplementary File S4, Supplementary Table S5). In Figure 2A, we first extracted 58 intersection targets from the overlaps between the disease gene set and PRP target sets (Supplementary File S4, Supplementary Table S6). In the following study, the relationships of these 58 targets were connected as a PPI network for importance evaluation (Supplementary File S4, Supplementary Table S7). As shown in Figure 2B, the PPI k-means classification results suggested that 25 genes, iADORA1, ADORA2B, ADRA1A, ADRA1B, and ADRA1D, play a more important role in arrhythmia pathology and PRP treatment.

**TABLE 1:** Chemical identification combining component characterization and database search.

Number	Name	Molecular Formula	Mass (Da)	Source
1	Pachymic Acid	C_33_H_52_O_5_	529.38875	MS、MS2、TCMSP、ETCM
2	Oleanolic acid	C_30_H_48_O_3_	457.3676	MS、MS2
3	Glycyrrhetinic acid	C_30_H_46_O_4_	471.34688	MS、MS2
4	Adenine	C_5_H_5_N_5_	136.06177	MS、BATMAN-TCM、ETCM
5	Ergotamine	C_33_H_35_N_5_O_5_	581.26382	ETCM
6	Lauric Aldehyde	C_12_H_24_O	184.18272	TCMSP、ETCM
7	Adenosine	C_10_H_13_N_5_O_4_	268.10402	MS、MS2
8	Palmitic acid	C_16_H_32_O_2_	256.24023	TCMSP、ETCM
9	Lauric acid	C_12_H_24_O_2_	200.17763	TCMSP、ETCM
10	Caprylic acid	C_8_H_16_O_2_	144.11503	TCMSP、ETCM
11	Tumulosic acid	C_31_H_50_O_4_	486.37091	TCMSP、ETCM

To further explore PRP multicomponent and multitarget therapy functions, all 58 targets of the PPI network were mapped to “component-disease” connections, and then the “component-disease-target” network of PRP therapy was constructed by Cytoscape software (Figure 2C). This network included 70 nodes, which illustrated potential interplay among 1 disease type, 11 chemical components, and 58 targets. Since the node size is proportional to the importance of nodes involved in the pathway, 7 components involved in the therapy pathway, including adenosine, palmitic acid, caprylic acid, lauric acid, and adenine. In addition, 58 targets, such as ADORA1, ADORA2B, and CACNA1B, were considered potential therapeutic targets (Supplementary File S4, Supplementary Table S8).

GO analysis revealed that the targets were significantly enriched in 581 pathways for PRP therapy, specifically “complex ion channels and transmembrane transporters”, “ion channel activity and metal ion transmembrane transport protein activity”, and “myocardial contraction, the regulation of myocardial contractility and the regulation of blood circulation” (Figures 2D–F, Supplementary File S4, Supplementary Table S9). Similarly, KEGG pathway enrichment analysis showed that the targets were significantly enriched in 193 pathways for PRP therapy, specifically the “cGMP-PKG signalling pathway” and “calcium ion signalling pathway” (Figure 2G, Supplementary File S4, Supplementary Table S10). As the GO and KEGG analyses suggested that most adenosine receptors (ADORAs) under PRP treatment were involved in arrhythmia-related pathways, these 58 targets were used to perform metabolomic joint pathway analysis.

### *Poria cum Radix Pini* can Rescue Barium Chloride-induced Cardiac Defects in Zebrafish Embryos

**FIGURE 3:**
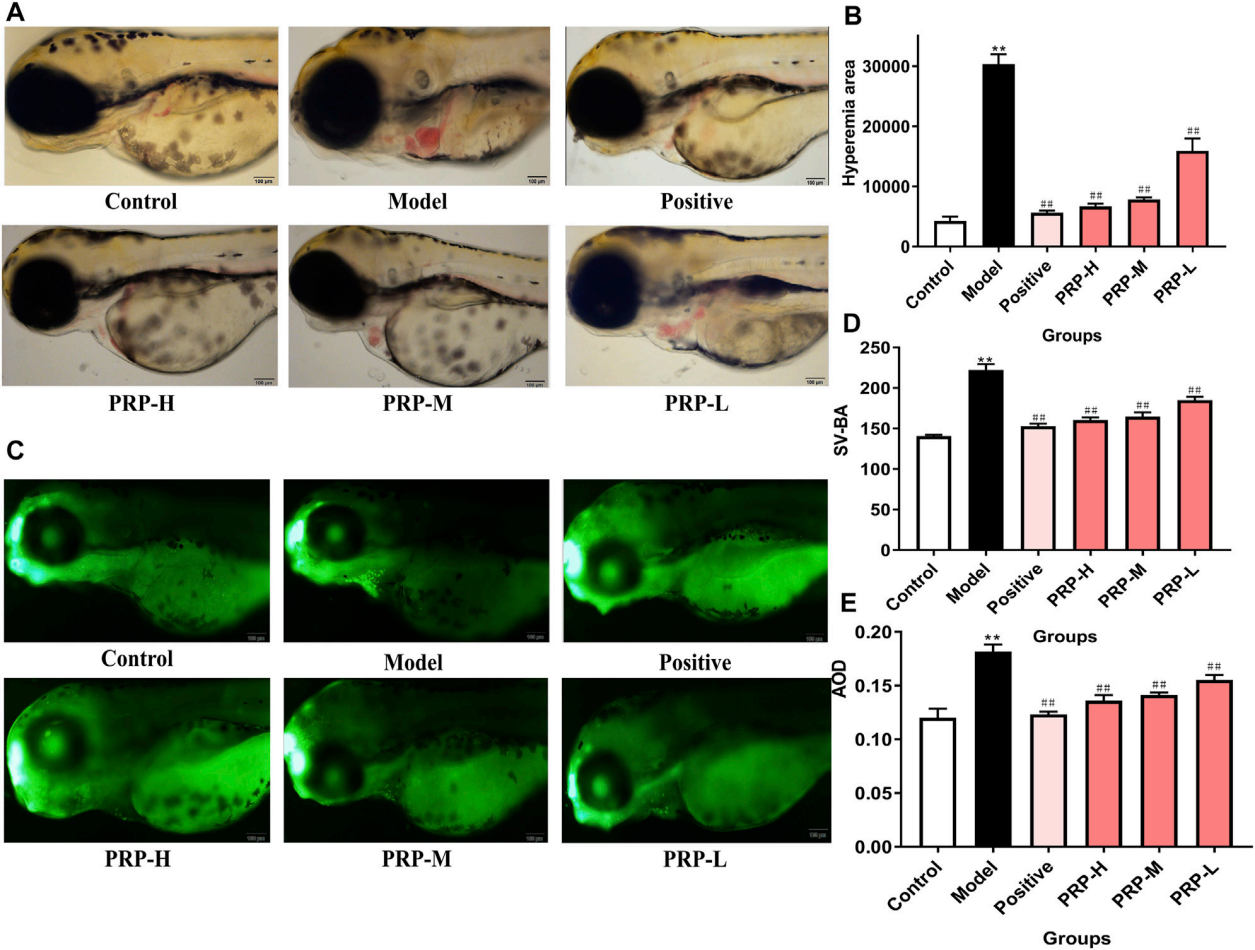
The effect of PRP on the heart development of zebrafish embryos following barium chloride treatment. **(A)** Morphological observations of the heart development of zebrafish embryos under a microscope, with the heart shown in the yellow box (X10). **(B)** The cardiac congestion area of zebrafish embryos was calculated. **(C)** The results of acridine orange staining. The apoptosis of zebrafish embryos cardiomyocytes observed under fluorescence microscopy is shown in the yellow box (X10). **(D)** The SV-BA distance was measured. **(E)** The average fluorescence density was calculated.All data are presented as means ± SD, *n* = 6. *p < 0.05, **p < 0.01, control versus model; ^#^ *p* < 0.05, ^##^ *p* < 0.01, model versus positive, PRP-H, PRP-M, and PRP-L.

In the morphological phenotype observation, PRP extracts showed a significant and dose-dependent rescue function against barium chloride-induced morphological phenotypes (Figure 3A). Calculating the cardiac congestion area in zebrafish showed that the cardiac congestion area was 605.42% larger in the zebrafish model group than in the control group. However, the cardiac congestion areas in the positive control group, PRP-H group, PRP-M group and PRP-L group showed decreases of 81.44, 78.05, 74.22 and 47.54%, respectively, compared to that in the model group. These findings further proved that PRP can lessen the degree of cardiac congestion induced by barium chloride in zebrafish (Figure 3B, Supplementary File S5, Supplementary Tables S11, S11-1, S11-2). Compared with the control group, the model group zebrafish showed a 58.10% increase in the SV-BA distance. Compared to the model group, the SV-BA distance in the verapamil exposure group led to a significant 31.30% decrease, while the PRP-H, PRP-M, and PRP-L treatment groups showed 27.83, 25.96, and 16.82% decreases, respectively (Figure 3D, Supplementary File S5, Supplementary Tables S12, S12-1, S12-2).

### *Poria cum Radix Pini* can Inhibit Cardiomyocyte Apoptosis in Zebrafish Embryos

After AO staining, compared with the control group, a large number of apoptotic cells (green debris particles) were found in zebrafish embryo hearts, and the average fluorescence density was 51.18% higher than that in the control group. At 96 hpf, the numbers of apoptotic cells in zebrafish embryo hearts were significantly and dose-dependently decreased in the PRP treatment group. Compared to the model group, the average fluorescence density of the PRP-H, PRP-M and PRP-L groups showed declines of 25.13, 22.20 and 14.50%, respectively. These results indicated that PRP could rescue barium chloride-induced apoptosis in the hearts of zebrafish embryos in a concentration-dependent manner (Figures 3C,E, Supplementary File S5, Supplementary Tables S13, S13-1, S13-2).

### *Poria cum Radix Pini* can Regulate Metabolite Disorders Induced by Barium Chloride in Zebrafish Embryos

**FIGURE 4:**
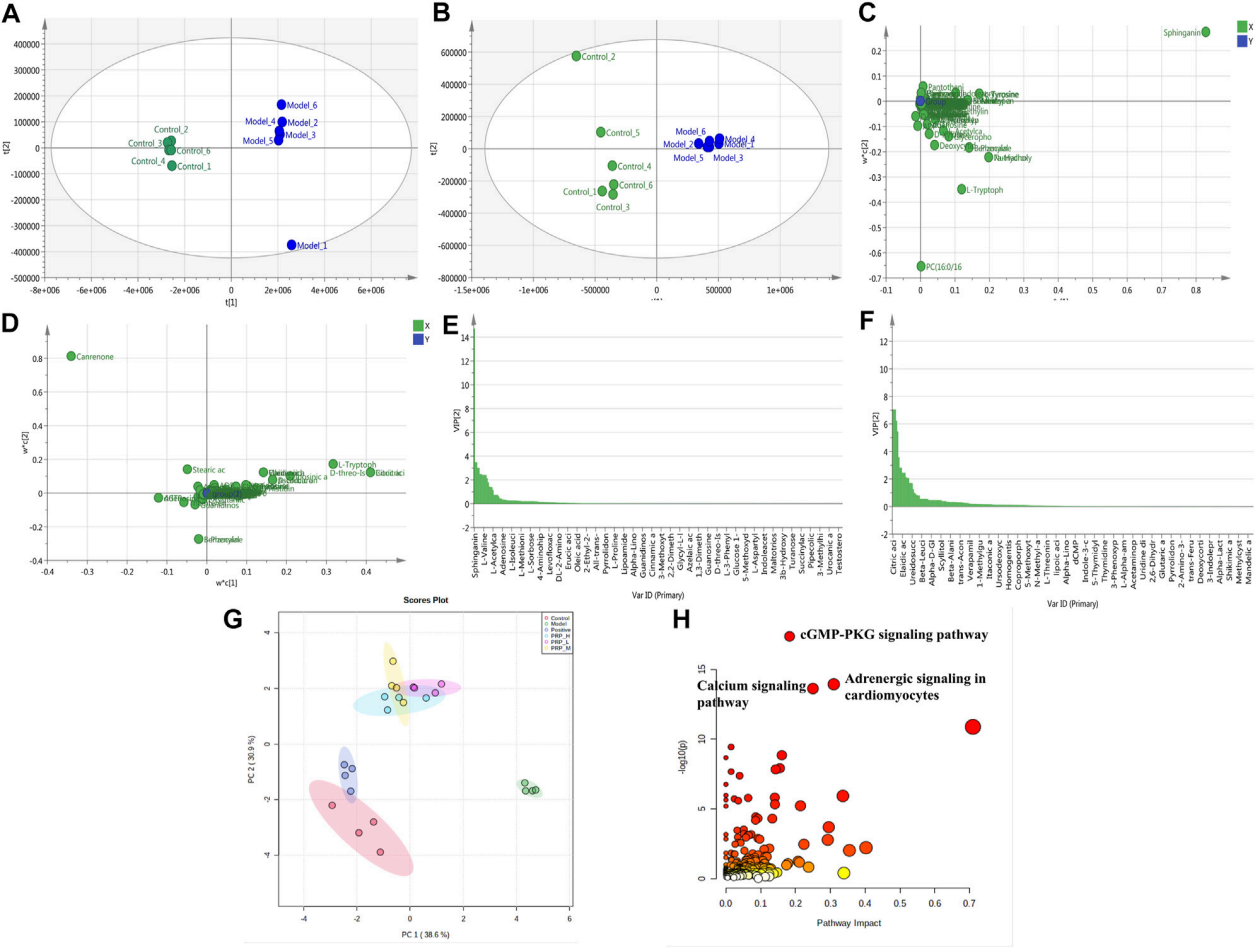
Effects of PRP on metabolite alterations induced by barium chloride in zebrafish embryos. R^2^Y = 0.999, Q^2^ = 0.995 in positive ion mode; R^2^Y = 0.986, Q^2^ = 0.984 in negative ion mode; **(A)** 2D scores plot in positive ion mode; **(B)** 2D scores plot in negative ion mode; **(C)** Loading plot in positive ion mode; **(D)** Loading plot in negative ion mode; **(E)** VIP diagram in positive ion mode; **(F)** VIP diagram in negative ion mode; **(G)** PCA scores plot of different metabolites in each group; **(H)** Metabolic pathway enrichment analysis diagram.

PLS analysis of the untargeted metabolome showed a distinct change in metabolite profiles between the model embryos and control embryos, represented by the substantial metabolomic perturbation of the cardiac system observed in these embryos (three experiments, Figures 4A,B). Then, significant features were selected based on a selected criterion with an adjusted *p*-value cut-off of <0.05. Statistical analysis of the metabolomics data from the barium chloride-treated group and the control group indicated significant differences in ions between these two groups, as shown in the loading plot (Figures 4C,D). The VIP values were also used to calculate the feature importance by the cut-off value of weight VIP > 1 (Figures 4E,F). Following the selection flow, a total of 17 potential differential metabolites were identified in positive and negative ion modes (Table 2), including amino acid components such as L-tyrosine, L-valine, L-tryptophan, indoleacrylic acid, and L-histidine and choline components such as glycerophosphocholine, L-acetylcarnitine and adenosine triphosphate.

**TABLE 2:** Potential differential metabolites induced by barium chloride in zebrafish embryos.

Number	Name	Formula	m/z	Adduct	VIP	*p* Value
1	L-Tyrosine	C_9_H_11_NO_3_	181.0739	+H	3.02665	3.2307 × 10^–14^
2	Nutriacholic acid	C_24_H_38_O_4_	390.2770	+H	3.50493	3.154 × 10^–05^
3	Indoleacrylic acid	C_11_H_9_NO_2_	187.0633	+H	1.79193	1.8004 × 10^–06^
4	L-Valine	C_5_H_11_NO_2_	117.0790	+H	2.42267	6.938 × 10^–06^
5	Benzocaine	C_9_H_11_NO_2_	165.0790	+H	2.49461	2.0293 × 10^–06^
6	Glycerophosphocholine	C_8_H_20_NO_6_P	257.1028	+H	1.45259	2.2183 × 10^–4^
7	L-Acetylcarnitine	C_9_H_17_NO_4_	203.1158	+H	1.16715	1.6222 × 10^–04^
8	Ascorbic acid	C_6_H_8_O_6_	176.0321	-H	2.83443	7.0845 × 10^–12^
9	Adenosine triphosphate	C_10_H_14_N_5_O_7_P	347.0631	-H	2.07961	6.416 × 10^–04^
10	Ureidosuccinic acid	C_5_H_8_N_2_O_5_	176.0433	-H	1.69662	3.963 × 10^–10^
11	Glutathione	C_10_H_17_N_3_O_6_S	307.0838	-H	1.26381	6.9709 × 10^–10^
12	Triamterene	C_12_H_11_N_7_	253.1076	-H	1.01154	3.853 × 10^–10^
13	Citric acid	C_10_H_8_O_7_	240.0270	-H	7.06906	2.5526 × 10^–03^
14	Inosinic acid	C_10_H_13_N_4_O_8_P	348.0471	-H	3.59027	1.9504 × 10^–12^
15	L-Histidine	C_6_H_9_N_3_O_2_	155.0695	-H	2.152	1.2383 × 10^–04^
16	L-Tryptophan	C_11_H_12_N_2_O_2_	204.0899	-H	5.46315	1.26 × 10^–14^
17	Canrenone	C_22_H_28_O_3_	340.2038	-H	6.2536	2.257 × 10^–02^

Furthermore, to clarify whether PRP administration can regulate barium chloride-induced metabolite alterations, comparisons among the control group, model group, positive control group and PRP-H, PRP-M and PRP-L groups were analysed by using the MetaboAnalyst database. As shown in Figure 4G, the PCA score plot showed obvious sample clusters among the control, model, positive control and PRP-treated groups, where the PRP-H group and PRP-M group were close to the control group but separated from the model group. The results indicated that PRP treatment could improve barium chloride-induced metabolomic profile imbalance in zebrafish embryos.

### *Poria cum Radix Pini* Rescues Metabolite Profiles From the ADORA1-Mediated cGMP-PKG Signalling Pathway

To further investigate the roles of the enriched metabolite pathways and dissect which of the screened targets contributed to these pathways, the candidate targets from the network pharmacology selection were mapped to 17 differential endometabolites by joint-pathway analysis on the MetaboAnalyst database (Figure 4H). Gene-metabolic pathway correlation analyses showed that the “cGMP-PKG signalling pathway” (*p* < 0.001) was highly enriched among the 72 enriched metabolic pathways. In this pathway, ADORA1 is a highly ranked protein that acts as an adenosine receptor to reduce cardiomyocyte calcium overload in the occurrence of arrhythmia diseases, and adenosine and cGMP are highly ranked metabolites (Szentmiklosi et al., 2015; Deb et al., 2019).

**FIGURE 5:**
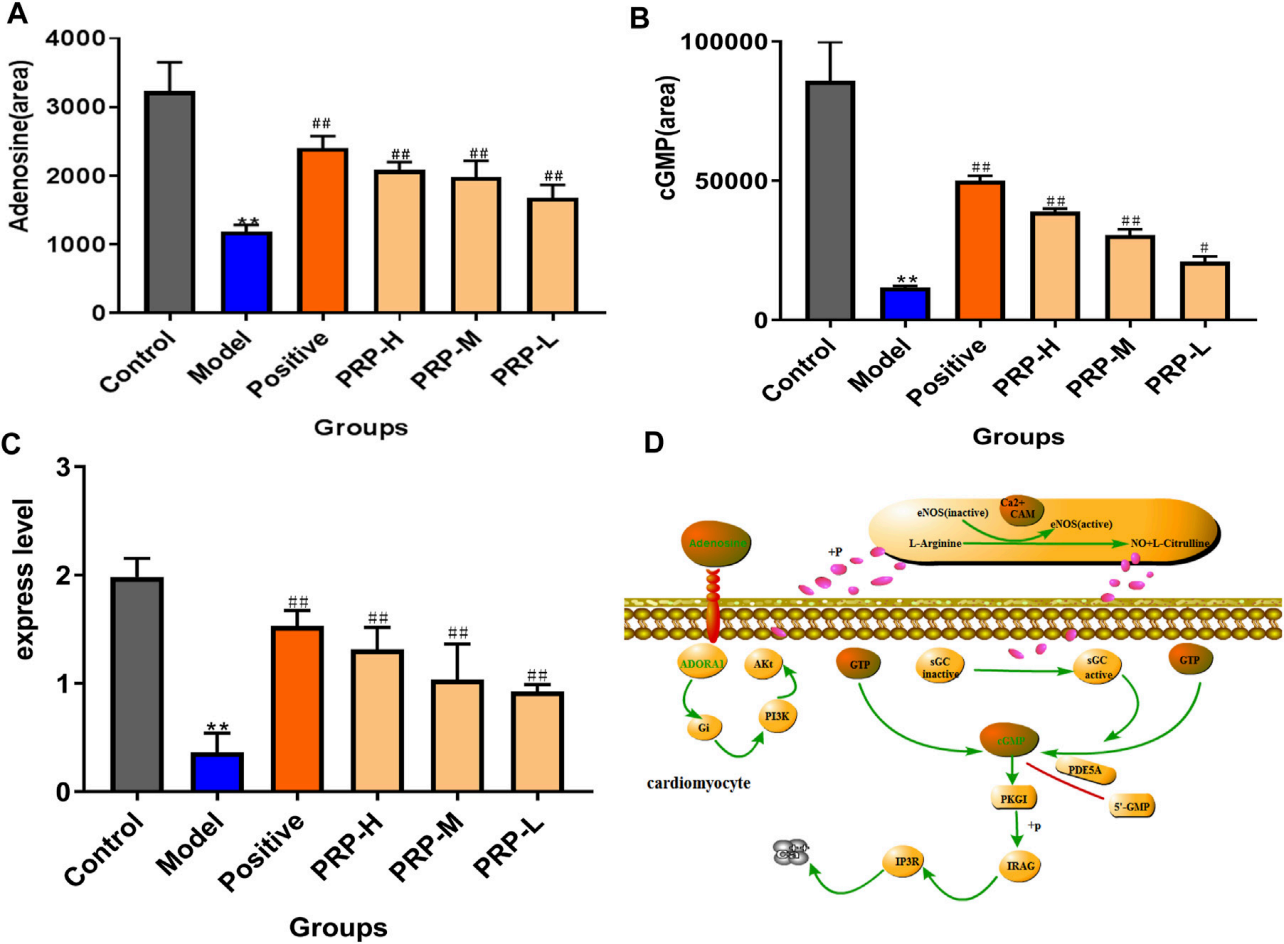
PRP-mediated regulation of the levels of adenosine and cGMP metabolites and the expression level of ADORA1 in the cGMP-PKG signalling pathway. **(A)** Quantitative evaluation of adenosine metabolism; **(B)** quantitative evaluation of cGMP metabolic levels; **(C)** expression levels of ADORA1 in zebrafish determined by reverse transcription polymerase chain reaction; **(D)** ADORA1-mediated cGMP-PKG pathway map.

Next, by quantifying the levels of adenosine and cGMP in embryo samples using LC-MS in multiple reaction monitoring (MRM) mode, barium chloride induced a significant decreasing trend in adenosine by 63.30% and cGMP by 78.86% compared with the control ones. PRP therapy significantly reversed these changes: the adenosine and cGMP levels in the PRP-H group were increased by 75.84 and 231.35%, in the PRP-M group were increased by 66.71 and 159.57%, and in the PRP-L group were increased by 41.71 and 78.86%, respectively (Figures 5A,B, Supplementary File S6, Supplementary Tables S14–S15-2). ADORA1 expression was determined using qRT-PCR assays to examine how PRP treatment influenced barium chloride-induced arrhythmia in zebrafish embryos (Figure 5C, Supplementary File S6, Supplementary Tables S16, S16-1, S16-2). Interestingly, ADORA1 expression was significantly decreased by 81.65% (*p* < 0.01) in the model group. PRP therapy restored ADORA1 expression at three dosages, with increases of 320.53, 261.96, 184.56 and 154.58%, respectively. The results showed that PRP could increase the expression level of ADORA1 in the cGMP-PKG signalling pathway. These findings indicated that barium chloride disturbed the cGMP-PKG signalling pathway, which resulted in arrhythmia. This was attributed to ADORA1 downregulation associated with adenosine and cGMP decline and PRP treatment rescued these trends towards an imbalance.

## Discussion

PRP is a traditional fungal medicinal material that is often used as the key medicine for the treatment of arrhythmia. Modern pharmacological studies have shown that PRP water decoction shows a similar central inhibitory effect to synergistic pentobarbital sodium, which further demonstrates its anti-arrhythmic effects, and it can be used together with *Poria cocos* to treat heart palpitations caused by deficiencies of the heart and spleen (Shah et al., 2014). In addition, its main active components, such as triterpenoids and polysaccharides, have been proven to play an important role in the treatment of arrhythmia (Wu et al., 2014; Li et al., 2015). Studies have indicated that cardiomyocyte apoptosis plays an important role in arrhythmia, and the severity of apoptosis is positively correlated with the arrhythmia score (Niu et al., 2020). In addition, zebrafish heart congestion, an extended SV-BA distance, pericardial oedema and other cardiac morphological changes can cause heart damage and arrhythmia. In this study, by observing the heart morphology of zebrafish with arrhythmia induced by barium chloride, it was found that PRP administration could significantly improve the cardiac congestion area, the SV-BA interval and the degree of myocardial cell apoptosis and promote the normal development of the heart in zebrafish, which further indicated the great potential of PRP for the treatment of arrhythmia. However, due to the complex pathogenesis of arrhythmia and the efficacy characteristics of multicomponent, multitarget and multipathway drugs, it is still a great challenge to explain the mechanism underlying the effects of PRP in the treatment of arrhythmia.

In the analysis of PPI and component-disease-target network pharmacology results, we found that the ADORA1 protein may be the main target in the PRP-based treatment of arrhythmia and that it participates in multiple signalling pathways, such as the cGMP-PKG, cAMP, and cardiac muscle contraction pathways. These pathways all play important roles in heart disease (Zhu et al., 2020). In addition, we verified through PCR that the expression levels of the ADORA1 protein in the PRP-treated groups were significantly higher than those in the model group. Studies have indicated that calcium overload in cardiomyocytes is one of the important causes of arrhythmias and that not only does adenosine affect the intracellular calcium ion current, but adenosine receptors can also cause arrhythmias by affecting calcium ion homeostasis (Greene et al., 2016). Among the adenosine receptors, ADORA1 shows the highest affinity for adenosine, which is mainly distributed in the sinoatrial and atrioventricular nodes and the atrial muscle of the heart and can play a role in myocardial protection by regulating oxygen free radicals, heat shock proteins, interleukins and L-type calcium channels (Goldman et al., 2010). Therefore, we highlight the finding that PRP can significantly restore the high gene expression of ADORA1 and thereby play a role in the treatment of arrhythmia.

In this study, the enrichment analysis of KEGG metabolic pathways showed that PRP can regulate a variety of signalling pathways, including the cGMP-PKG signalling pathway, the adrenergic signalling pathway in cardiomyocytes, and the calcium ion signalling pathway. Among these pathways, the cGMP-PKG signalling pathway showed the highest significance and can be regarded as one of the most critical signalling pathways in the PRP-based treatment of arrhythmia. The results of network pharmacology and metabolomics correlation analysis showed that the cGMP-PKG signalling pathway was the most significant, which was consistent with the network pharmacology prediction results, indicating that PRP may participate in the treatment of arrhythmia by regulating proteins and metabolites in the cGMP-PKG signalling pathway (Figure 5D). The adenosine receptor protein ADORA1 is activated and conjugates with the Gi protein to promote the phosphoinositide 3-kinase/protein kinase B (PI3K/Akt) signalling pathway. Studies have shown that the PI3K/Akt/eNOS signalling pathway has myocardial protective effects, such as antioxidative and antiapoptotic effects (Yao et al., 2014; Zhang and Zhang, 2021). NO is a factor that regulates heart relaxation. Its formation process involves L-arginine and nitric oxide synthase (NOS). Following the catalytic synthesis of NO and L-citrulline, calcium ions enter the cell and bind with calmodulin to form a complex to activate eNOS. The resulting NO diffuses into the intercellular space and crosses the membrane of adjacent target cells to activate SGC and convert GTP into cGMP (Silberman et al., 2010). cGMP activates the most important downstream target, PKG I. Activated PKG I acts on its downstream targets to regulate the inositol triphosphate receptor (IP3R) in cardiomyocytes and increase calcium ion regeneration in the sarcoplasmic reticulum, thereby inhibiting platelet activation, reducing cell apoptosis and exerting a negative inotropic effect on cardiomyocytes to protect the cardiovascular system (Zhang et al., 2007). Studies have also shown that the cGMP-PKG signalling pathway plays an important role in the cardioprotective mechanism of pretreatment and posttreatment. It can prevent cell necrosis-induced stress, reduce cell apoptosis and negative inotropic effects on cardiomyocytes, protect the cardiovascular system and reduce the occurrence of arrhythmias (Yu et al., 2018).

In the cGMP-PKG signalling pathway, the metabolites adenosine and cGMP play extremely important roles. In this study, the levels of adenosine and cGMP in zebrafish with arrhythmia were quantitatively determined after various treatments and were shown to be significantly higher in the PRP-treated groups than in the model group. These findings indicated that PRP could increase adenosine and cGMP metabolism, improve heart shape, and reduce the occurrence of arrhythmia in zebrafish. As an anti-arrhythmic drug, adenosine can induce independent potassium currents in the atrioventricular node, reduce the influx of calcium ions, slow atrioventricular conduction and reduce the occurrence of arrhythmia. Studies have indicated that inflammation and oxidative stress can be reduced by increasing adenosine levels and that drugs with such effects exert a protective effect against myocardial ischaemia-reperfusion injury in mice (Zhao et al., 2020a). The metabolite cGMP can activate protein kinase G to activate phosphodiesterase (PDE), cleave cAMP, inhibit the phosphorylation of muscle fibre membrane proteins, and reduce the intake of calcium ions through the cell membrane, thereby protecting the heart. Studies have shown that after administration, the release of cGMP in the isolated hearts of rats with myocardial ischaemia is increased, which inhibits ischaemia-induced cardiac sympathetic hyperactivity and cardiac electrophysiological instability and attenuates arrhythmia (He et al., 2020). Therefore, we believe that PRP can play a role in the treatment of arrhythmia by increasing the metabolic levels of adenosine and cGMP in the cGMP-PKG pathway.

The main advantage of this study was that we used the methods of network pharmacology and metabolomics correlation analysis to determine the pathways underlying the effects of PRP on arrhythmia and experimentally verified the key target ADORA1 and the main metabolites adenosine and cGMP in the pathway. PRP can interfere with arrhythmia by regulating the protein level of ADORA1 and the metabolic levels of adenosine and cGMP in the cGMP-PKG pathway. The above findings indicated that PRP is a Chinese herbal medicine with great potential for preventing and reducing the occurrence of arrhythmias.

## Conclusion

This study used network pharmacology and metabolomics correlation analysis to explain the mechanism of PRP in arrhythmia. The results showed that PRP can significantly improve barium chloride-induced zebrafish cardiac congestion, shorten SV-BA spacing, and reduce myocardial apoptosis. By upregulating the expression level of the ADORA1 target and the metabolic levels of adenosine and cGMP in the cGMP-PKG signalling pathway, PRP plays a role in reducing arrhythmia. Therefore, we believe that PRP shows great potential as an adjuvant for the treatment of arrhythmia.

## Data Availability

The raw data supporting the conclusions of this article will be made available by the authors, without undue reservation, to any qualified researcher.

## Abbreviations

ADORA1, Adenosine A1 Receptor; AO, acridine orange; BA, bulbus arteriosus; BP, biological processes; CC, cellular components; CT, cycle threshold; FA, formic acid; GO, gene Ontology; IP3R, inositol triphosphate receptor; MF, molecular functions NOS, nitric oxide synthase; PBS, phosphate-buffered saline; PI3K/Akt, phosphoinositide 3-kinase/protein kinase B; PLS, partial least squares; PPI, protein-protein interaction; PRP, *Poria cum Radix Pini*; PRP-H, PRP high-dose group; PRP-L, PRP low-dose group; PRP-M, PRP medium-dose group; RT-PCR, reverse transcription polymerase chain reaction; SPSS, statistical program for the social sciences; SV, sinus venosus; UPLC/Q-TOF-MS, ultraperformance liquid chromatography/quadrupole time-of flight mass spectrometry; VIP, variable importance in projection.
