# Supplementary material for: Poria cum Radix Pini Rescues Barium Chloride-Induced Arrhythmia by Regulating the cGMP-PKG Signalling Pathway Involving ADORA1 in Zebrafish
Source: Front Pharmacol. 2021 Jul 30;12:688746. doi: 10.3389/fphar.2021.688746 (PMC8360851; doi:10.3389/fphar.2021.688746)
Supplement: Supplementary file 1 [file DataSheet1.pdf]

SUPPLEMENTARY MATERIAL

Poria cum Radix Pini Rescues Barium chloride-induced Arrhythmia by Regulating the ADORA1-Involved cGMP-PKG Signalling Pathway in Zebrafish

*Ning-Juan Yang [1†], Yan-Ru Liu [1† *], Zhi-Shu Tang [1*], Jin-Ao Duan [2], Yan-Ya Feng[1], Zhong-Xing Song [1], Ming-Geng Wang[3], Yu-Ru Zhang [1], Bai-Jin Chang [1,4], Meng-Li Zhao [1] and Yan-Ting Zhao [1]*

[1] Shaanxi Province Key Laboratory of New Drugs and Chinese Medicine Foundation Research, Shaanxi Collaborative Innovation Center Medicinal Resources Industrialization, Shaanxi University of Chinese Medicine, 712083, Xianyang, P.R. China

[2] Key Laboratory for High Technology Research of TCM Formulae and Jiangsu Collaborative Innovation Center of Chinese Medicinal Resources Industrialization, Nanjing University of Chinese Medicine, 210023, Nanjing, P.R. China

[3] Shandong Buchang Pharmaceutical Co. Ltd, 250000, Shandong, P.R. China

[4] Changchun University of Chinese Medicine, 130117, Chang chun, P.R. China

**Corresponding author:*

Yan-Ru Liu, Tel/Fax: +86 029 38182207, Email address: yanzi_2203@aliyun.com.

Zhi-Shu Tang, Tel/Fax: +86 029 38183461, Email address: tzs6565@163.com.

†These authors contributed equally to this paper.

Abstract:

The traditional Chinese medicine *Poria cum Radix Pini* (PRP) is a fungal medicinal material that has been proven to play an important role in the treatment of arrhythmia. However, the mechanism of its effect on arrhythmia is still unclear. In this study, network pharmacology and metabolomics correlation analysis methods were used to determine the key targets, metabolites and potential pathways involved in the effects of PRP on arrhythmia. The results showed that PRP can significantly improve cardiac congestion, shorten the SV-BA interval and reduce the apoptosis of myocardial cells induced by barium chloride in zebrafish. By upregulating the expression of the ADORA1 protein and the levels of adenosine and cGMP metabolites in the cGMP-PKG signalling pathway, PRP can participate in ameliorating

arrhythmia. Therefore, we believe that PRP shows great potential for the treatment of arrhythmia.

Keywords: Arrhythmia; *Poria cum Radix Pini*; ADORA1; cGMP-PKG signalling pathway; Zebrafish.

Supplementary File S1

S1.1 LC_{50} experiment methods

The embryos of 72hpf zebrafish were divided into six groups: control group, model group, verapamil positive control group, PRP-high dose, medium dose and low dose group. The arrhythmia model of zebrafish was established using the chemical drug barium chloride, and verapamil hydrochloride injection was used as the positive control group. sixty zebrafish embryos with normal development after fertilization were taken from each group and placed in 48-well plates. Model group was set at 5 concentrations of 58.3, 52.5, 47.25, 42.5 and 38.25 μg/mL. The positive group was given 5 concentrations of 12.25, 11, 9.9, 8.91 and 6 μg/mL, and the PRP group was given 5 concentrations of 4800, 4320, 3888, 3499 and 3149 μg/mL (2 Wells per concentration, five embryos per well, 2 mL), After 24 h incubation with sterile saline solution of barium chloride, verapamil hydrochloride, or PRP extract, and the survival number of zebrafish embryos was recorded.

S1.2 LC_{50} experiment and results

The LC_{50} of the model group was 42 μg/mL, the positive group was 9 μg/mL, and the PRP group was 4878 μg/mL by regression analysis in SPSS 26.0 software. The drug concentration of model group and positive control group was set at 1/20 LC_{50} (2.1 μg/mL and 0.45 μg/mL). The dose of PRP in high, medium and low administration groups were 1/30 LC_{50}, 1/40 LC_{50}, 1/50 LC_{50} (162.60 μg/mL, 121.95 μg/mL, and 97.56 μg/mL), respectively. Then, zebrafish embryos were randomized divided into six groups and placed on 48-well cell culture plate (ten embryos per group, five embryos per well): control, model (barium chloride sterile saline solution 2.1 μg/mL, 2mL), positive control (model + injection 0.45 μg/mL), and three treatment groups (PRP-H: model + PRP extract 162.60 μg/mL,

PRP-M: model + PRP extract 121.95 μg/mL, PRP-H: model + PRP extract 97.56 μg/mL). All animal handling and experimental conditions were approved by the Laboratory Animal Care and Use Committee of the Shaanxi University of Chinese Medicine.

Supplementary File S2

Metabolomics Analytical validation

S2.1 Sample preparation

To prepare the calibration curve, appropriate amounts of the adenosine and cGMP reference materials were accurately measured, and a 1: 1 methanol: water solution was added to produce a stock solution of 0.1 mg/mL for each reference material. The working standard solution was prepared by continuously diluting the stock solution to obtain the necessary concentration, and all solutions were stored at 4°C until use.

The metabolic sample preparation method was consistent with the nontarget metabolomics sample preparation method.

S2.2 LC-MS analysis

Metabolites were quantified with an AB Q Trap 4500-Agilent 1290 liquid chromatography-electrospray ionization/mass spectrometry (LC-ESI/MS) system with an external standard method. An Infinity Lab Poroshell 120HILIC-Z column (100 mm×2.6 mm, 2.7-Micron with column ID) was used under the following conditions: mobile phase A, 0.2% formic acid in H2O; mobile phase B, acetonitrile; linear elution time, 30 min; gradient elution conditions, 0~1.5 min, 10% B, 1.5~23 min, 10%~99% B, 23~24 min, 99% B, 24~27 min, 99%-10% B, 27~30 min, 90% B. The column temperature was maintained at 30°C. ESI positive and negative ion modes were used for scanning, and the detection mode was multi-reaction monitoring. The ion spray voltages of positive and negative ion modes are 5500 V and 4500 V.TEM at 550°C, GS1 and GS2 were nitrogen with 344.738 kPa (50 psi) and CUR 241.316 kPa (35 psi). Collection parameters for compound ion pair optimization: DP, CE and cell exit potential (CXP), as shown in **Table S1**. All data were analyzed using Analyst Software 1.6.3, and metabolites were quantified using Multiquant Software 2.1.

Table S1 Mass spectrometric information of adenosine and cGMP

Metabolites	Ion mode	t_R/min	parent ion (*m/z*)	daughter ion (*m/z*)	DP/V	CE/eV	CXP/V
adenosine	Positive	0.86	268.1	136.1	235.44	21.99	6.00
cGMP	Negative	1.56	344.1	150.0	-104.5	-29.23	-9.82

S2.3 Method validation

The methodology was investigated from the aspects of precision, limit of detection, limit of quantification, linearity, precision, stability, reproducibility, matrix effect and extraction recovery

S2.3.1 Precision

The metabolite adenosine and cGMP standard were measured for 6 consecutive times, the RSD values of peak area and retention time were 0.02% and 0.08%, respectively. The RSD values of retention time were 0, which indicated that the precision of the instrument was good.

S2.3.2 LOD and LOQ and Linearity

When the signal-to-noise ratio (S/N) of the analyte is greater than 3:1, the detection limit is obtained; when the signal-to-noise ratio (S/N) is greater than 10:1, the quantification limit is obtained. From this definition, the detection limit and quantification limit of adenosine were 3ng/mL and 15ng/mL, respectively, and the detection limit and quantification limit of cGMP were 0.03ng/mL and 0.1ng/mL, respectively. By diluting the QC samples step by step, the linear regression equation of adenosine was Y= 112.58 X +1214, and the correlation coefficient R was 0.9994. The linear regression equation of cGMP was Y = 3445 X +599.45, and the correlation coefficient R was 0.9999, indicating that the two metabolites had a good linear relationship.

S2.3.3 Repeatability

Six QC samples were prepared in parallel, and the RSD values of adenosine and cGMP peak area were 0.10% and 0.01%, respectively, and the RSD values of retention time were 0.02% and 0.01%, respectively, which indicated that the method had good repeatability.

S2.3.4 Stability

In order to determine the stability of the treated samples, the samples were stored in an automatic sampler at 4°C, and the peak areas were measured at 0, 24 and 48h, respectively. The RSD values of the peak areas of adenosine and cGMP were calculated as 8.36% and 2.87%, respectively, and the retention time RSD values were 0 and 2.18%, respectively, which indicated that the samples had good stability.

S2.3.5 Matrix effect and extraction recovery

By adding the mixed standard solution to the zebrafish metabolic sample in the control group after protein removal, and comparing its peak area with the peak area of each standard in the standard solution, the matrix effects of adenosine and cGMP were calculated to be 90.08% and 85.80%, respectively. It showed that the biological matrix of zebrafish metabolism sample didn't affect the determination of the sample. By comparing the peak area of the zebrafish metabolic sample in the control group before deproteinization and the peak area of the mixed standard solution after deproteinization, the extraction recovery rates of adenosine and cGMP were calculated to be 81.07% and 80.49%, respectively, which indicated that the extraction method of this sample was stable and reliable.

Supplementary File S3

S3.1 PRP chemical components identification conditions

The concentration of PRP extract was 9.6 mg/mL. The PRP extract solution was analysed on a Waters Acquity H-CLass ultra-performance LC (Waters, MA, USA) / tandem a 5600^{+} triple-quadruple time-of-flight mass spectrometer (QTOF-MS) system (AB SCIEX, MA, USA) using a Waters BEH C_{18} column (50 mm × 2.1 mm, 1.7 μm). With a gradient mobile phase of 0.1% formic acid aqueous solution (A) and 0.1% formic acid-acetonitrile solution (B) at a flow rate of 0.3 mL/min and 30°C column temperature. The gradient program was as follows: 0~1 min, 2% B; 1~42 min, 2%~100% B; 42~44 min, 100% B; 44~48 min, 100%~2% B; 48~50 min, 2% B. Full scan and MS/MS experiments were performed under positive and negative modes by using an electrospray ionization (ESI) ion source. Information-dependent data (IDA) acquisition mode was used to switch automatically between MS and MS/MS acquisition with the following parameters: ionspray voltage

floating: +5500 V or -4500 V, declustering potential (DP): 40 V, ion source gas 1 and ion source gas 2 both set as nitrogen: 50 psi; curtain gas: 35 psi; source temperature: 500°C. The scanning range of parent ions (TOF-MS) was 50 to 2000 m/z. The eight strongest peaks exceeding 100 cps were collected by MS2, and the subion scanning range was 1000~2000 m/z. Data were acquired using Analyst TF software (version 1.7.1, AB SCIEX). Components identification was performed by using Peakview software (version 2.2, AB SCIEX), and Masterview software (version 1.1, AB SCIEX) coupled with TCM library 1.0 database.

S3.2 UPLC/Q-TOF-MS components identification of PRP

Under the optimized chromatographic and mass spectrometry conditions, UPLC/Q-TOF-MS in positive and negative detection modes was used to analyse the alcohol extract of PRP. The results showed that 31 compounds were identified in positive ion mode and 5 in negative ion mode (**Table S2**). According to the molecular weight, retention time, and MS/MS data of the four compounds analysed using UPLC-TOF-MS chromatography, four compounds of PRP were identified: pachymic acid, glycyrrhetinic acid, oleanolic acid and adenosine **(Figure S1 A-D)** (**Table S3**).

Table S2 Compounds identified by MS in positive/negative ion mode

NO	Name	Molecular Formula	Extraction Mass (Da)	Found at RT(min)	Adduct
1	Pachymic acid	$C_{33}H_{52}O_5$	529.38875	30.31	+H
2	Schisandrin A	$C_{24}H_{32}O_6$	417.22716	25.86	+H
3	Vitamin K1	$C_{31}H_{46}O_2$	451.35707	30.31	+H
4	Alisol B 23-acetate	$C_{32}H_{50}O_5$	515.37312	29.09	+H
5	Hydroxycamptothecine	$C_{20}H_{16}N_2O_5$	365.1132	0.5	+H
6	Glycyrrhetinic acid	$C_6H_{14}O_6$	471.34688	19.85	+H
7	Oleanolic acid	$C_{30}H_{48}O_3$	457.36763	33.02	+H
8	Schisandrin B	$C_{23}H_{28}O_6$	401.19588	27.48	+H
9	Linoleic acid	$C_{18}H_{32}O_2$	281.24751	32.38	+H
10	Schisanhenol	$C_{23}H_{30}O_6$	403.21153	23.67	+H
11	Adenosine	$C_{10}H_{13}N_5O_4$	268.10402	0.56	+H
12	Vitamin A acid	$C_{20}H_{28}O_2$	301.2162	36.54	+H
13	Aristolone	$C_{15}H_{22}O$	219.17434	20.56	+H
14	Kaurenoic acid	$C_{20}H_{30}O_2$	303.23186	30.14	+H
15	Schisandrin	$C_{24}H_{32}O_7$	433.22209	17.7	+H
16	1,2,3,7-tetramethoxyxanthone	$C_{17}H_{16}O_6$	317.10197	17.64	+H
17	Ligustilide	$C_{12}H_{14}O_2$	191.10666	20.42	+H

18	polygalaxanthone III	$C_{25}H_{28}O_{15}$	569.1501	8.36	+H
19	1,2,3,6,7-pentamethoxyxanthone	$C_{18}H_{18}O_7$	347.11253	15.54	+H
20	1,7-dimethoxyxanthone	$C_{15}H_{12}O_4$	257.08082	16.71	+H
21	Glucosamine Hydrochloride	$C_6H_{13}NO_5$	180.08665	0.59	+H
22	Tanshinone IIA	$C_{19}H_{18}O_3$	295.13286	26.3	+H
23	L-Valine	$C_5H_{11}NO_2$	118.08626	0.56	+H
24	Adenine	$C_5H_5N_5$	136.06177	0.6	+H
25	guanosine	$C_{10}H_{13}N_5O_5$	284.09895	0.55	+H
26	Xylitol	$C_5H_{12}O_5$	153.07574	0.58	+H
27	nicotinamide	$C_6H_6N_2O$	123.05529	0.75	+H
28	Tectorigenin	$C_{16}H_{12}O_6$	301.07066	17.67	+H
29	Trigonelline	$C_7H_7NO_2$	138.05496	0.57	+H
30	Polydatin	$C_{20}H_{22}O_8$	391.13875	9.05	+H
31	2-Methoxycinnamic acid	$C_{10}H_{10}O_3$	179.07026	31.2	+H
32	Lobetyolin +HCOOH	$C_{20}H_{28}O_8$. HCOOH	441.17662	27.35	-H
33	2-HYDROXYADENOSINE	$C_{10}H_{13}N_5O_5$	282.0844	0.5	-H
34	Scopolamine Hydrobromide	$C_{17}H_{21}NO_4$	302.13979	46.47	-H
35	Nuciferin	$C_{19}H_{21}NO_2$	294.14995	0.54	-H
36	Peimine	$C_{27}H_{45}NO_3$	430.33266	36.57	-H

A

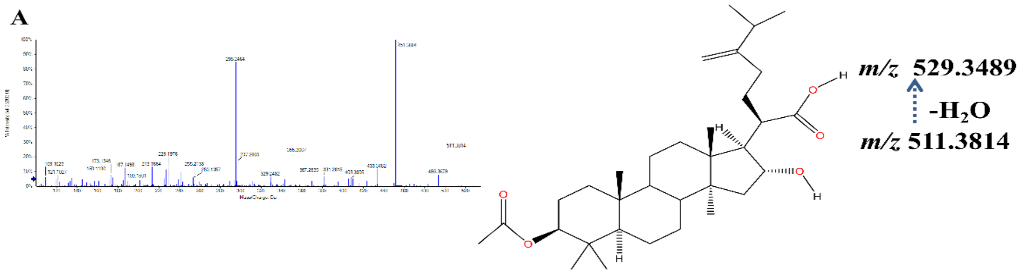

B

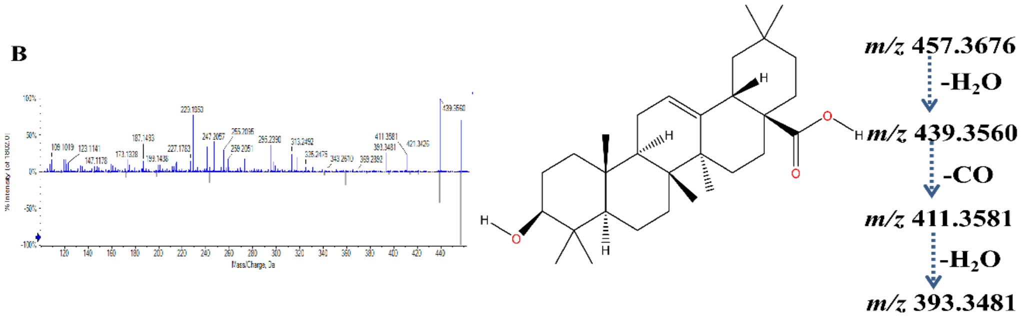

C

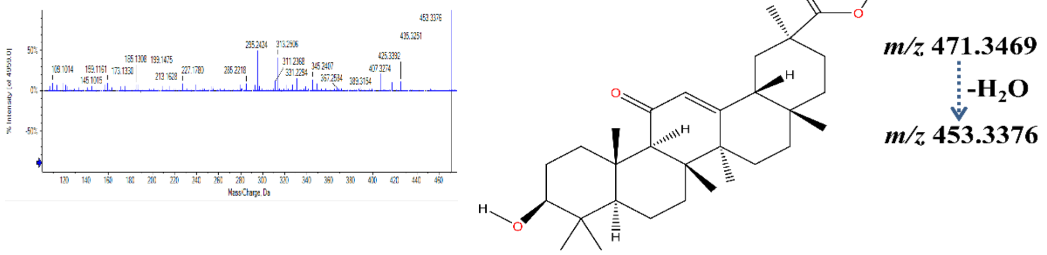

D

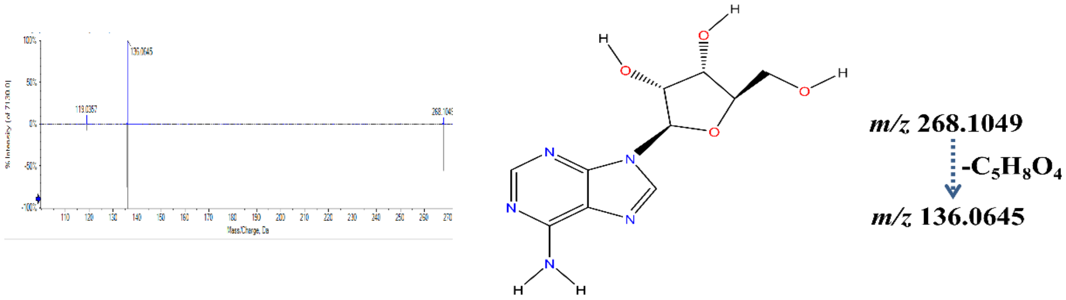

(A) pachymic acid (B) olanolic acid (C) glycyrrhetinic acid (D) adenosine

Figure S1 MS/MS diagram and structural formula of the components contained in the alcohol extract of PRP.

Table S3 Compounds identified by MS2 in positive ion mode

NO.	Name	Molecular Formula	Extraction Mass (Da)	Found at RT(min)	Daughter ion
1	Pachymic acid	$C_{33}H_{52}O_5$	529.38875	30.31	$C_{33}H_{50}O_4$
2	Oleanolic acid	$C_{30}H_{48}O_3$	457.3676	33.02	$C_{30}H_{46}O_2$、$C_{29}H_{46}O$、$C_{30}H_{44}$
3	Glycyrrhetinic acid	$C_{30}H_{46}O_4$	471.34688	19.85	$C_{30}H_{44}O_2$
4	Adenosine	$C_{10}H_{13}N_5O_4$	268.10402	0.56	$C_5H_5N_5$

Supplementary File S4

S4.1 Determination of key target of PRP for treatment of arrhythmia

To further explore PRP multicomponent and multi-target therapy functions, all the 58 targets of the PPI network were mapped to ‘component-disease’ connections, and then the ‘component-disease-target’ network of PRP therapy was constructed by Cytoscape software. Since the node size is proportional to the importance of nodes involved in the pathway (**Table S4**).

Table S4 Compound related targets

NO.	Compound name	Compound related targets
1	Pachymic	PGR,AR,NR3C2,HMGCR,ESR1,PTGER4,NR3C1,ITGB2,PTGER2,PTGER3,PTGF

	Acid	R,ANXA1,ITGAL,CYP19A1,HDAC2,ADH1C,AKR1C2,ALOX5,ANXA1, AR, CES1, COX4I1, COX5A,COX5B, COX6A2, COX6B1, COX6C, COX7A1,COX7B, COX7C,COX8A, EFTUD1, ESR1, ESRRG, FABP6, FECH,GPBAR1, GSTP1, HMGCR,HOXA10,HSD11B1, HSD11B2,HSD3B1,IGHG2, ITGAL, ITGB2, MT-CO1, MT-CO2, MT-CO3, NCOA1,NPPB,NR1H4,NR1I2,NR3C1,NR3C2,PGR,PLA2G1B, PTGS1, VDR
2	Oleanolic acid	SRD5A1,CYP17A1,SLC8A1,F12,PRLR,ESR1,TRPV1,PTGER1,PTGER4,PGR,AR, NR3C1,OPRK1,FADS2,FADS1,PTGER2,PTGS1,PTGER3,PTGS2,ELOVL4,ANXA1,NR3C2,ADH1C, AKR1C1,AKR1C2, AR, CES1, CLEC4E, COX4I1, COX5A,COX5B, COX6A2, COX6B1, COX6C, COX7A1,COX7B, COX7C,COX8A, EFTUD1, ESR1, ESR2,ESRRG, FABP6,IGHG2, GABRA1,GABRA2, GABRA3, GABRA4,GABRA5, GABRA6, GABRB1,GABRB2, GABRB3, GABRD,GABRE,GABRG1, GABRG2, GABRG3, GABRP, GABRQ, GPBAR1,GRIN1, GRIN2A, GRIN2B, GRIN2C, GRIN2D, GRIN3A, GRIN3B,GSTP1, HOXA10, HSD11B1, HSD11B2, HSD17B1, IGHG2, LSS,MT-CO1, MT-CO2,MT-CO3, NCOA2,NFKB1,NFKB2, NPPB, NR1H4,NR1I2,NR1I3,NR3C1, NR3C2, PGR, PLA2G1B, PPARA, PRLR, RORA,SHBG,SIGMAR1, SULT2A1, SULT2B1,VDR
3	Glycyrrhetinic acid	HMOX1,ICAM1,NQO1,HSD11B1,AR,NR3C1,ANXA1,PTGER1,PTGER4,PTGER2,PTGER3,CD300A,KIF14,SRD5A1,CYP17A1,SLC8A1,F12,PRLR,ESR1,TRPV1, PPARG,FFAR1,PGR,OPRK1,FADS2,FADS1,PPARD,PTGS1,ACSL4,PTGS2,ELOVL4,ACSL3,NR3C2,ADH1C, AKR1C1 ,AKR1C2, ALOX5 , ANXA1, AR ,CES1 , COX4I1 , COX5A , COX5B , FECH,COX6B1 ,COX6C ,COX7A1 , COX7B , COX7C , COX8A , EFTUD1 , ESR1 , ESRRG, FABP6 ,FECH ,GPBAR1, GSTP1 , HMGCR, HSD11B1,HSD11B2 , HSD3B1, IGHG2 , ITGAL , ITGB2 , MT-CO1 , MT-CO2, MT-CO3 ,NCOA1 , NCOA2 , NFKB1 , NFKB2 ,NPPB,NR1H4 , NR1I2, NR3C1, NR3C2 , PGR, PLA2G1B, PRLR,PTGS1 , SULT2B1 ,
4	Adenine	ACACB,MTAP,APRT,PECR,SRPK2ACP1,ACACA,FBP1,PRKAB1,ACSS2,ADCY1,ADK,PDE4D,PRKAA1,CREB1,ACSS1,PIM1,PYGL,HINT1,ACSL1,PDE4B,PRKAB2,SRPK1,SRPK3,SHMT1,MTHFD2,MTR,MTHFD1,ALDH1L1,AMT,FBP2,MTFMT,ATIC,PYGM,SHMT,FTCD,MTHFR,PYGB,SCNN1A,KCNJ8,ADRA1A,SCN1A,SCNN1G,HTR2A, COX6A2,ADRA2A,TLR8,ADRB1,ADRA1D,HRH1,HTR7,HRH2,ADRA1B,SCNN1D,ADRA2C,AOC3,P4HA1,TLR7,KCNJ1,AGTR1,ADRB3,SCNN1B,CYP2B6,ADRA2B,SLC6A2,SAXO1,SCT,UGCG,PHKG2,APLP1,CHRNA7,PNP,ARRB2,RAB7A,PRKAA2,HTR2C,HSP90AB1,ALDH3A2,INS,CALCOCO2,SLC22A1,AC2,ARRDC3,CALCA,HPRT1,ACTN2,ACACB, ACP1,APRT,MTAP, PECR, PIM1,PKIA, PRKACA,SRPK2
5	Ergotamine	ADRA1A,HTR2A,HTR1F,HTR1A,ADRA1B,ADRA2A,HTR1B,DRD5,DRD1,HTR2C,ADRA1D,ADRA2B,HTR1D,DRD2,SLC6A2,SLC6A3,DNMT3A,CNR1,INS,SLC22A1,ADRA2C,PHKG2,APLP1,CHRNA7,CD34,CALCA,DRD4,HTR7,PTGER2, PTGER3,DRD3,HTR1E,PTGER1,PTGER4,SLC6A4,SLC6A1,SLC6A13,SLC6A8,SLC6A11,SLC6A12,SLC6A6,SLC6A7,HTR2B,ADRB2,HTR3D,GABRA2,GABRQ, GABRD,GABRB1,GABRG3,GABRB3,HTR3B,GABRA5,HTR3E,GABRA6,HTR3

		A,GABRA1,HTR6,GABRE,GABRG2,GABRA3,GABRG1,GABRP,SLCO2B1,GABRA4,HTR3C,GABRB2
6	Lauric Aldehyde	SCN11A,SCN2B,ABAT,SCN1A,SCN3B,SCN3A,SCN7A,SCN5A,SCN10A,AKR1D1,ALDH5A1,SCN2A,HDAC9,TYR,SCN9A,SCN4A,SCN4B,SRD5A2,ACADSB,SCN8A,SCN1B,OGDH,HDAC2,ABAT, ABL1, ACADSB,ACOX1, ACSL3, ACSL4, ADH5,ALDH5A1,ARF1, ARF6,C8G, CALM1, CNR1, CNR2, CYP2C8,ECI2,ELOVL4, FADS1,FADS2,FFAR1,HTR4, FURIN, GLTP, GM2A, GUCA1A, HAO1, HDAC2, HDAC9,HNF4A, HNF4G, INS, LALBA, LIP3, LTB4R, LTB4R2,LTF, LY96, NCOA1,NR1H4, OGDH, PAEP,PKIA, PLA2G1B, PLA2G2A,PLA2G2D,PLA2G2E,PMP2,PPARA, PPARD, PPARG, PPA_RS05235, PPP3CA,PPP3R1,PPT1, PRKACA,PTGS1,PTGS2, PVR, RCVRN,RHO, RXRA, RXRB, RXRG ,S100B,SCN10A,SCN11A, SCN1A, SCN1B, SCN2A, SCN2B, SCN3A, SCN3B, SCN4A, SCN4B,SCN5A, SCN7A, SCN8A, SCN9A, SEC14L2, SLC8A1, SREBF1,TLR4, TM1468,TRAPPC3,TRPV1,VLDLR
7	Adenosine	ADORA2A,ADORA3,ADORA1,ADORA2B,SLC25A6,SLC25A5,TACR2,RIPK1,CX3CR1,ADA,HAP1,ABCC2,AKT1,ACVR1,ASNA1,ADRBK2,ACSS2,PRKAA1,ABCC9,ACSS1,ABL1,AFG3L2,ACVRL1,ADCY1,CDK15,NT5C2,AMHR2,TNK2,ASS1,ASNS,ACVR1B,ACSL1,ABCA1,ABL2,ADRBK1,NAE1,APAF1,ABCC8,ALK,ABCB11,ABCB1,ABCG1,ARAF,FKBP1A,TYR,TYMS,FBP1,PRKAB1,RRM2,POLE,CREB1,PIM1,PYGL,POLE2,POLE3,HINT1,POLA1,RRM2B,ADK,PDE4B,PNP,PRKAB2,POLE4,RRM1,PDE4D,CACNA1C,CACNB1,CACNA1D,SLC6A4,CACNB3,KCNH2,CACNA1F,AKT3,CACNA1A,ABCB4,CACNA1I,CACNA1G,SCN5A,CACNB2,KCNJ11,CACNA1S,PRKAA2,CACNB4,AKT2,CACNA1B,CBSL,MAT1A,CBS,COMT,DCK,MAT2A,AMD1,GNMT,PDE5A,UTS2R,HMGCR,DGKI,FURIN,UTS2,INS,KHDRBS1,SLC18A3,CRH,ATP1A2,HOMER1,SHANK3,AVPR2,SPX,ATP2A1,SMO,ADRA2A,CETN2,CRP,CETN1,PIK3CG,TNF,IDO1,AGT,KCNB1,DRD1,SORCS3,LTA,SLC44A4,GHRL,NCBP2,PCSK6,DRD2,DNM3,CHRNA3,SCN9A,PTGDR2,PLAT,PTGR2,CFTR,PTGER2,PTGS1,PLA2G2A,THBD,PTGS2,GLO1,PPARG,PTGER1,PPARA,BCL2,FABP2,TPK1,SLC19A2,TOP2A,DHFR,TOP2B,SLC6A3,PDE7B,PDE7A,PDE9A,PECR,ADRA1A,PDE4A,RYR1,SLC18A2,ITPR3,SLC12A2,MAPK1,DRD4,PDE4C,PDE3A,HTR7,SLC12A7,SLC12A6,SLC12A5, ADRB2,HTR1A,ADRA1B,ACACB,MTAP,ITPR1,ADRA2C,DBH,PDE6A,PTGER3,PDE10A,PDE1C,SLC25A31,DRD3,PRKDC,PIK3CD,HTR1B,ACSL5,DRD5,ATM, SRPK2,PDE3B,SLC12A1,PTGIS,PDE6B,PIK3CA,ACVR1C,PIK3R3,PDE2A,PIK3R2,ADRB1,PIK3R1,PDE1A,PTGER4,APRT,ADRB3,TGFBR1,PDE8B,SLC18A1,PIK3CB,ACP1,SLC12A4,ABCG4,ADRA1D,PDE6C,ITPR2,ADRA2B,PDE11A,P2RY12,PTGIR,PDE1B,PAH,SLC6A2,PDE8A,CACNG1,ACSL6,CUBN,TCN1,AMN, MUT,MMACHC,MMAB,MTRR,POLB,MMAA,DNMT1,MTR,FBP2,ATF4,SAXO1,XBP1,AK9,RALA,BICD1,UGCG,JAG1,ASNSD1,SMAD3,CASP9,TGFBR2,CTNNB1,VLDLR,ABCA7,UBA3,GRB2,ABCA1,ABCB1, ABCB11, ABCC1, ABCC2, ABCC4, ABCC6 , ABCC8, ABCC9, ABCG1, ABL1, ABL2, ACSL1, ACSS1, ACSS2, ACTA1, ACVR1, ACVR1B, ACVRL1, ADA, ADCY1, ADH1C, ADK, ADORA1, ADORA2A, ADORA2B, ADORA3, ADRBK1, ADRBK2, AFG3L2, AHCY, K1, AK2, AK8, AKT1, ALK, AMHR2, APAF1, ARAF, ASNA1, ASNS,

		ASS1, BAG1, BALF5, CCT3, CDK15, CFTR, COMTD1, CREB1, CSNK1G2, CSNK2A1, CSNK2B, DAM, DAPK1, DCK, DNMT1, DPH5, DTYMK, EEF2, ENPP1, EPHA2, EPHB2, FBP1, FHIT, GALK1, GAMT, GAPDH, GAPDHS, GNMT, GSG2, GSK3B, GSS, HCN2, HINT1, HK1, HNMT, HSP90B1, HSPA8, IGF1R, IMPDH1, IMPDH2, ITPKA, KIF1A, KIF2C, L, LCK, LSM6, MAFF_RS13750, MAPK10, MAPK12, MAPK3, MTAP, NAE1, NME1, NT5C2, PAPS, PB1, PCMT1, PDE4B, PDE4D, PFKFB4, PIK3CA, PIM1, PNMT, PNP, POLA1, POLB, POLE, POLE2, POLE3, POLE4, PPP5C, PRKAA1, PRKAB1, PRKAB2, PRKAR1A, PRKAR2B, PRMT1, PRMT3, PYGL, RAD51, RNASE1, RPL10L, RPL11, RPL13A, RPL15, RPL19, RPL23, RPL23A, RPL26L1, RPL3, RPL37, RPL8, RRM1, RRM2, RRM2B, RSL24D1, SETD7, SIRT3, SIRT5, SLC25A4, SRPK2, STM4066, TK, TLR7, TM0588, TNK2, TOP2B, TRDMT1, TRPM7, TTHA0667, TTHA1435, UCK2, VCP, PTGS1, PNP, PTGS2, PTPN1, ADORA2A
8	palmitic acid	CTSD,ADH1B,ADH1C,PTGS1,PTGS2,NCOA1,BCL2,IL10,TNF,,PTEN,GUSBP3, SLC22A5,LALBA,PPT1,PAEP,CYP2C,PMP2,SEC14L2,RHO,TRAPPC3,HNF4G,AKR1D1,TYR,SRD5A2,SCN11A,SCN2B,ABAT,SCN1A,ESRRG,SCN3B,COX6C,COX5B,COX7C,COX1,SCN3A,AKR1C2,SCN7A,SCN5A,SCN10A,ALDH5A1,SCN2A,COX5A,SLC25A4,AR,COX7A1,FECH,COX4I1,HDAC9,PLA2G1B,COX6A2,SCN9A,SCN4A,SCN4B,ADH1C,COX6B1,FABP6,CES1,ACADSB,SCN8A,COX7B, SCN1B,COX2,OGDH,HDAC2,COX8A,NR1H4,PLD1,PCYT1A,PCYT1B,PHOSPHO1,ACHE,PLD2,BCHE,SUCLG2,SLC13A2,SLC13A1,SUCLG1,HSD17B6,SLC25A10,P3H3,OXCT2,TMLHE,PLOD1,SUCNR1,SLC13A3,ASPH,BBOX1,SDHB,OXCT1,PLOD3,P4HA1,SDHC,P3H2,SDHA,P4HA2,SUCLA2,SDHD,P3H1,DCT,AKR1C3,CRTAP,SLIT2,ACO2,JMJD6,SLC13A4,SDHAF2,SLC1A3,SALL1,HSD17B11, TYRP1,HIF1AN,UROS,SLC13A5,P4HB,CACNA2D1,PLAT,SLC7A2,CACNA1A, GRIN3B,CACNA2D2,GRIN2A,TOP1,PLG,CACNA1B,GRIN2C,GRIN2B,SLC7A3,KARS,GRIN3A,SLC7A1,SLC7A4,GRIN2D,ADORA1,GRIN1,ABAT,ABL1, ACADSB,ACOX1, ACSL3, ACSL4,ADH5, ALDH5A1, ARF1, ARF6,C8G CALM1, CNR1 CNR2, CYP2C8, ECI2,ELOVL4, FADS1,FADS2, FFAR1,FKBP1A,FURIN, GLTP, GM2A, GUCA1A, HAO1,HDAC2,HDAC9,HNF4A, HNF4G, INS, LALBA, LIP3, LTB4R, LTB4R2,LTF, LY96,NCOA1, NR1H4, OGDH, PAEP, PKIA,PLA2G1B,PLA2G2A, PLA2G2D, PLA2G2E, PMP2,PPARA,PPARD, PPARG, PPA_RS05235,PPP3CA, PPP3R1, PPT1, PRKACA, PTGS1,PTGS2,PVR, RCVRN, RHO,RXRA, RXRB, RXRG, S100B, SCN10A, SCN11A, SCN1A, SCN1B, SCN2A,SCN2B, SCN3A, SCN3B, SCN4A, SCN4B,SCN5A, SCN7A, SCN8A,SCN9A,SEC14L2, SLC8A1, SREBF1,TLR4,TM1468,TRAPPC3,TRPV1,VLDLR
9	lauric acid	PTGS1,BCHE,PLA2G7,ADH1B,ADH1C,ELOVL1,PTGS2,,RELA,AKT1,IL6R,,DUOX2,CD80,AKR1D1,TYR,SRD5A2,SCN11A,SCN2B,ABAT,SCN1A,ESRRG,SCN3B,COX6C,COX5B,COX7C,COX1,SCN3A,AKR1C2,SCN7A,SCN5A,SCN10A,ALDH5A1,SCN2A,COX5A,COX3,AR,COX7A1,FECH,COX4I1,HDAC9,PLA2G1B,COX6A2,COX3,SCN4A,SCN4B,ADH1C,COX6B1,FABP6,CES1,ACADSB,SCN8A, COX7B,SCN1B,COX2,OGDH,HDAC2,COX8A,NR1H4,PLD1,PCYT1A,PCYT1B,

		PHOSPHO1,ACHE,PLD2,BCHE,SUCLG2,SLC13A2,SLC13A1,SUCLG1,HSD17B6,SLC25A10,P3H3,OXCT2,TMLHE,PLOD1,SUCNR1,SLC13A3,ASPH,BBOX1,SDHB,OXCT1,PLOD3,P4HA1,SDHC,P3H2,SDHA,P4HA2,SUCLA2,SDHD,P3H1,DCT,AKR1C3,CRTAP,SLIT2,ACO2,JMJD6,SLC13A4,SDHAF2,SLC1A3,SALL1,HSD17B11,TYRP1,HIF1AN,UROS,SLC13A5,P4HB,CACNA2D1,PLAT,SLC7A2,CACNA1A,GRIN3B,CACNA2D2,GRIN2A,TOP1,PLG,CACNA1B,GRIN2C,GRIN2B,SLC7A3,KARS,GRIN3A,SLC7A1,SLC7A4,GRIN2D,ADORA1,GRIN1,ABAT ,ABL1,ACADSB , ACOX1, ACSL3 , ACSL4,ADH5 ,ALDH5A1, ARF1 , ARF6 ,C8G, CALM1,CNR1, CNR2 , CYP2C8,ECI2 ,ELOVL4 , FADS1 , FADS2 ,FFAR1 , FKBP1A , FURIN , GLTP , GM2A, GUCA1A , HAO1 ,HDAC2 ,HDAC9, HNF4A , HNF4G , INS , LALBA , LIP3 , LTB4R , LTB4R2 , LTF,LY96, NCOA1, NR1H4 , OGDH, PAEP , PKIA , PLA2G1B , PLA2G2A, PLA2G2D , PLA2G2E , PMP2 , PPARA , PPARD, PPARG , PPA_RS05235 , PPP3CA, PPP3R1 , PPT1 , PRKACA,PTGS1 , PTGS2 ,,PVR,RCVRN,RHO , RXRA ,RXRB, RXRG ,S100B, SCN10A , SCN11A, SCN1A , SCN1B ,SCN2A ,SCN2B , SCN3A , SCN3B,SCN4A ,SCN4B ,SCN5A , SCN7A ,SCN8A , SCN9A , SEC14L2 , SLC8A1 ,SREBF1 ,TLR4, TM1468 ,TRAPPC3 , TRPV1,VLDLR
10	caprylic acid	PLA2G7,ADH1B,ADH1C,PPARA,EP300,FABP1,RBP2,AKR1D1,TYR,SRD5A2,SCN11A,SCN2B,ABAT,SCN1A,ESRRG,SCN3B,COX6C,COX5B,COX7C,COX1,SCN3A,AKR1C2,SCN7A,SCN5A,SCN10A,ALDH5A1,SCN2A,COX5A,COX3,AR,COX7A1,FECH,COX4I1,HDAC9,PLA2G1B,COX6A2,SCN9A,SCN4A,SCN4B,ADH1C,COX6B1,FABP6,CES1,SCN9A,SCN8A,COX7B,SCN1B,COX2,OGDH,HDAC2,COX8A,NR1H4,PLD1,PCYT1A,PCYT1B,PHOSPHO1,ACHE,PLD2,BCHE,SUCLG2,SLC13A2,SLC13A1,SUCLG1,HSD17B6,SLC25A10,P3H3,OXCT2,TMLHE,PLOD1,SUCNR1,SLC13A3,ASPH,BBOX1,SDHB,OXCT1,PLOD3,P4HA1,SDHC,P3H2,SDHA,P4HA2,SUCLA2,SDHD,P3H1,DCT,AKR1C3,CRTAP,SLIT2,ACO2,JMJD6,SLC13A4,SDHAF2,SLC1A3,SALL1,HSD17B11,TYRP1,HIF1AN,UROS,SLC13A5,P4HB,CACNA2D1,PLAT,SLC7A2,CACNA1A,GRIN3B,CACNA2D2,GRIN2A,TOP1,PLG,CACNA1B,GRIN2C,GRIN2B,SLC7A3,KARS,GRIN3A,SLC7A1,SLC7A4,GRIN2D,ADORA1,ABAT, ABL1, ACADSB,ACOX1, ACSL3, ACSL4, ADH5,ALDH5A1,ARF1, ARF6,C8G, CALM1, CNR1, CNR2, CYP2C8,ECI2,ELOVL4, FADS1,FADS2,FFAR1,FKBP1A, FURIN, GLTP, GM2A, GUCA1A, HAO1, HDAC2, HDAC9,HNF4A, HNF4G, INS, LALBA, LIP3, LTB4R, LTB4R2,LTF, LY96, NCOA1,NR1H4, OGDH, PAEP,PKIA, PLA2G1B, PLA2G2A,PLA2G2D,PLA2G2E,PMP2,PPARA, PPARD, PPARG, PPA_RS05235, PPP3CA,PPP3R1,PPT1, PRKACA,PTGS1,PTGS2, PVR, RCVRN,RHO, RXRA, RXRB, RXRG ,S100B,SCN10A,SCN11A, SCN1A, SCN1B, SCN2A, SCN2B, SCN3A, SCN3B, SCN4A, SCN4B,SCN5A, SCN7A, SCN8A, SCN9A, SEC14L2, SLC8A1, SREBF1,TLR4, TM1468,TRAPPC3,TRPV1,VLDLR
11	Tumulosic Acid	NR3C1,SRD5A1,CYP17A1,SLC8A1,F12,PRLR,ESR1,TRPV1,PTGER1,PTGER4,PGR,AR,PTGIR,OPRK1,FADS2,FADS1,PTGER2,PTGS1,PTGER3,PTGS2,ELOVL4,PTGFR,ANXA1,NR3C2,ADH1C,AKR1C1, AKR1C2, ANXA1, AR, CES1,CLEC4E, COX4I1, COX5A, COX5B, COX6A2, COX6B1, COX6C, COX7A1, COX7B,COX7C, COX8A, CYP27B1, EFTUD1,ACADSB, ESRRG,

	FABP6, FECH,GPBAR1, GSTP1, HOXA10,HSD11B1, HSD11B2,HSD3B1,IGHG2, LSS,MT-CO1, MT-CO2, MT-CO3, NCOA1,NCOA2, NFKB1,NFKB2, NPPB, NR1H4,NR1I2, NR1I3, NR3C1, NR3C2, PGR, PLA2G1B, PRLR, RORA, RXRA, SULT2B1, VDR

Table S5 Arrhythmia related targets from three databases.

Database	Arrhythmia related targets
TTD	ADORA1,F2,F10,PLG,HTR4,ADRB2,ABCC8,CD20,PTAFR,VKORC1,KCNH2,SCN5A,ADORA2B,ADRA1A,ADRA2A,ADRA2B,ADRA2C,ADRB1,F8,RFWD2,EDNRA,EDNRB,IL12B,CACNA1C,KCNA5,ADRA1B,ADRA1D,CALM,SPT,ATPase,KCND3,SCN11A,IL18,SLC9A1,TBXAS1,MCHR1,SLC9A3,CACNA1D,HSP70,HSP27,NaC,ADOR,CaC,HCN,,KCNN,GJP,IC,LISC,SLC
ETCM	A2ML1, ACTC1, ATP6,BRAF, CACNA1B, CASR, CAV3,COX3, COX7B, CPT1A, CPT2, CREBBP,CTLA4,CYTB,DES,DMD,EFEMP2,ELN,EP300,ERCC6, ERCC8,FBLN5,FBXL4, FHL1,FOXC2,GATA1,GDAP1,GJA1,GLA,GNA11,GNAQ,GPC3,GPC4,GPX4,GTPBP3, HBB,HCCS,HDAC4,HFE,HJV,HLA-DPB1,HMBS,HRAS,KC,KAT6B,KCNK3,ECP2, MEFV,MGME1,MTO1,MYH6,MYH7,MYLK2,NAA10,ND1,ND2,ND4,ND4L,ND5,ND6, NDUFB11,NKX2-5,NRAS,PCCA,PCCB,PEX1,PEX10,PEX11B,PEX12,PEX13,PEX14, PEX16, PEX19,PEX2,PEX26,PEX3(HPO), PEX5, PEX6 ,PEX7 ,PHOX2B,PHYH, PIK3CA, POLG2,PRDM16,PRTN3,PSMB8,PTPN11,PTPN22,RAF1,RASA2,RIT1,RPL11, RPL15, RPL18, RPL26,RPL27,RPL35,RPL35A,RPL5,RPS10,RPS17,RPS19,RPS24,RPS26, RPS27, RPS28, RPS29, RPS7, RRAS, SALL4, SCN1B, SCN4A, SCN5A, SCNN1B, SCNN1G , SLC19A2,SLC40A1,SOS1,SOS2,SOX10,SPECC1L,SYNE2,TAB2,TAZ,TBX3,TMEM70, TNXB, TRNE,TRNK,TRNL1,TRPM4, TSC1,TSC2, TSR2, TTN ,TTPA,TTR,VHL
Genecards	SCN5A,KCNH2,KCNQ1,ANK2,RYR2,KCNJ2,KCNE2,CACNA1C,KCNE1,LMNA,PKP2, MYH7,DSP,AKAP9,SNTA1,TTN,CAV3,CASQ2,SCN4B,DSG2,KCNJ5,JUP,TMEM43,HCN4,TANGO2,CACNB2,MYBPC3,SCN1B,TNNT2,MYH6,DSC2,GJA5,DMD,NKX2-5,TRDN,RANGRF,NPPA,TNNI3,CACNA2D1,GJA1,GNB5,SCN3B,KCNA5,HSPG2,PLN, NOS1AP,KCND3,KCNJ8,KCNE3,DES,TGFB3,GPD1L,LDB3,CALM2,PRKAG2,MYPN,RBM20,CALM1,ABCC9,SCN2B,RYR1,GATA4,CPT2,TECRL,CACNA1S,ACTN2,SYNE2,TRPM4,SLC25A20,NEXN,KCNE5,SCN10A,TPM1,CACNA1D,DTNA,ACTC1,SYNE1,TAZ,CTNNA3,LAMP2,TTR,LAMA4,LAMA2,GATA6,TCAP,ACADVL,CALM3,TNNC1,SDHA,GYG1,GATA5,PPARG,FLNC,ACE,CSRP3,HFE,GAA,HADHA,FHL1,NUP155,AKAP10, MYL3,CAVIN1,SLMAP,RAF1,SCN4A,IFNG,DPP6,PTPN11,TBX5,XK,VCL,PRDM16,TNNI3K,MYL4,NPPB,GATAD1,HLA-DRB1,BAG3,LOC110121269,KCNQ1-AS1,SLC25A4, MECP2,DMPK,FOS,ACADM,ALG10B,MT-TL1,EYA4,ADRB1,BMP2,CPT1A,TBX3,ANKRD1,KCNQ1OT1,CTLA4,DNAJC19,MYOT,MYLK2,NAA10,MT-ND4,MT-ND5,SGCD, ELN,CRYAB,CAV1,TP53,TBX20,PITX2,FKBP1B,POLG,TWNK,MT-CYB,KCNJ18,SOS1, MYL2,IL6,KCNJ11,CRP,MT-CO1,MT-TK,ERCC6,ACADL,POLG2,CACNA1C-AS1,APOE,HRAS,AGPAT2,BSCL2,TMEM70,HJV,BVES,MT-ND1,FKTN,SLC25A35,GLA,GSN,PEX1,NR2F2,NDUFS2,TNF,TMPO,AGTR1,BRAF,NDUFB11,SGO1,ALB,PIK3CA,HADHB, SLC19A2,RRM2B,SCNN1A,PEX6,TTPA,MT-ATP6,JPH2,ATFB2,SDHB,TOP3A,PHYH,PEX7,CNBP,KRAS,ATFB1,ATFB5,SLC40A1,PSEN1,REN,MT-CO3,FBLN5,PEX14,PEX10,

	PEX2,PEX3,PEX5,PEX26,PEX12,PEX13,PEX16,EDN1,MYOZ2,PDGFRA,DYSF,KCND2,HLA-B,ADRB2,COL4A1,CLCNKB,PSEN2,PPCS,CIZ1,PSMB8,NRAS,CASR,GATA1,HBB,PRTN3,ERCC8,INS,FBN1,MT-CO2,CDH23,SLC12A3,CD2AP,SLC26A4,MT-ND6,MT-ND4L,MEFV,COL1A1,HAND2,RNF207,CHKB,RET,SDHC,EHMT1,ETFDH, PTH,FBXL4

Table S6 58 Common targets from compounds-disease sets

#	SYMBOL	ENTREZID
1	COX7B	1349
2	MT-CO1	4512
3	MT-CO2	4513
4	MT-CO3	4514
5	NPPB	4879
6	PPARG	5468
7	SCNN1A	6337
8	KCNJ8	3764
9	ADRA1A	148
10	SCNN1G	6340
11	ADRB2	154
12	ADRA2A	150
13	ADRB1	153
14	ADRA1D	146
15	ADRA1B	147
16	ADRA2C	152
17	AGTR1	185
18	SCNN1B	6338
19	ADRA2B	151
20	INS	3630
21	ACTN2	88
22	HTR4	3360
23	SCN11A	11280
24	SCN2B	6327
25	SCN3B	55800
26	SCN5A	6331
27	SCN10A	6336
28	SCN4A	6329
29	SCN4B	6330
30	SCN1B	6324
31	CALM1	801
32	ADORA1	134
33	ADORA2B	136
34	ABCC9	10060
35	ABCC8	6833
36	SLC25A4	291
37	RRM2B	50484

38	CACNA1C	775
39	CACNA1D	776
40	KCNH2	3757
41	CACNB2	783
42	KCNJ11	3767
43	CACNA1S	779
44	CACNA1B	774
45	CRP	1401
46	TNF	7124
47	SLC19A2	10560
48	RYR1	6261
49	PIK3CA	5290
50	RPL11	6135
51	RPL15	6138
52	COX3	4514
53	SDHB	6390
54	SDHC	6391
55	SDHA	6389
56	CACNA2D1	781
57	PLG	5340
58	EP300	2033

Table S7 Protein to protein interaction among the 58 targets

#clustering method	cluster number	cluster color	gene count	protein name	protein identifier	protein description
kmeans	1	Red	25	ABCC8	9606.ENSP00000303960	ATP-binding cassette sub-family C member 8; Subunit of the beta-cell ATP-sensitive potassium channel (KATP). Regulator of ATP-sensitive K(+) channels and insulin release; ATP binding cassette subfamily C
kmeans	1	Red	25	ADORA1	9606.ENSP00000356205	Adenosine receptor A1; Receptor for adenosine. The activity of this receptor is mediated by G proteins which inhibit adenylyl cyclase; Belongs to the G-protein coupled receptor 1 family
kmeans	1	Red	25	ADORA2B	9606.ENSP000003045	Adenosine receptor A2b;

					01	Receptor for adenosine. The activity of this receptor is mediated by G proteins which activate adenylyl cyclase
kmeans	1	Red	25	ADRA1A	9606.ENSP00000369960	Alpha-1A adrenergic receptor; This alpha-adrenergic receptor mediates its action by association with G proteins that activate a phosphatidylinositol-calcium second messenger system. Its effect is mediated by G(q) and G(11) proteins. Nuclear ADRA1A-ADRA1B heterooligomers regulate phenylephrine(PE)-stimulated ERK signaling in cardiac myocytes; Belongs to the G-protein coupled receptor 1 family. Adrenergic receptor subfamily. ADRA1A sub-subfamily
kmeans	1	Red	25	ADRA1B	9606.ENSP00000306662	Alpha-1B adrenergic receptor; This alpha-adrenergic receptor mediates its action by association with G proteins that activate a phosphatidylinositol-calcium second messenger system. Its effect is mediated by G(q) and G(11) proteins. Nuclear ADRA1A-ADRA1B heterooligomers regulate phenylephrine (PE)-stimulated ERK signaling in cardiac myocytes; Belongs to the G-protein coupled receptor 1 family. Adrenergic

						receptor subfamily. ADRA1B sub-subfamily
kmeans	1	Red	25	ADRA1D	9606.ENSP00000368766	Alpha-1D adrenergic receptor; This alpha-adrenergic receptor mediates its effect through the influx of extracellular calcium; Adrenoceptors
kmeans	1	Red	25	ADRA2A	9606.ENSP00000280155	Alpha-2A adrenergic receptor; Alpha-2 adrenergic receptors mediate the catecholamine-induced inhibition of adenylate cyclase through the action of G proteins. The rank order of potency for agonists of this receptor is oxymetazoline > clonidine > epinephrine > norepinephrine > phenylephrine > dopamine > p-synephrine > p-tyramine > serotonin = p-octopamine. For antagonists, the rank order is yohimbine > phentolamine = mianserine > chlorpromazine = spiperone = prazosin > propanolol > alprenolol = pindolol; Adrenoceptors
kmeans	1	Red	25	ADRA2B	9606.ENSP00000480573	Alpha-2B adrenergic receptor; Alpha-2 adrenergic receptors mediate the catecholamine-induced inhibition of adenylate cyclase through the action of G proteins. The rank order of potency for agonists of this receptor is clonidine > norepinephrine > epinephrine =

						oxymetazoline > dopamine > p-tyramine = phenylephrine > serotonin > p-synephrine / p-octopamine. For antagonists, the rank order is yohimbine > chlorpromazine > phentolamine > mianserine > spiperone > prazosin > alprenolol > propanolol > pindolol; Belongs to the G-protein coupled receptor 1 family. Adrenergic receptor subfamily. [...]
kmeans	1	Red	25	ADRA2C	9606.ENSP00000386069	Alpha-2C adrenergic receptor; Alpha-2 adrenergic receptors mediate the catecholamine-induced inhibition of adenylate cyclase through the action of G proteins; Adrenoceptors
kmeans	1	Red	25	ADRB1	9606.ENSP00000358301	Beta-1 adrenergic receptor; Beta-adrenergic receptors mediate the catecholamine-induced activation of adenylate cyclase through the action of G proteins. This receptor binds epinephrine and norepinephrine with approximately equal affinity. Mediates Ras activation through G(s)-alpha- and cAMP-mediated signaling; Belongs to the G-protein coupled receptor 1 family. Adrenergic receptor subfamily. ADRB1 sub-subfamily
kmeans	1	Red	25	ADRB2	9606.ENSP000003053	Beta-2 adrenergic receptor;

					72	Beta-adrenergic receptors mediate the catecholamine-induced activation of adenylate cyclase through the action of G proteins. The beta-2-adrenergic receptor binds epinephrine with an approximately 30-fold greater affinity than it does norepinephrine; Belongs to the G-protein coupled receptor 1 family. Adrenergic receptor subfamily. ADRB2 sub-subfamily
kmeans	1	Red	25	AGTR1	9606.ENSP00000419422	Type-1 angiotensin II receptor; Receptor for angiotensin II. Mediates its action by association with G proteins that activate a phosphatidylinositol-calcium second messenger system
kmeans	1	Red	25	CACNA1B	9606.ENSP00000360423	Voltage-dependent N-type calcium channel subunit alpha-1B; Voltage-sensitive calcium channels (VSCC) mediate the entry of calcium ions into excitable cells and are also involved in a variety of calcium-dependent processes, including muscle contraction, hormone or neurotransmitter release, gene expression, cell motility, cell division and cell death. The isoform alpha-1B gives rise to N-type calcium currents. N-type calcium channels belong to the 'high-voltage activated' (HVA) group and are blocked by

						omega-conotoxin-GVIA (omega-CTx-GVIA) and by omega-agatoxin- IIIA (omega-Aga-IIIA). Th [...]
kmeans	1	Red	25	CACNA1C	9606.ENSP00000266376	Voltage-dependent L-type calcium channel subunit alpha-1C; Voltage-sensitive calcium channels (VSCC) mediate the entry of calcium ions into excitable cells and are also involved in a variety of calcium-dependent processes, including muscle contraction, hormone or neurotransmitter release, gene expression, cell motility, cell division and cell death. The isoform alpha-1C gives rise to L-type calcium currents. Long-lasting (L-type) calcium channels belong to the 'high-voltage activated' (HVA) group. They are blocked by dihydropyridines (DHP), phenylalkylamines, benzothiazepines, and by o [...]
kmeans	1	Red	25	CACNA1D	9606.ENSP00000288139	Voltage-dependent L-type calcium channel subunit alpha-1D; Voltage-sensitive calcium channels (VSCC) mediate the entry of calcium ions into excitable cells and are also involved in a variety of calcium-dependent processes, including muscle contraction, hormone or neurotransmitter release, gene expression, cell motility, cell division and

						cell death. The isoform alpha-1D gives rise to L-type calcium currents. Long-lasting (L-type) calcium channels belong to the 'high-voltage activated' (HVA) group. They are blocked by dihydropyridines (DHP), phenylalkylamines, benzothiazepines, and by o [...]
kmeans	1	Red	25	CACNB2	9606.ENSP00000320025	Voltage-dependent L-type calcium channel subunit beta-2; The beta subunit of voltage-dependent calcium channels contributes to the function of the calcium channel by increasing peak calcium current, shifting the voltage dependencies of activation and inactivation, modulating G protein inhibition and controlling the alpha-1 subunit membrane targeting
kmeans	1	Red	25	CRP	9606.ENSP00000255030	C-reactive protein; Displays several functions associated with host defense: it promotes agglutination, bacterial capsular swelling, phagocytosis and complement fixation through its calcium-dependent binding to phosphorylcholine. Can interact with DNA and histones and may scavenge nuclear material released from damaged circulating cells; Short pentraxins
kmeans	1	Red	25	EP300	9606.ENSP00000263253	Histone acetyltransferase p300; Functions as histone acetyltransferase and

						regulates transcription via chromatin remodeling. Acetylates all four core histones in nucleosomes. Histone acetylation gives an epigenetic tag for transcriptional activation. Mediates cAMP-gene regulation by binding specifically to phosphorylated CREB protein. Mediates acetylation of histone H3 at 'Lys-122' (H3K122ac), a modification that localizes at the surface of the histone octamer and stimulates transcription, possibly by promoting nucleosome instability. Mediates acetylation of histone H3 at 'Lys-27' (H3K [...]
kmeans	1	Red	25	HTR4	9606.ENSP00000353915	5-hydroxytryptamine receptor 4; This is one of the several different receptors for 5-hydroxytryptamine (serotonin), a biogenic hormone that functions as a neurotransmitter, a hormone, and a mitogen. The activity of this receptor is mediated by G proteins that stimulate adenylate cyclase; 5-hydroxytryptamine receptors, G protein-coupled
kmeans	1	Red	25	INS	9606.ENSP00000380432	Insulin; Insulin decreases blood glucose concentration. It increases cell permeability to monosaccharides, amino acids and fatty acids. It

						accelerates glycolysis, the pentose phosphate cycle, and glycogen synthesis in liver
kmeans	1	Red	25	KCNJ11	9606.ENSP00000345708	ATP-sensitive inward rectifier potassium channel 11; This receptor is controlled by G proteins. Inward rectifier potassium channels are characterized by a greater tendency to allow potassium to flow into the cell rather than out of it. Their voltage dependence is regulated by the concentration of extracellular potassium; as external potassium is raised, the voltage range of the channel opening shifts to more positive voltages. The inward rectification is mainly due to the blockage of outward current by internal magnesium. Can be blocked by extracellular barium (By similarity). Subunit [...]
kmeans	1	Red	25	NPPB	9606.ENSP00000365651	Natriuretic peptides B; Cardiac hormone which may function as a paracrine antifibrotic factor in the heart. Also plays a key role in cardiovascular homeostasis through natriuresis, diuresis, vasorelaxation, and inhibition of renin and aldosterone secretion. Specifically binds and stimulates the cGMP production of the NPR1 receptor. Binds the clearance receptor NPR3;

						Belongs to the natriuretic peptide family
kmeans	1	Red	25	PIK3CA	9606.ENSP00000263967	Phosphatidylinositol 4,5-bisphosphate 3-kinase catalytic subunit alpha isoform; Phosphoinositide-3-kinase (PI3K) that phosphorylates PtdIns (Phosphatidylinositol), PtdIns4P (Phosphatidylinositol 4-phosphate) and PtdIns(4,5)P2 (Phosphatidylinositol 4,5-bisphosphate) to generate phosphatidylinositol 3,4,5-trisphosphate (PIP3). PIP3 plays a key role by recruiting PH domain-containing proteins to the membrane, including AKT1 and PDPK1, activating signaling cascades involved in cell growth, survival, proliferation, motility and morphology. Participates in cellular signaling in response to [...]
kmeans	1	Red	25	PLG	9606.ENSP00000308938	Plasminogen; Plasmin dissolves the fibrin of blood clots and acts as a proteolytic factor in a variety of other processes including embryonic development, tissue remodeling, tumor invasion, and inflammation. In ovulation, weakens the walls of the Graafian follicle. It activates the urokinase-type plasminogen activator, collagenases and

						several complement zymogens, such as C1 and C5. Cleavage of fibronectin and laminin leads to cell detachment and apoptosis. Also cleaves fibrin, thrombospondin and von Willebrand factor. Its role in tissue remodeling and tumor invasion may be modulated b [...]
kmeans	1	Red	25	PPARG	9606.ENSP00000287820	Peroxisome proliferator-activated receptor gamma; Nuclear receptor that binds peroxisome proliferators such as hypolipidemic drugs and fatty acids. Once activated by a ligand, the nuclear receptor binds to DNA specific PPAR response elements (PPRE) and modulates the transcription of its target genes, such as acyl-CoA oxidase. It therefore controls the peroxisomal beta-oxidation pathway of fatty acids. Key regulator of adipocyte differentiation and glucose homeostasis. ARF6 acts as a key regulator of the tissue-specific adipocyte P2 (aP2) enhancer. Acts as a critical regulator of gut ho [...]
kmeans	2	Green	24	ABCC9	9606.ENSP00000261200	ATP-binding cassette sub-family C member 9; Subunit of ATP-sensitive potassium channels (KATP). Can form cardiac and smooth muscle-type KATP channels with

						KCNJ11. KCNJ11 forms the channel pore while ABCC9 is required for activation and regulation; Belongs to the ABC transporter superfamily. ABCC family. Conjugate transporter (TC 3.A.1.208) subfamily
kmeans	2	Green	24	ACTN2	9606.ENSP00000443495	Alpha-actinin-2; F-actin cross-linking protein which is thought to anchor actin to a variety of intracellular structures. This is a bundling protein; Actinins
kmeans	2	Green	24	CACNA1S	9606.ENSP00000355192	Voltage-dependent L-type calcium channel subunit alpha-1S; Pore-forming, alpha-1S subunit of the voltage-gated calcium channel that gives rise to L-type calcium currents in skeletal muscle. Calcium channels containing the alpha-1S subunit play an important role in excitation-contraction coupling in skeletal muscle via their interaction with RYR1, which triggers Ca(2+) release from the sarcplasmic reticulum and ultimately results in muscle contraction. Long-lasting (L-type) calcium channels belong to the 'high-voltage activated' (HVA) group
kmeans	2	Green	24	CACNA2D1	9606.ENSP00000349320	Voltage-dependent calcium channel subunit alpha-2/delta-1; The alpha-2/delta subunit of voltage-dependent calcium channels regulates calcium current density and

						activation/inactivation kinetics of the calcium channel. Plays an important role in excitation-contraction coupling (By similarity)
kmeans	2	Green	24	CALM1	9606.ENSP00000349467	Calmodulin-1; Calmodulin mediates the control of a large number of enzymes, ion channels, aquaporins and other proteins through calcium-binding. Among the enzymes to be stimulated by the calmodulin-calcium complex are a number of protein kinases and phosphatases. Together with CCP110 and centrin, is involved in a genetic pathway that regulates the centrosome cycle and progression through cytokinesis. Mediates calcium-dependent inactivation of CACNA1C. Positively regulates calcium-activated potassium channel activity of KCNN2
kmeans	2	Green	24	KCNH2	9606.ENSP00000262186	Potassium voltage-gated channel subfamily H member 2; Pore-forming (alpha) subunit of voltage-gated inwardly rectifying potassium channel. Channel properties are modulated by cAMP and subunit assembly. Mediates the rapidly activating component of the delayed rectifying potassium current in heart (IKr)

kmeans	2	Green	24	KCNJ8	9606.ENSP00000240662	ATP-sensitive inward rectifier potassium channel 8; This potassium channel is controlled by G proteins. Inward rectifier potassium channels are characterized by a greater tendency to allow potassium to flow into the cell rather than out of it. Their voltage dependence is regulated by the concentration of extracellular potassium; as external potassium is raised, the voltage range of the channel opening shifts to more positive voltages. The inward rectification is mainly due to the blockage of outward current by internal magnesium. Can be blocked by external barium (By similarity); Belon [...]
kmeans	2	Green	24	RPL11	9606.ENSP00000363676	60S ribosomal protein L11; Component of the ribosome, a large ribonucleoprotein complex responsible for the synthesis of proteins in the cell. The small ribosomal subunit (SSU) binds messenger RNAs (mRNAs) and translates the encoded message by selecting cognate aminoacyl- transfer RNA (tRNA) molecules. The large subunit (LSU) contains the ribosomal catalytic site termed the peptidyl transferase center (PTC), which catalyzes the formation of peptide bonds, thereby polymerizing the amino acids delivered by

						tRNAs into a polypeptide chain. The nascent polypeptides leave the ribosome throug [...]
kmeans	2	Green	24	RPL15	9606.ENSP00000309334	Ribosomal protein L15
kmeans	2	Green	24	RRM2B	9606.ENSP00000251810	Ribonucleoside-diphosphate reductase subunit M2 B; Plays a pivotal role in cell survival by repairing damaged DNA in a p53/TP53-dependent manner. Supplies deoxyribonucleotides for DNA repair in cells arrested at G1 or G2. Contains an iron-tyrosyl free radical center required for catalysis. Forms an active ribonucleotide reductase (RNR) complex with RRM1 which is expressed both in resting and proliferating cells in response to DNA damage
kmeans	2	Green	24	RYR1	9606.ENSP00000352608	Ryanodine receptor 1; Calcium channel that mediates the release of Ca(2+) from the sarcoplasmic reticulum into the cytoplasm and thereby plays a key role in triggering muscle contraction following depolarization of T-tubules. Repeated very high-level exercise increases the open probability of the channel and leads to Ca(2+) leaking into the cytoplasm. Can also mediate the release of Ca(2+) from intracellular stores in neurons, and may thereby promote prolonged

						Ca(2+) signaling in the brain. Required for normal embryonic development of muscle fibers and skeletal muscle. Required for nor [...]
kmeans	2	Green	24	SCN10A	9606.ENSP00000390600	Sodium channel protein type 10 subunit alpha; Tetrodotoxin-resistant channel that mediates the voltage-dependent sodium ion permeability of excitable membranes. Assuming opened or closed conformations in response to the voltage difference across the membrane, the protein forms a sodium-selective channel through which sodium ions may pass in accordance with their electrochemical gradient. Plays a role in neuropathic pain mechanisms; Sodium voltage-gated channel alpha subunits
kmeans	2	Green	24	SCN11A	9606.ENSP00000307599	Sodium channel protein type 11 subunit alpha; This protein mediates the voltage-dependent sodium ion permeability of excitable membranes. Assuming opened or closed conformations in response to the voltage difference across the membrane, the protein forms a sodium-selective channel through which sodium ions may pass in accordance with their electrochemical gradient. It is a tetrodotoxin-resistant

						sodium channel isoform. Also involved, with the contribution of the receptor tyrosine kinase NTRK2, in rapid BDNF-evoked neuronal depolarization; Sodium voltage-gated channel alpha subunits
kmeans	2	Green	24	SCN1B	9606.ENSP00000396915	Sodium channel subunit beta-1; Crucial in the assembly, expression, and functional modulation of the heterotrimeric complex of the sodium channel. The subunit beta-1 can modulate multiple alpha subunit isoforms from brain, skeletal muscle, and heart. Its association with NFASC may target the sodium channels to the nodes of Ranvier of developing axons and retain these channels at the nodes in mature myelinated axons; Immunoglobulin like domain containing
kmeans	2	Green	24	SCN2B	9606.ENSP00000278947	Sodium channel subunit beta-2; Crucial in the assembly, expression, and functional modulation of the heterotrimeric complex of the sodium channel. The subunit beta-2 causes an increase in the plasma membrane surface area and in its folding into microvilli. Interacts with TNR may play a crucial role in clustering and regulation of activity of sodium channels at nodes of Ranvier (By similarity); Belongs to the sodium

						channel auxiliary subunit SCN2B (TC 8.A.17) family
kmeans	2	Green	24	SCN3B	9606.ENSP00000376523	Sodium channel subunit beta-3; Modulates channel gating kinetics. Causes unique persistent sodium currents. Inactivates the sodium channel opening more slowly than the subunit beta-1. Its association with NFASC may target the sodium channels to the nodes of Ranvier of developing axons and retain these channels at the nodes in mature myelinated axons (By similarity); Belongs to the sodium channel auxiliary subunit SCN3B (TC 8.A.17) family
kmeans	2	Green	24	SCN4A	9606.ENSP00000396320	Sodium channel protein type 4 subunit alpha; This protein mediates the voltage-dependent sodium ion permeability of excitable membranes. Assuming opened or closed conformations in response to the voltage difference across the membrane, the protein forms a sodium-selective channel through which Na(+) ions may pass in accordance with their electrochemical gradient. This sodium channel may be present in both denervated and innervated skeletal muscle; Belongs to the sodium channel (TC 1.A.1.10) family. Nav1.4/SCN4A subfamily

kmeans	2	Green	24	SCN4B	9606.ENSP00000322460	Sodium channel subunit beta-4; Modulates channel gating kinetics. Causes negative shifts in the voltage dependence of activation of certain alpha sodium channels, but does not affect the voltage dependence of inactivation. Modulates the suceptibility of the sodium channel to inhibition by toxic peptides from spider, scorpion, wasp and sea anemone venom; Sodium voltage-gated channel beta subunits
kmeans	2	Green	24	SCN5A	9606.ENSP00000410257	Sodium channel protein type 5 subunit alpha; This protein mediates the voltage-dependent sodium ion permeability of excitable membranes. Assuming opened or closed conformations in response to the voltage difference across the membrane, the protein forms a sodium-selective channel through which Na(+) ions may pass in accordance with their electrochemical gradient. It is a tetrodotoxin-resistant Na(+) channel isoform. This channel is responsible for the initial upstroke of the action potential. Channel inactivation is regulated by intracellular calcium levels; Sodium voltage-gated channe [...]
kmeans	2	Green	24	SCNN1A	9606.ENSP00000353292	Amiloride-sensitive sodium channel subunit alpha; Sodium permeable

						non-voltage-sensitive ion channel inhibited by the diuretic amiloride. Mediates the electrodiffusion of the luminal sodium (and water, which follows osmotically) through the apical membrane of epithelial cells. Plays an essential role in electrolyte and blood pressure homeostasis, but also in airway surface liquid homeostasis, which is important for proper clearance of mucus. Controls the reabsorption of sodium in kidney, colon, lung and eccrine sweat glands. Also plays a role in taste perception
kmeans	2	Green	24	SCNN1B	9606.ENSP00000345751	Amiloride-sensitive sodium channel subunit beta; Sodium permeable non-voltage-sensitive ion channel inhibited by the diuretic amiloride. Mediates the electrodiffusion of the luminal sodium (and water, which follows osmotically) through the apical membrane of epithelial cells. Plays an essential role in electrolyte and blood pressure homeostasis, but also in airway surface liquid homeostasis, which is important for proper clearance of mucus. Controls the reabsorption of sodium in kidney, colon, lung and sweat glands. Also plays a role in taste perception; Belongs to the amiloride-sensit [...]

kmeans	2	Green	24	SCNN1G	9606.ENSP00000300061	Amiloride-sensitive sodium channel subunit gamma; Sodium permeable non-voltage-sensitive ion channel inhibited by the diuretic amiloride. Mediates the electrodiffusion of the luminal sodium (and water, which follows osmotically) through the apical membrane of epithelial cells. Plays an essential role in electrolyte and blood pressure homeostasis, but also in airway surface liquid homeostasis, which is important for proper clearance of mucus. Controls the reabsorption of sodium in kidney, colon, lung and sweat glands. Also plays a role in taste perception
kmeans	2	Green	24	SLC19A2	9606.ENSP00000236137	Thiamine transporter 1; High-affinity transporter for the intake of thiamine; Solute carriers
kmeans	2	Green	24	SLC25A4	9606.ENSP00000281456	ADP/ATP translocase 1; Involved in mitochondrial ADP/ATP transport. Catalyzes the exchange of cytoplasmic ADP with mitochondrial ATP across the mitochondrial inner membrane; Belongs to the mitochondrial carrier (TC 2.A.29) family
kmeans	3	Blue	9	COX7B	9606.ENSP00000417656	Cytochrome c oxidase subunit 7B, mitochondrial; This protein is one of the nuclear-coded polypeptide chains of cytochrome c oxidase, the terminal oxidase in mitochondrial

						electron transport. Plays a role in proper central nervous system (CNS) development in vertebrates
kmeans	3	Blue	9	MT-CO1	9606.ENSP00000354499	Cytochrome c oxidase subunit 1; Cytochrome c oxidase is the component of the respiratory chain that catalyzes the reduction of oxygen to water. Subunits 1- 3 form the functional core of the enzyme complex. CO I is the catalytic subunit of the enzyme. Electrons originating in cytochrome c are transferred via the copper A center of subunit 2 and heme A of subunit 1 to the bimetallic center formed by heme A3 and copper B
kmeans	3	Blue	9	MT-CO2	9606.ENSP00000354876	Cytochrome c oxidase subunit 2; Cytochrome c oxidase is the component of the respiratory chain that catalyzes the reduction of oxygen to water. Subunits 1- 3 form the functional core of the enzyme complex. Subunit 2 transfers the electrons from cytochrome c via its binuclear copper A center to the bimetallic center of the catalytic subunit 1
kmeans	3	Blue	9	MT-CO3	9606.ENSP00000354982	Cytochrome c oxidase subunit 3; Subunits I, II and III form the functional core of the enzyme complex; Mitochondrial complex IV: cytochrome c oxidase subunits
kmeans	3	Blue	9	PTGS1	9606.ENSP00000354612	Prostaglandin G/H synthase 1; Converts arachidonate to

						prostaglandin H2 (PGH2), a committed step in prostanoid synthesis. Involved in the constitutive production of prostanoids in particular in the stomach and platelets. In gastric epithelial cells, it is a key step in the generation of prostaglandins, such as prostaglandin E2 (PGE2), which plays an important role in cytoprotection. In platelets, it is involved in the generation of thromboxane A2 (TXA2), which promotes platelet activation and aggregation, vasoconstriction and proliferation of vascular smooth muscle cells; Belongs to th [...]
kmeans	3	Blue	9	SDHA	9606.ENSP00000264932	Succinate dehydrogenase [ubiquinone] flavoprotein subunit, mitochondrial; Flavoprotein (FP) subunit of succinate dehydrogenase (SDH) that is involved in complex II of the mitochondrial electron transport chain and is responsible for transferring electrons from succinate to ubiquinone (coenzyme Q). Can act as a tumor suppressor; Belongs to the FAD-dependent oxidoreductase 2 family. FRD/SDH subfamily
kmeans	3	Blue	9	SDHB	9606.ENSP00000364649	Succinate dehydrogenase [ubiquinone] iron-sulfur subunit, mitochondrial; Iron-sulfur protein (IP) subunit of succinate

						dehydrogenase (SDH) that is involved in complex II of the mitochondrial electron transport chain and is responsible for transferring electrons from succinate to ubiquinone (coenzyme Q)
kmeans	3	Blue	9	SDHC	9606.ENSP00000356953	Succinate dehydrogenase cytochrome b560 subunit, mitochondrial; Membrane-anchoring subunit of succinate dehydrogenase (SDH) that is involved in complex II of the mitochondrial electron transport chain and is responsible for transferring electrons from succinate to ubiquinone (coenzyme Q); Belongs to the cytochrome b560 family
kmeans	3	Blue	9	TNF	9606.ENSP00000398698	Tumor necrosis factor; Cytokine that binds to TNFRSF1A/TNFR1 and TNFRSF1B/TNFBR. It is mainly secreted by macrophages and can induce cell death of certain tumor cell lines. It is potent pyrogen causing fever by direct action or by stimulation of interleukin-1 secretion and is implicated in the induction of cachexia, Under certain conditions it can stimulate cell proliferation and induce cell differentiation. Impairs regulatory T-cells (Treg) function in individuals with rheumatoid arthritis via FOXP3 dephosphorylation. Upregulates the expression of protein phosphatase 1

	(PP1), which de [...]

Table S8 PRP acts directly on disease targets and topological parameters (Degree>10)

NO	Gene name	ENTREZID	Degree
1	INS	3630	33
2	CACNA1C	775	25
3	SCN5A	6331	25
4	SCN1B	6324	25
5	SCN10A	6336	24
6	SCN3B	55800	22
7	CACNA2D1	781	22
8	SCN3B	55800	22
9	SCN4B	6330	21
10	ADORA1	134	21
11	ADORA2B	136	20
12	CACNB2	154	19
13	SCN4A	6329	17
14	ABCC9	10060	17
15	TNF	7124	16
16	CACNA1B	774	16
17	KCNJ8	3764	16
18	KCNJ11	3767	15
19	CACNA1S	779	13
20	SDHC	6391	13
21	ADRB2	154	13
22	AGTR1	185	13
23	PPARG	5468	13
24	SCNN1A	6337	12
25	RYR1	6261	12
26	SCN11A	11280	11
27	CALM1	801	11
28	ADRA1B	147	10
29	ADRA1A	148	10

Table S9 Gene Ontology annotations of biological processes (BP); cellular components (CC) and molecular functions (MF)

ONTOLOGY	ID	Description	GeneRatio	BgRatio	pvalue	p.adjust	qvalue	geneID	Count
BP	GO:1903522	regulation of blood circulation	25/57	297/18670	2.49E-30	5.12E-27	3.38E-27	148/150/153/147/152/185/151/6327/55800/6331	25

								/6336/6330/6324/801/134/10060/775/776/3757/783/3767/779/774/6261/781	
BP	GO:0006936	muscle contraction	26/57	360/18670	7.46E-30	7.66E-27	5.06E-27	3764/148/154/150/147/152/151/88/6327/55800/6331/6336/6329/6330/6324/801/134/136/775/776/3757/783/779/6261/5290/781	26
BP	GO:0060047	heart contraction	23/57	280/18670	1.51E-27	1.03E-24	6.83E-25	3764/148/153/147/6327/55800/6331/6336/6330/6324/801/134/10060/775/776/3757/783/3767/779/774/6261/5290/781	23
BP	GO:0003015	heart process	23/57	290/18670	3.44E-27	1.77E-24	1.17E-24	3764/148/153/147/6327/55800/6331/6336/6330/6324/801/134/10060/775/776/3757/783/3767/779/774/6261/5290/781	23
BP	GO:0003012	muscle system process	26/57	465/18670	6.03E-27	2.48E-24	1.64E-24	3764/148/154/150/147/152/151/88/6327/55800/6331/6336/6329/6330/6324/801/134/136/775/776/3757/783/779/6261/5290/781	26
BP	GO:0008016	regulation of heart contraction	21/57	251/18670	2.98E-25	1.02E-22	6.73E-23	148/153/147/6327/55800/6331/6336/6330/6324/801/134/10060/775/776/3757/783/3767/779/774/6261/781	21

BP	GO:0086010	membrane depolarization during action potential	13/57	37/18670	1.55E-24	4.53E-22	2.99E-22	11280/6327/55800/6331/6336/6329/6330/6324/775/776/3757/783/781	13
BP	GO:0060048	cardiac muscle contraction	17/57	137/18670	2.03E-23	5.21E-21	3.44E-21	3764/148/147/6327/55800/6331/6336/6330/6324/801/134/775/776/3757/783/5290/781	17
BP	GO:0006941	striated muscle contraction	18/57	174/18670	2.60E-23	5.92E-21	3.91E-21	3764/148/147/6327/55800/6331/6336/6329/6330/6324/801/134/775/776/3757/783/5290/781	18
BP	GO:0035637	multicellular organismal signaling	18/57	204/18670	4.92E-22	1.01E-19	6.67E-20	11280/6327/55800/6331/6336/6329/6330/6324/801/10060/775/776/3757/783/3767/779/6261/781	18
BP	GO:0061337	cardiac conduction	16/57	146/18670	3.71E-21	6.92E-19	4.57E-19	6327/55800/6331/6336/6330/6324/801/10060/775/776/3757/783/3767/779/6261/781	16
BP	GO:0051899	membrane depolarization	14/57	91/18670	9.75E-21	1.67E-18	1.10E-18	11280/6327/55800/6331/6336/6329/6330/6324/775/776/3757/783/774/781	14
BP	GO:0042391	regulation of membrane potential	21/57	434/18670	3.02E-20	4.76E-18	3.15E-18	3764/148/154/153/88/11280/6327/55800/6331/6336/6329/6330/6324/134/775/776/3757/783/3767/774/781	21
BP	GO:0002027	regulation of heart rate	14/57	101/186	4.58E-20	6.43E-18	4.25E-18	148/153/6327/55800/6331/6336	14

				70				/6330/6324/801/775/776/3757/783/781	
BP	GO:0001508	action potential	15/57	133/18670	4.70E-20	6.43E-18	4.25E-18	3764/148/11280/6327/55800/6331/6336/6329/6330/6324/775/776/3757/783/781	15
BP	GO:0034765	regulation of ion transmembrane transport	21/57	483/18670	2.73E-19	3.51E-17	2.32E-17	3764/154/150/88/11280/6327/55800/6331/6336/6329/6330/6324/801/775/776/3757/783/3767/779/774/781	21
BP	GO:0086003	cardiac muscle cell contraction	12/57	69/18670	1.47E-18	1.78E-16	1.17E-16	3764/6327/55800/6331/6330/6324/134/775/776/3757/783/781	12
BP	GO:0086001	cardiac muscle cell action potential	12/57	74/18670	3.62E-18	4.13E-16	2.73E-16	3764/6327/55800/6331/6336/6330/6324/775/776/3757/783/781	12
BP	GO:0086012	membrane depolarization during cardiac muscle cell action potential	9/57	23/18670	9.39E-18	1.01E-15	6.70E-16	6327/55800/6331/6330/6324/775/776/783/781	9
BP	GO:0086002	cardiac muscle cell action potential involved in contraction	11/57	56/18670	1.03E-17	1.06E-15	7.01E-16	3764/6327/55800/6331/6330/6324/775/776/3757/783/781	11
BP	GO:0086091	regulation of heart rate by cardiac conduction	10/57	38/18670	1.35E-17	1.32E-15	8.74E-16	6327/55800/6331/6330/6324/775/776/3757/783/781	10
BP	GO:0070252	actin-mediated cell contraction	13/57	116/18670	2.01E-17	1.87E-15	1.24E-15	3764/88/6327/55800/6331/6330/6324/134/775/776/3757/783/781	13

BP	GO:0030048	actin filament-based movement	13/57	138/18670	2.05E-16	1.83E-14	1.21E-14	3764/88/6327/55800/6331/6330/6324/134/775/776/3757/783/781	13
BP	GO:0086019	cell-cell signaling involved in cardiac conduction	9/57	32/18670	3.16E-16	2.50E-14	1.65E-14	55800/6331/6336/6330/6324/775/776/783/781	9
BP	GO:0035296	regulation of tube diameter	13/57	143/18670	3.29E-16	2.50E-14	1.65E-14	4879/148/154/150/153/147/152/185/151/3630/134/136/1401	13
BP	GO:0050880	regulation of blood vessel size	13/57	143/18670	3.29E-16	2.50E-14	1.65E-14	4879/148/154/150/153/147/152/185/151/3630/134/136/1401	13
BP	GO:0097746	regulation of blood vessel diameter	13/57	143/18670	3.29E-16	2.50E-14	1.65E-14	4879/148/154/150/153/147/152/185/151/3630/134/136/1401	13
BP	GO:0035150	regulation of tube size	13/57	144/18670	3.61E-16	2.64E-14	1.75E-14	4879/148/154/150/153/147/152/185/151/3630/134/136/1401	13
BP	GO:0086065	cell communication involved in cardiac conduction	10/57	56/18670	9.78E-16	6.92E-14	4.57E-14	55800/6331/6336/6330/6324/801/775/776/783/781	10
BP	GO:0006937	regulation of muscle contraction	13/57	171/18670	3.47E-15	2.38E-13	1.57E-13	148/154/150/147/152/151/6331/6336/6329/801/134/136/775	13
BP	GO:0003018	vascular process in circulatory system	13/57	173/18670	4.04E-15	2.68E-13	1.77E-13	4879/148/154/150/153/147/152/185/151/3630/134/136/1401	13
BP	GO:0010959	regulation of metal ion transport	16/57	394/18670	3.01E-14	1.93E-12	1.27E-12	154/150/88/6327/55800/6331/6330/6324/801/134/775/776/3757/783/774/781	16

BP	GO:0071880	adenylate cyclase-activating adrenergic receptor signaling pathway	8/57	31/18670	3.38E-14	2.10E-12	1.39E-12	148/154/150/153/146/147/152/151	8
BP	GO:0043270	positive regulation of ion transport	14/57	275/18670	7.10E-14	4.17E-12	2.75E-12	154/150/88/55800/6331/6330/6324/801/134/776/3757/783/3767/781	14
BP	GO:1904062	regulation of cation transmembrane transport	15/57	342/18670	7.11E-14	4.17E-12	2.75E-12	154/150/88/6327/55800/6331/6330/6324/801/775/776/3757/783/3767/781	15
BP	GO:0071875	adrenergic receptor signaling pathway	8/57	34/18670	7.72E-14	4.40E-12	2.91E-12	148/154/150/153/146/147/152/151	8
BP	GO:0032409	regulation of transporter activity	14/57	283/18670	1.05E-13	5.84E-12	3.86E-12	5468/154/150/3630/88/6327/55800/6330/6324/801/776/783/3767/781	14
BP	GO:0023061	signal release	16/57	462/18670	3.49E-13	1.88E-11	1.24E-11	148/150/152/185/151/3630/134/136/6833/291/775/776/783/3767/774/7124	16
BP	GO:0035725	sodium ion transmembrane transport	11/57	140/18670	3.89E-13	2.05E-11	1.35E-11	6337/6340/6338/11280/6327/55800/6331/6336/6329/6330/6324	11
BP	GO:0090257	regulation of muscle system process	13/57	259/18670	7.42E-13	3.81E-11	2.52E-11	148/154/150/147/152/151/6331/6336/6329/801/134/136/775	13
BP	GO:0022898	regulation of transmembrane transporter activity	13/57	268/18670	1.15E-12	5.74E-11	3.79E-11	154/150/3630/88/6327/55800/6330/6324/801/776/783/3767/781	13
BP	GO:00068	sodium ion	12/57	218/	2.11	1.03	6.82E-1	6337/6340/154/	12

	14	transport		18670	E-12	E-10	1	6338/11280/6327/55800/6331/6336/6329/6330/6324	
BP	GO:0019932	second-messenger-mediated signaling	15/57	439/18670	2.62E-12	1.25E-10	8.26E-11	4879/148/154/150/153/146/147/152/185/151/3630/801/136/775/7124	15
BP	GO:0097756	negative regulation of blood vessel diameter	9/57	84/18670	3.67E-12	1.71E-10	1.13E-10	148/150/147/152/185/151/3630/134/1401	9
BP	GO:0007187	G protein-coupled receptor signaling pathway, coupled to cyclic nucleotide second messenger	12/57	256/18670	1.40E-11	6.38E-10	4.21E-10	148/154/150/153/146/147/152/151/3360/134/136/776	12
BP	GO:0032412	regulation of ion transmembrane transporter activity	12/57	260/18670	1.68E-11	7.48E-10	4.94E-10	154/150/88/6327/55800/6330/6324/801/776/783/3767/781	12
BP	GO:0006940	regulation of smooth muscle contraction	8/57	65/18670	2.00E-11	8.72E-10	5.76E-10	148/154/150/147/152/151/134/136	8
BP	GO:0046883	regulation of hormone secretion	12/57	266/18670	2.19E-11	9.35E-10	6.17E-10	150/152/185/151/3630/134/6833/291/775/776/3767/7124	12
BP	GO:0099622	cardiac muscle cell membrane repolarization	7/57	42/18670	4.19E-11	1.74E-09	1.15E-09	3764/6331/6330/6324/776/3757/781	7
BP	GO:0019935	cyclic-nucleotide-mediated signaling	11/57	215/18670	4.24E-11	1.74E-09	1.15E-09	4879/148/154/150/153/146/147/152/151/801/136	11
BP	GO:0060371	regulation of atrial cardiac	5/57	10/1867	5.52E-11	2.22E-09	1.47E-09	6327/55800/6331/6336/6324	5

		muscle cell membrane depolarization		0					
BP	GO:0007188	adenylate cyclase-modulating G protein-coupled receptor signaling pathway	11/57	221/18670	5.70E-11	2.25E-09	1.49E-09	148/154/150/153/146/147/152/151/134/136/776	11
BP	GO:0086016	AV node cell action potential	5/57	11/18670	1.01E-10	3.84E-09	2.54E-09	6331/6336/6330/775/783	5
BP	GO:0086027	AV node cell to bundle of His cell signaling	5/57	11/18670	1.01E-10	3.84E-09	2.54E-09	6331/6336/6330/775/783	5
BP	GO:0046879	hormone secretion	12/57	312/18670	1.39E-10	5.18E-09	3.42E-09	150/152/185/151/3630/134/6833/291/775/776/3767/7124	12
BP	GO:0086009	membrane repolarization	7/57	50/18670	1.52E-10	5.58E-09	3.69E-09	3764/6331/6330/6324/776/3757/781	7
BP	GO:0086067	AV node cell to bundle of His cell communication	5/57	12/18670	1.73E-10	6.22E-09	4.11E-09	6331/6336/6330/775/783	5
BP	GO:0008217	regulation of blood pressure	10/57	182/18670	1.76E-10	6.22E-09	4.11E-09	4879/5468/148/154/153/147/185/151/134/774	10
BP	GO:0009914	hormone transport	12/57	322/18670	1.99E-10	6.94E-09	4.58E-09	150/152/185/151/3630/134/6833/291/775/776/3767/7124	12
BP	GO:0019933	cAMP-mediated signaling	10/57	187/18670	2.29E-10	7.84E-09	5.18E-09	148/154/150/153/146/147/152/151/801/136	10
BP	GO:0007189	adenylate cyclase-activating G protein-coupled receptor	9/57	139/18670	3.60E-10	1.21E-08	7.99E-09	148/154/150/153/146/147/152/151/136	9

		signaling pathway							
BP	GO:0099625	ventricular cardiac muscle cell membrane repolarization	6/57	31/18670	4.29E-10	1.42E-08	9.37E-09	3764/6331/6330/6324/3757/781	6
BP	GO:0099623	regulation of cardiac muscle cell membrane repolarization	6/57	32/18670	5.26E-10	1.70E-08	1.12E-08	6331/6330/6324/776/3757/781	6
BP	GO:0006942	regulation of striated muscle contraction	8/57	97/18670	5.30E-10	1.70E-08	1.12E-08	148/147/6331/6336/6329/801/134/775	8
BP	GO:0007588	excretion	7/57	63/18670	8.18E-10	2.59E-08	1.71E-08	4879/148/6340/185/6338/134/136	7
BP	GO:0006939	smooth muscle contraction	8/57	110/18670	1.46E-09	4.54E-08	3.00E-08	148/154/150/147/152/151/134/136	8
BP	GO:0060306	regulation of membrane repolarization	6/57	39/18670	1.86E-09	5.71E-08	3.77E-08	6331/6330/6324/776/3757/781	6
BP	GO:0003085	negative regulation of systemic arterial blood pressure	5/57	19/18670	2.49E-09	7.53E-08	4.97E-08	4879/148/154/153/134	5
BP	GO:0055117	regulation of cardiac muscle contraction	7/57	82/18670	5.38E-09	1.60E-07	1.06E-07	148/147/6331/6336/801/134/775	7
BP	GO:0009060	aerobic respiration	7/57	87/18670	8.17E-09	2.40E-07	1.58E-07	1349/4512/4513/4514/6390/6391/6389	7
BP	GO:0055078	sodium ion homeostasis	6/57	52/18670	1.13E-08	3.26E-07	2.15E-07	4879/6337/6340/185/6338/134	6
BP	GO:0090276	regulation of peptide hormone secretion	9/57	208/18670	1.26E-08	3.59E-07	2.37E-07	150/152/3630/6833/291/775/776/3767/7124	9
BP	GO:0003073	regulation of systemic arterial blood pressure	7/57	93/18670	1.31E-08	3.67E-07	2.43E-07	4879/148/154/153/147/185/134	7

BP	GO:0015980	energy derivation by oxidation of organic compounds	10/57	285/18670	1.35E-08	3.73E-07	2.47E-07	1349/4512/4513/4514/3630/801/5290/6390/6391/6389	10
BP	GO:0060307	regulation of ventricular cardiac muscle cell membrane repolarization	5/57	27/18670	1.70E-08	4.65E-07	3.07E-07	6331/6330/6324/3757/781	5
BP	GO:0019229	regulation of vasoconstriction	6/57	58/18670	2.21E-08	5.98E-07	3.95E-07	148/150/147/152/185/151	6
BP	GO:0006813	potassium ion transport	9/57	240/18670	4.34E-08	1.16E-06	7.64E-07	3764/150/88/134/10060/6833/776/3757/3767	9
BP	GO:0050796	regulation of insulin secretion	8/57	176/18670	5.93E-08	1.56E-06	1.03E-06	150/152/6833/291/775/776/3767/7124	8
BP	GO:0030072	peptide hormone secretion	9/57	250/18670	6.16E-08	1.60E-06	1.06E-06	150/152/3630/6833/291/775/776/3767/7124	9
BP	GO:0022904	respiratory electron transport chain	7/57	117/18670	6.46E-08	1.66E-06	1.10E-06	1349/4512/4513/4514/6390/6391/6389	7
BP	GO:2001259	positive regulation of cation channel activity	6/57	70/18670	6.97E-08	1.77E-06	1.17E-06	154/88/801/783/3767/781	6
BP	GO:0045777	positive regulation of blood pressure	5/57	37/18670	8.97E-08	2.22E-06	1.46E-06	148/153/147/151/134	5
BP	GO:2000273	positive regulation of signaling receptor activity	5/57	37/18670	8.97E-08	2.22E-06	1.46E-06	154/150/152/151/134	5
BP	GO:0086005	ventricular cardiac muscle cell action potential	5/57	38/18670	1.03E-07	2.52E-06	1.66E-06	3764/55800/6331/775/3757	5
BP	GO:0051480	regulation of cytosolic calcium ion	10/57	357/18670	1.12E-07	2.70E-06	1.79E-06	148/147/185/801/134/775/776/783/6261/781	10

		concentration							
BP	GO:0042310	vasoconstriction	6/57	76/18670	1.15E-07	2.73E-06	1.81E-06	148/150/147/152/185/151	6
BP	GO:0045333	cellular respiration	8/57	193/18670	1.21E-07	2.85E-06	1.88E-06	1349/4512/4513/4514/5290/6390/6391/6389	8
BP	GO:0045986	negative regulation of smooth muscle contraction	4/57	16/18670	1.38E-07	3.22E-06	2.13E-06	154/152/134/136	4
BP	GO:0070509	calcium ion import	6/57	80/18670	1.56E-07	3.60E-06	2.38E-06	775/776/783/779/774/781	6
BP	GO:0034764	positive regulation of transmembrane transport	8/57	204/18670	1.85E-07	4.22E-06	2.79E-06	154/3630/88/801/3757/783/3767/781	8
BP	GO:0030073	insulin secretion	8/57	207/18670	2.07E-07	4.67E-06	3.08E-06	150/152/6833/291/775/776/3767/7124	8
BP	GO:0002028	regulation of sodium ion transport	6/57	85/18670	2.24E-07	5.01E-06	3.31E-06	154/6327/55800/6331/6330/6324	6
BP	GO:1904064	positive regulation of cation transmembrane transport	7/57	144/18670	2.69E-07	5.93E-06	3.92E-06	154/88/801/3757/783/3767/781	7
BP	GO:0002791	regulation of peptide secretion	11/57	500/18670	2.76E-07	5.97E-06	3.94E-06	150/152/3630/134/6833/291/775/776/3767/1401/7124	11
BP	GO:0045776	negative regulation of blood pressure	5/57	46/18670	2.76E-07	5.97E-06	3.94E-06	4879/148/154/153/134	5
BP	GO:0086014	atrial cardiac muscle cell action potential	4/57	20/18670	3.65E-07	7.64E-06	5.04E-06	55800/6331/775/783	4
BP	GO:0086026	atrial cardiac muscle cell to AV node cell signaling	4/57	20/18670	3.65E-07	7.64E-06	5.04E-06	55800/6331/775/783	4

BP	GO:0086066	atrial cardiac muscle cell to AV node cell communication	4/57	20/18670	3.65E-07	7.64E-06	5.04E-06	55800/6331/775/783	4
BP	GO:0003044	regulation of systemic arterial blood pressure mediated by a chemical signal	5/57	49/18670	3.82E-07	7.83E-06	5.17E-06	148/154/153/147/185	5
BP	GO:0003254	regulation of membrane depolarization	5/57	49/18670	3.82E-07	7.83E-06	5.17E-06	6327/55800/6331/6336/6324	5
BP	GO:0055067	monovalent inorganic cation homeostasis	7/57	154/18670	4.24E-07	8.62E-06	5.69E-06	4879/6337/6340/185/6338/134/3757	7
BP	GO:0070588	calcium ion transmembrane transport	9/57	315/18670	4.37E-07	8.79E-06	5.80E-06	150/801/775/776/783/779/774/6261/781	9
BP	GO:0006123	mitochondrial electron transport, cytochrome c to oxygen	4/57	21/18670	4.49E-07	8.87E-06	5.86E-06	1349/4512/4513/4514	4
BP	GO:0019646	aerobic electron transport chain	4/57	21/18670	4.49E-07	8.87E-06	5.86E-06	1349/4512/4513/4514	4
BP	GO:0034767	positive regulation of ion transmembrane transport	7/57	156/18670	4.63E-07	9.05E-06	5.97E-06	154/88/801/3757/783/3767/781	7
BP	GO:0007204	positive regulation of cytosolic calcium ion concentration	9/57	319/18670	4.85E-07	9.40E-06	6.21E-06	148/147/185/801/775/776/783/6261/781	9
BP	GO:0042775	mitochondrial ATP synthesis coupled electron	6/57	97/18670	4.93E-07	9.46E-06	6.25E-06	1349/4512/4513/4514/6391/6389	6

		transport							
BP	GO:0042773	ATP synthesis coupled electron transport	6/57	98/18670	5.24E-07	9.96E-06	6.58E-06	1349/4512/4513/4514/6391/6389	6
BP	GO:0050995	negative regulation of lipid catabolic process	4/57	23/18670	6.62E-07	1.25E-05	8.23E-06	150/3630/134/7124	4
BP	GO:0006816	calcium ion transport	10/57	434/18670	6.76E-07	1.26E-05	8.33E-06	148/150/801/775/776/783/779/774/6261/781	10
BP	GO:0032147	activation of protein kinase activity	9/57	333/18670	6.95E-07	1.29E-05	8.49E-06	154/150/152/151/3630/134/136/7124/5290	9
BP	GO:0032414	positive regulation of ion transmembrane transporter activity	6/57	104/18670	7.45E-07	1.36E-05	9.01E-06	154/88/801/783/3767/781	6
BP	GO:0050806	positive regulation of synaptic transmission	7/57	168/18670	7.63E-07	1.39E-05	9.16E-06	148/154/3630/134/776/783/7124	7
BP	GO:0097755	positive regulation of blood vessel diameter	5/57	59/18670	9.79E-07	1.76E-05	1.16E-05	154/153/3630/134/136	5
BP	GO:0006874	cellular calcium ion homeostasis	10/57	458/18670	1.10E-06	1.97E-05	1.30E-05	148/147/185/801/134/775/776/783/6261/781	10
BP	GO:2001257	regulation of cation channel activity	7/57	178/18670	1.13E-06	1.99E-05	1.32E-05	154/88/6324/801/783/3767/781	7
BP	GO:0032411	positive regulation of transporter activity	6/57	112/18670	1.15E-06	2.02E-05	1.34E-05	154/88/801/783/3767/781	6
BP	GO:1902305	regulation of sodium ion transmembrane transport	5/57	62/18670	1.26E-06	2.19E-05	1.44E-05	6327/55800/6331/6330/6324	5
BP	GO:00459	negative	4/57	27/1	1.30	2.22	1.47E-0	154/152/134/13	4

	32	regulation of muscle contraction		8670	E-06	E-05	5	6	
BP	GO:0051953	negative regulation of amine transport	4/57	27/18670	1.30E-06	2.22E-05	1.47E-05	150/152/151/134	4
BP	GO:0055074	calcium ion homeostasis	10/57	471/18670	1.42E-06	2.40E-05	1.59E-05	148/147/185/801/134/775/776/783/6261/781	10
BP	GO:0050708	regulation of protein secretion	10/57	472/18670	1.44E-06	2.43E-05	1.60E-05	150/152/3630/6833/291/775/776/3767/1401/7124	10
BP	GO:0022900	electron transport chain	7/57	186/18670	1.51E-06	2.52E-05	1.66E-05	1349/4512/4513/4514/6390/6391/6389	7
BP	GO:0046888	negative regulation of hormone secretion	5/57	66/18670	1.72E-06	2.85E-05	1.88E-05	150/152/151/134/3767	5
BP	GO:0070838	divalent metal ion transport	10/57	483/18670	1.78E-06	2.92E-05	1.93E-05	148/150/801/775/776/783/779/774/6261/781	10
BP	GO:0072511	divalent inorganic cation transport	10/57	489/18670	1.98E-06	3.23E-05	2.14E-05	148/150/801/775/776/783/779/774/6261/781	10
BP	GO:0072503	cellular divalent inorganic cation homeostasis	10/57	493/18670	2.13E-06	3.45E-05	2.28E-05	148/147/185/801/134/775/776/783/6261/781	10
BP	GO:0042311	vasodilation	4/57	32/18670	2.63E-06	4.22E-05	2.79E-05	154/153/134/136	4
BP	GO:0019226	transmission of nerve impulse	5/57	72/18670	2.65E-06	4.22E-05	2.79E-05	11280/6331/6336/6329/6324	5
BP	GO:1903524	positive regulation of blood circulation	5/57	73/18670	2.84E-06	4.49E-05	2.96E-05	148/153/147/152/55800	5
BP	GO:00860	SA node cell to	3/57	10/1	3.19	5.00	3.30E-0	55800/6331/776	3

	70	atrial cardiac muscle cell communication		8670	E-06	E-05	5	
BP	GO:0010765	positive regulation of sodium ion transport	4/57	34/18670	3.38E-06	5.18E-05	3.42E-05	55800/6331/6330/6324	4
BP	GO:0019228	neuronal action potential	4/57	34/18670	3.38E-06	5.18E-05	3.42E-05	11280/6331/6336/6329	4
BP	GO:0032148	activation of protein kinase B activity	4/57	34/18670	3.38E-06	5.18E-05	3.42E-05	150/152/151/3630	4
BP	GO:1903531	negative regulation of secretion by cell	7/57	211/18670	3.49E-06	5.31E-05	3.51E-05	150/152/151/3630/134/3767/7124	7
BP	GO:0071804	cellular potassium ion transport	7/57	217/18670	4.20E-06	6.29E-05	4.16E-05	3764/88/10060/6833/776/3757/3767	7
BP	GO:0071805	potassium ion transmembrane transport	7/57	217/18670	4.20E-06	6.29E-05	4.16E-05	3764/88/10060/6833/776/3757/3767	7
BP	GO:0010742	macrophage derived foam cell differentiation	4/57	36/18670	4.28E-06	6.31E-05	4.17E-05	5468/185/1401/2033	4
BP	GO:0090077	foam cell differentiation	4/57	36/18670	4.28E-06	6.31E-05	4.17E-05	5468/185/1401/2033	4
BP	GO:0006119	oxidative phosphorylation	6/57	145/18670	5.18E-06	7.60E-05	5.02E-05	1349/4512/4513/4514/6391/6389	6
BP	GO:0098659	inorganic cation import across plasma membrane	5/57	83/18670	5.37E-06	7.76E-05	5.13E-05	3764/10060/3757/3767/781	5
BP	GO:0099587	inorganic ion import across plasma membrane	5/57	83/18670	5.37E-06	7.76E-05	5.13E-05	3764/10060/3757/3767/781	5
BP	GO:0002674	negative regulation of	3/57	12/1867	5.82E-06	8.36E-05	5.52E-05	5468/3630/134	3

		acute inflammatory response		0					
BP	GO:0050804	modulation of chemical synaptic transmission	9/57	436/18670	6.34E-06	9.04E-05	5.97E-05	148/154/3630/134/136/776/783/774/7124	9
BP	GO:0099177	regulation of trans-synaptic signaling	9/57	437/18670	6.46E-06	9.14E-05	6.04E-05	148/154/3630/134/136/776/783/774/7124	9
BP	GO:0051048	negative regulation of secretion	7/57	238/18670	7.70E-06	0.000108	7.15E-05	150/152/151/3630/134/3767/7124	7
BP	GO:1990573	potassium ion import across plasma membrane	4/57	43/18670	8.82E-06	0.000123	8.13E-05	3764/10060/3757/3767	4
BP	GO:0086069	bundle of His cell to Purkinje myocyte communication	3/57	14/18670	9.59E-06	0.000133	8.78E-05	6331/6336/781	3
BP	GO:0051924	regulation of calcium ion transport	7/57	254/18670	1.18E-05	0.000162	0.000107	150/801/775/776/783/774/781	7
BP	GO:0033604	negative regulation of catecholamine secretion	3/57	15/18670	1.20E-05	0.000164	0.000108	150/152/151	3
BP	GO:0009141	nucleoside triphosphate metabolic process	8/57	362/18670	1.33E-05	0.000181	0.000119	1349/4512/4513/4514/3630/50484/6391/6389	8
BP	GO:0007200	phospholipase C-activating G protein-coupled receptor signaling pathway	5/57	100/18670	1.34E-05	0.000181	0.000119	148/150/147/185/88	5
BP	GO:0010107	potassium ion import	4/57	48/18670	1.37E-05	0.000183	0.000121	3764/10060/3757/3767	4
BP	GO:0043268	positive regulation of	4/57	48/1867	1.37E-05	0.00018	0.000121	150/88/134/3757	4

		potassium ion transport		0		3			
BP	GO:0010469	regulation of signaling receptor activity	6/57	173/18670	1.42E-05	0.000189	0.000125	154/150/152/151/88/134	6
BP	GO:0014061	regulation of norepinephrine secretion	3/57	16/18670	1.47E-05	0.000193	0.000128	150/152/151	3
BP	GO:0009409	response to cold	4/57	50/18670	1.62E-05	0.00021	0.000139	4513/5468/154/153	4
BP	GO:0019233	sensory perception of pain	5/57	104/18670	1.62E-05	0.00021	0.000139	152/11280/55800/6336/134	5
BP	GO:0043266	regulation of potassium ion transport	5/57	105/18670	1.70E-05	0.000219	0.000145	150/88/134/776/3757	5
BP	GO:0031000	response to caffeine	3/57	17/18670	1.78E-05	0.000221	0.000146	5468/779/6261	3
BP	GO:0031649	heat generation	3/57	17/18670	1.78E-05	0.000221	0.000146	154/153/7124	3
BP	GO:0036270	response to diuretic	3/57	17/18670	1.78E-05	0.000221	0.000146	5468/779/6261	3
BP	GO:0048243	norepinephrine secretion	3/57	17/18670	1.78E-05	0.000221	0.000146	150/152/151	3
BP	GO:0061577	calcium ion transmembrane transport via high voltage-gated calcium channel	3/57	17/18670	1.78E-05	0.000221	0.000146	775/783/781	3
BP	GO:0099509	regulation of presynaptic cytosolic calcium ion concentration	3/57	17/18670	1.78E-05	0.000221	0.000146	134/776/783	3
BP	GO:2000649	regulation of sodium ion	4/57	52/1867	1.89E-05	0.00023	0.000155	6327/55800/6330/6324	4

		transmembrane transporter activity		0		4			
BP	GO:0098739	import across plasma membrane	5/57	110/18670	2.13E-05	0.000261	0.000173	3764/10060/3757/3767/781	5
BP	GO:0050994	regulation of lipid catabolic process	4/57	54/18670	2.20E-05	0.000269	0.000178	150/3630/134/7124	4
BP	GO:0017158	regulation of calcium ion-dependent exocytosis	5/57	112/18670	2.32E-05	0.000282	0.000186	148/150/136/776/783	5
BP	GO:0098900	regulation of action potential	4/57	55/18670	2.37E-05	0.000286	0.000189	148/6331/775/781	4
BP	GO:0010888	negative regulation of lipid storage	3/57	19/18670	2.53E-05	0.000303	0.0002	5468/1401/7124	3
BP	GO:0003014	renal system process	5/57	120/18670	3.23E-05	0.000386	0.000255	4879/148/185/134/50484	5
BP	GO:0015874	norepinephrine transport	3/57	21/18670	3.45E-05	0.000407	0.000269	150/152/151	3
BP	GO:0035813	regulation of renal sodium excretion	3/57	21/18670	3.45E-05	0.000407	0.000269	4879/185/134	3
BP	GO:0046034	ATP metabolic process	7/57	305/18670	3.81E-05	0.000447	0.000295	1349/4512/4513/4514/3630/6391/6389	7
BP	GO:0031281	positive regulation of cyclase activity	3/57	22/18670	3.99E-05	0.000465	0.000307	136/775/776	3
BP	GO:0009895	negative regulation of catabolic process	7/57	308/18670	4.05E-05	0.00047	0.00031	148/150/3630/134/7124/5290/6135	7
BP	GO:0034763	negative regulation of transmembrane transport	5/57	128/18670	4.41E-05	0.000508	0.000336	150/88/801/3757/7124	5
BP	GO:00358	renal sodium	3/57	23/1	4.58	0.00	0.00034	4879/185/134	3

	12	excretion		8670	E-05	0522	5		
BP	GO:0051349	positive regulation of lyase activity	3/57	23/18670	4.58E-05	0.000522	0.000345	136/775/776	3
BP	GO:0044062	regulation of excretion	3/57	24/18670	5.22E-05	0.000592	0.000391	4879/185/134	3
BP	GO:0045444	fat cell differentiation	6/57	223/18670	5.93E-05	0.000668	0.000441	5468/154/153/3630/7124/2033	6
BP	GO:0010460	positive regulation of heart rate	3/57	26/18670	6.67E-05	0.000749	0.000494	148/153/55800	3
BP	GO:0071902	positive regulation of protein serine/threonine kinase activity	7/57	334/18670	6.76E-05	0.000754	0.000498	154/150/151/801/134/136/7124	7
BP	GO:0009205	purine ribonucleoside triphosphate metabolic process	7/57	335/18670	6.88E-05	0.000764	0.000505	1349/4512/4513/4514/3630/6391/6389	7
BP	GO:1903779	regulation of cardiac conduction	4/57	73/18670	7.26E-05	0.000802	0.000529	801/10060/3767/6261	4
BP	GO:0009167	purine ribonucleoside monophosphate metabolic process	7/57	340/18670	7.55E-05	0.000829	0.000548	1349/4512/4513/4514/3630/6391/6389	7
BP	GO:0009126	purine nucleoside monophosphate metabolic process	7/57	341/18670	7.69E-05	0.000836	0.000552	1349/4512/4513/4514/3630/6391/6389	7
BP	GO:0009199	ribonucleoside triphosphate metabolic process	7/57	341/18670	7.69E-05	0.000836	0.000552	1349/4512/4513/4514/3630/6391/6389	7
BP	GO:0009144	purine nucleoside	7/57	342/186	7.84E-05	0.00084	0.000559	1349/4512/4513/4514/3630/639	7

		triphosphate metabolic process		70		7		1/6389	
BP	GO:0045834	positive regulation of lipid metabolic process	5/57	146/18670	8.24E-05	0.000886	0.000585	5468/185/3630/134/7124	5
BP	GO:0042593	glucose homeostasis	6/57	241/18670	9.10E-05	0.000973	0.000642	5468/150/147/3630/3767/5290	6
BP	GO:0033500	carbohydrate homeostasis	6/57	242/18670	9.31E-05	0.00099	0.000654	5468/150/147/3630/3767/5290	6
BP	GO:2000300	regulation of synaptic vesicle exocytosis	4/57	78/18670	9.41E-05	0.000996	0.000658	148/136/776/783	4
BP	GO:0009266	response to temperature stimulus	6/57	243/18670	9.52E-05	0.001002	0.000662	4513/5468/154/153/134/2033	6
BP	GO:1903169	regulation of calcium ion transmembrane transport	5/57	151/18670	9.66E-05	0.001012	0.000668	150/801/775/783/781	5
BP	GO:0009161	ribonucleoside monophosphate metabolic process	7/57	354/18670	9.72E-05	0.001013	0.000669	1349/4512/4513/4514/3630/6391/6389	7
BP	GO:0062013	positive regulation of small molecule metabolic process	5/57	153/18670	0.000103	0.001066	0.000704	4513/5468/3630/136/7124	5
BP	GO:0010743	regulation of macrophage derived foam cell differentiation	3/57	30/18670	0.000103	0.001066	0.000704	5468/185/1401	3
BP	GO:0048871	multicellular organismal homeostasis	8/57	485/18670	0.000106	0.001083	0.000715	6337/6340/154/153/6338/134/7124/5290	8
BP	GO:0017156	calcium ion regulated exocytosis	5/57	154/18670	0.000106	0.001083	0.000715	148/150/136/776/783	5

BP	GO:0086011	membrane repolarization during action potential	3/57	31/18670	0.000114	0.00116	0.000766	3764/3757/781	3
BP	GO:0043271	negative regulation of ion transport	5/57	157/18670	0.000116	0.001174	0.000775	150/88/801/134/3757	5
BP	GO:0060402	calcium ion transport into cytosol	5/57	158/18670	0.00012	0.001204	0.000795	148/801/775/6261/781	5
BP	GO:1902803	regulation of synaptic vesicle transport	4/57	84/18670	0.000126	0.001246	0.000823	148/136/776/783	4
BP	GO:0045907	positive regulation of vasoconstriction	3/57	32/18670	0.000126	0.001246	0.000823	148/147/152	3
BP	GO:0045987	positive regulation of smooth muscle contraction	3/57	32/18670	0.000126	0.001246	0.000823	148/147/151	3
BP	GO:1903305	regulation of regulated secretory pathway	5/57	160/18670	0.000127	0.001252	0.000827	148/150/136/776/783	5
BP	GO:0072659	protein localization to plasma membrane	6/57	260/18670	0.000138	0.001354	0.000894	3630/88/55800/783/3767/7124	6
BP	GO:0009123	nucleoside monophosphate metabolic process	7/57	375/18670	0.000139	0.001358	0.000897	1349/4512/4513/4514/3630/6391/6389	7
BP	GO:0006099	tricarboxylic acid cycle	3/57	34/18670	0.000151	0.001462	0.000965	6390/6391/6389	3
BP	GO:0070050	neuron cellular homeostasis	3/57	34/18670	0.000151	0.001462	0.000965	134/776/783	3
BP	GO:1901019	regulation of calcium ion transmembrane transporter	4/57	89/18670	0.000157	0.001515	0.001	150/801/783/781	4

		activity							
BP	GO:1901653	cellular response to peptide	7/57	385/18670	0.000163	0.001559	0.001029	5468/154/185/3630/88/5290/781	7
BP	GO:0007269	neurotransmitter secretion	5/57	169/18670	0.000164	0.001559	0.001029	148/136/776/783/774	5
BP	GO:0006101	citrate metabolic process	3/57	35/18670	0.000165	0.001559	0.001029	6390/6391/6389	3
BP	GO:0098801	regulation of renal system process	3/57	35/18670	0.000165	0.001559	0.001029	4879/185/134	3
BP	GO:0099643	signal release from synapse	5/57	170/18670	0.000169	0.001587	0.001048	148/136/776/783/774	5
BP	GO:1904063	negative regulation of cation transmembrane transport	4/57	91/18670	0.000171	0.001605	0.00106	150/88/801/3757	4
BP	GO:0060401	cytosolic calcium ion transport	5/57	171/18670	0.000173	0.001616	0.001067	148/801/775/6261/781	5
BP	GO:0045833	negative regulation of lipid metabolic process	4/57	92/18670	0.000179	0.001659	0.001095	150/3630/134/7124	4
BP	GO:0014808	release of sequestered calcium ion into cytosol by sarcoplasmic reticulum	3/57	36/18670	0.000179	0.001659	0.001095	801/775/6261	3
BP	GO:0043243	positive regulation of protein complex disassembly	3/57	37/18670	0.000195	0.001785	0.001179	154/88/7124	3
BP	GO:1903514	release of sequestered calcium ion into cytosol by endoplasmic	3/57	37/18670	0.000195	0.001785	0.001179	801/775/6261	3

		reticulum							
BP	GO:0051952	regulation of amine transport	4/57	95/18670	0.000202	0.001844	0.001218	150/152/151/134	4
BP	GO:0007190	activation of adenylate cyclase activity	3/57	38/18670	0.000211	0.001908	0.00126	154/153/136	3
BP	GO:1901021	positive regulation of calcium ion transmembrane transporter activity	3/57	38/18670	0.000211	0.001908	0.00126	801/783/781	3
BP	GO:0051930	regulation of sensory perception of pain	3/57	39/18670	0.000228	0.002045	0.00135	152/11280/134	3
BP	GO:0072350	tricarboxylic acid metabolic process	3/57	39/18670	0.000228	0.002045	0.00135	6390/6391/6389	3
BP	GO:0045823	positive regulation of heart contraction	3/57	40/18670	0.000246	0.002187	0.001444	148/153/55800	3
BP	GO:0051931	regulation of sensory perception	3/57	40/18670	0.000246	0.002187	0.001444	152/11280/134	3
BP	GO:0050873	brown fat cell differentiation	3/57	41/18670	0.000265	0.00233	0.001539	154/153/3630	3
BP	GO:0015837	amine transport	4/57	102/18670	0.000266	0.00233	0.001539	150/152/151/134	4
BP	GO:2000379	positive regulation of reactive oxygen species metabolic process	4/57	102/18670	0.000266	0.00233	0.001539	4513/185/1401/7124	4
BP	GO:0034766	negative regulation of ion transmembran	4/57	103/18670	0.000276	0.002408	0.00159	150/88/801/3757	4

		e transport						
BP	GO:0046928	regulation of neurotransmitter secretion	4/57	104/18670	0.000286	0.002488	0.001643	148/136/776/783	4
BP	GO:0031279	regulation of cyclase activity	3/57	43/18670	0.000305	0.002623	0.001732	136/775/776	3
BP	GO:0070296	sarcoplasmic reticulum calcium ion transport	3/57	43/18670	0.000305	0.002623	0.001732	801/775/6261	3
BP	GO:0086004	regulation of cardiac muscle cell contraction	3/57	43/18670	0.000305	0.002623	0.001732	6331/134/775	3
BP	GO:0051047	positive regulation of secretion	7/57	428/18670	0.000312	0.002666	0.00176	4879/3630/134/136/776/783/7124	7
BP	GO:2000377	regulation of reactive oxygen species metabolic process	5/57	195/18670	0.000318	0.002705	0.001787	4513/185/3630/1401/7124	5
BP	GO:0051341	regulation of oxidoreductase activity	4/57	107/18670	0.000319	0.002705	0.001787	185/3630/801/7124	4
BP	GO:1990778	protein localization to cell periphery	6/57	311/18670	0.000362	0.003056	0.002018	3630/88/55800/783/3767/7124	6
BP	GO:0010883	regulation of lipid storage	3/57	46/18670	0.000373	0.003101	0.002048	5468/1401/7124	3
BP	GO:0030810	positive regulation of nucleotide biosynthetic process	3/57	46/18670	0.000373	0.003101	0.002048	4513/3630/136	3
BP	GO:0051339	regulation of lyase activity	3/57	46/18670	0.000373	0.003101	0.002048	136/775/776	3
BP	GO:1900373	positive regulation of purine nucleotide	3/57	46/18670	0.000373	0.003101	0.002048	4513/3630/136	3

		biosynthetic process							
BP	GO:0098693	regulation of synaptic vesicle cycle	4/57	112/18670	0.000379	0.003141	0.002074	148/136/776/783	4
BP	GO:0006953	acute-phase response	3/57	47/18670	0.000398	0.003266	0.002157	3630/1401/7124	3
BP	GO:1903115	regulation of actin filament-based movement	3/57	47/18670	0.000398	0.003266	0.002157	6331/134/775	3
BP	GO:0044557	relaxation of smooth muscle	2/57	10/18670	0.000406	0.003279	0.002165	134/136	2
BP	GO:0045741	positive regulation of epidermal growth factor-activated receptor activity	2/57	10/18670	0.000406	0.003279	0.002165	150/134	2
BP	GO:0070472	regulation of uterine smooth muscle contraction	2/57	10/18670	0.000406	0.003279	0.002165	152/151	2
BP	GO:0071313	cellular response to caffeine	2/57	10/18670	0.000406	0.003279	0.002165	779/6261	2
BP	GO:0045933	positive regulation of muscle contraction	3/57	48/18670	0.000423	0.003408	0.002251	148/147/151	3
BP	GO:0006006	glucose metabolic process	5/57	209/18670	0.000437	0.003507	0.002316	3630/3767/7124/5290/2033	5
BP	GO:0016079	synaptic vesicle exocytosis	4/57	119/18670	0.000478	0.003815	0.002519	148/136/776/783	4
BP	GO:0033860	regulation of NAD(P)H oxidase activity	2/57	11/18670	0.000495	0.003866	0.002553	185/3630	2
BP	GO:00400	negative	2/57	11/1	0.00	0.00	0.00255	154/153	2

	15	regulation of multicellular organism growth		8670	0495	3866	3		
BP	GO:0070471	uterine smooth muscle contraction	2/57	11/18670	0.000495	0.003866	0.002553	152/151	2
BP	GO:0099624	atrial cardiac muscle cell membrane repolarization	2/57	11/18670	0.000495	0.003866	0.002553	6331/776	2
BP	GO:1902805	positive regulation of synaptic vesicle transport	2/57	11/18670	0.000495	0.003866	0.002553	776/783	2
BP	GO:0051235	maintenance of location	6/57	330/18670	0.000495	0.003866	0.002553	5468/801/775/1401/7124/6261	6
BP	GO:0050999	regulation of nitric-oxide synthase activity	3/57	51/18670	0.000506	0.003938	0.002601	3630/801/7124	3
BP	GO:0017157	regulation of exocytosis	5/57	217/18670	0.000519	0.00402	0.002655	148/150/136/776/783	5
BP	GO:0051928	positive regulation of calcium ion transport	4/57	123/18670	0.000541	0.004176	0.002758	801/776/783/781	4
BP	GO:0002526	acute inflammatory response	5/57	220/18670	0.000552	0.004247	0.002805	5468/3630/134/1401/7124	5
BP	GO:1903307	positive regulation of regulated secretory pathway	3/57	53/18670	0.000567	0.004345	0.002869	136/776/783	3
BP	GO:0002024	diet induced thermogenesis	2/57	12/18670	0.000593	0.00449	0.002965	154/153	2
BP	GO:0045760	positive regulation of action	2/57	12/18670	0.000593	0.00449	0.002965	148/6331	2

		potential							
BP	GO:1901387	positive regulation of voltage-gated calcium channel activity	2/57	12/18670	0.000593	0.00449	0.002965	783/781	2
BP	GO:0051353	positive regulation of oxidoreductase activity	3/57	54/18670	0.000599	0.004506	0.002976	185/3630/7124	3
BP	GO:1904645	response to amyloid-beta	3/57	54/18670	0.000599	0.004506	0.002976	154/774/781	3
BP	GO:0061098	positive regulation of protein tyrosine kinase activity	3/57	55/18670	0.000632	0.004739	0.003129	148/150/134	3
BP	GO:0001973	adenosine receptor signaling pathway	2/57	13/18670	0.000699	0.00515	0.003401	134/136	2
BP	GO:0003084	positive regulation of systemic arterial blood pressure	2/57	13/18670	0.000699	0.00515	0.003401	148/134	2
BP	GO:0010745	negative regulation of macrophage derived foam cell differentiation	2/57	13/18670	0.000699	0.00515	0.003401	5468/1401	2
BP	GO:0071415	cellular response to purine-containing compound	2/57	13/18670	0.000699	0.00515	0.003401	779/6261	2
BP	GO:0045981	positive regulation of nucleotide metabolic process	3/57	57/18670	0.000702	0.00515	0.003401	4513/3630/136	3
BP	GO:19005	positive	3/57	57/1	0.00	0.00	0.00340	4513/3630/136	3

	44	regulation of purine nucleotide metabolic process		8670	0702	515	1		
BP	GO:0001505	regulation of neurotransmitter levels	6/57	354/18670	0.000716	0.005229	0.003453	148/136/776/783/774/7124	6
BP	GO:0031644	regulation of neurological system process	4/57	137/18670	0.000811	0.005884	0.003886	154/152/11280/134	4
BP	GO:0038166	angiotensin-activated signaling pathway	2/57	14/18670	0.000814	0.005884	0.003886	185/88	2
BP	GO:0099171	presynaptic modulation of chemical synaptic transmission	2/57	14/18670	0.000814	0.005884	0.003886	776/783	2
BP	GO:0051588	regulation of neurotransmitter transport	4/57	139/18670	0.000856	0.006148	0.00406	148/136/776/783	4
BP	GO:1905953	negative regulation of lipid localization	3/57	61/18670	0.000856	0.006148	0.00406	5468/1401/7124	3
BP	GO:0097553	calcium ion transmembrane import into cytosol	4/57	140/18670	0.000879	0.006289	0.004153	801/775/6261/781	4
BP	GO:0050433	regulation of catecholamine secretion	3/57	62/18670	0.000898	0.00638	0.004213	150/152/151	3
BP	GO:0070265	necrotic cell death	3/57	62/18670	0.000898	0.00638	0.004213	4513/291/7124	3
BP	GO:0050709	negative regulation of protein secretion	4/57	141/18670	0.000903	0.006391	0.004221	150/3630/3767/7124	4
BP	GO:0030730	sequestering of triglyceride	2/57	15/18670	0.000937	0.006523	0.004308	5468/7124	2

BP	GO:0051044	positive regulation of membrane protein ectodomain proteolysis	2/57	15/18670	0.000937	0.006523	0.004308	150/7124	2
BP	GO:0055089	fatty acid homeostasis	2/57	15/18670	0.000937	0.006523	0.004308	3630/134	2
BP	GO:1901841	regulation of high voltage-gated calcium channel activity	2/57	15/18670	0.000937	0.006523	0.004308	783/781	2
BP	GO:1902514	regulation of calcium ion transmembrane transport via high voltage-gated calcium channel	2/57	15/18670	0.000937	0.006523	0.004308	783/781	2
BP	GO:0019318	hexose metabolic process	5/57	249/18670	0.000964	0.006689	0.004418	3630/3767/7124/5290/2033	5
BP	GO:0032768	regulation of monooxygenase activity	3/57	64/18670	0.000985	0.006786	0.004482	3630/801/7124	3
BP	GO:0050432	catecholamine secretion	3/57	64/18670	0.000985	0.006786	0.004482	150/152/151	3
BP	GO:0035264	multicellular organism growth	4/57	146/18670	0.001028	0.007057	0.00466	154/153/5290/2033	4
BP	GO:0040014	regulation of multicellular organism growth	3/57	66/18670	0.001077	0.007372	0.004868	154/153/5290	3
BP	GO:0002792	negative regulation of peptide secretion	4/57	148/18670	0.001081	0.007373	0.004869	150/3630/3767/7124	4
BP	GO:00140	response to	4/57	149/	0.00	0.00	0.00497	5468/3767/779/	4

	74	purine-containing compound		18670	1108	7534	5	6261	
BP	GO:0050909	sensory perception of taste	3/57	67/18670	0.001125	0.007625	0.005035	6337/6340/6338	3
BP	GO:0043406	positive regulation of MAP kinase activity	5/57	258/18670	0.00113	0.00763	0.005039	150/151/134/136/7124	5
BP	GO:0016999	antibiotic metabolic process	4/57	151/18670	0.001164	0.007837	0.005176	4513/6390/6391/6389	4
BP	GO:0019915	lipid storage	3/57	68/18670	0.001175	0.007856	0.005188	5468/1401/7124	3
BP	GO:0050891	multicellular organismal water homeostasis	3/57	68/18670	0.001175	0.007856	0.005188	6337/6340/6338	3
BP	GO:0048489	synaptic vesicle transport	4/57	152/18670	0.001193	0.007909	0.005223	148/136/776/783	4
BP	GO:0097480	establishment of synaptic vesicle localization	4/57	152/18670	0.001193	0.007909	0.005223	148/136/776/783	4
BP	GO:0017085	response to insecticide	2/57	17/18670	0.001209	0.007909	0.005223	6327/6324	2
BP	GO:0032460	negative regulation of protein oligomerization	2/57	17/18670	0.001209	0.007909	0.005223	3630/2033	2
BP	GO:0045721	negative regulation of gluconeogenesis	2/57	17/18670	0.001209	0.007909	0.005223	3630/2033	2
BP	GO:0048521	negative regulation of behavior	2/57	17/18670	0.001209	0.007909	0.005223	3630/134	2
BP	GO:0015850	organic hydroxy compound	5/57	262/18670	0.00121	0.007909	0.005223	5468/150/152/185/151	5

		transport							
BP	GO:0030168	platelet activation	4/57	153/18670	0.001222	0.007967	0.005261	150/152/151/5290	4
BP	GO:1904427	positive regulation of calcium ion transmembrane transport	3/57	71/18670	0.001331	0.008648	0.005711	801/783/781	3
BP	GO:1905952	regulation of lipid localization	4/57	157/18670	0.001344	0.008707	0.00575	5468/185/1401/7124	4
BP	GO:0006836	neurotransmitter transport	5/57	269/18670	0.001359	0.008776	0.005795	148/136/776/783/774	5
BP	GO:0002673	regulation of acute inflammatory response	4/57	159/18670	0.001409	0.009066	0.005987	5468/3630/134/7124	4
BP	GO:0030104	water homeostasis	3/57	74/18670	0.0015	0.009623	0.006355	6337/6340/6338	3
BP	GO:0002544	chronic inflammatory response	2/57	19/18670	0.001515	0.009657	0.006377	136/7124	2
BP	GO:0098915	membrane repolarization during ventricular cardiac muscle cell action potential	2/57	19/18670	0.001515	0.009657	0.006377	3764/3757	2
BP	GO:0019216	regulation of lipid metabolic process	6/57	410/18670	0.001524	0.009689	0.006398	5468/150/185/3630/134/7124	6
BP	GO:0097479	synaptic vesicle localization	4/57	163/18670	0.001543	0.009749	0.006438	148/136/776/783	4
BP	GO:1902600	proton transmembrane transport	4/57	163/18670	0.001543	0.009749	0.006438	1349/4512/4513/4514	4
BP	GO:0051937	catecholamine transport	3/57	77/18670	0.001682	0.01059	0.006994	150/152/151	3

BP	GO:0072593	reactive oxygen species metabolic process	5/57	284/18670	0.001726	0.010835	0.007155	4513/185/3630/1401/7124	5
BP	GO:0032413	negative regulation of ion transmembrane transporter activity	3/57	78/18670	0.001745	0.01089	0.007192	150/88/801	3
BP	GO:0072332	intrinsic apoptotic signaling pathway by p53 class mediator	3/57	78/18670	0.001745	0.01089	0.007192	50484/6135/2033	3
BP	GO:0001956	positive regulation of neurotransmitter secretion	2/57	21/18670	0.001853	0.011527	0.007612	776/783	2
BP	GO:0097193	intrinsic apoptotic signaling pathway	5/57	289/18670	0.001863	0.011553	0.00763	3630/50484/7124/6135/2033	5
BP	GO:0001659	temperature homeostasis	4/57	173/18670	0.001919	0.011866	0.007836	154/153/134/7124	4
BP	GO:0005996	monosaccharide metabolic process	5/57	292/18670	0.001949	0.012013	0.007933	3630/3767/7124/5290/2033	5
BP	GO:0051702	interaction with symbiont	3/57	82/18670	0.002014	0.01238	0.008176	801/5340/2033	3
BP	GO:0005980	glycogen catabolic process	2/57	22/18670	0.002034	0.012428	0.008208	3630/801	2
BP	GO:0051000	positive regulation of nitric-oxide synthase activity	2/57	22/18670	0.002034	0.012428	0.008208	3630/7124	2
BP	GO:0009251	glucan catabolic process	2/57	23/18670	0.002223	0.013505	0.008919	3630/801	2

BP	GO:0051043	regulation of membrane protein ectodomain proteolysis	2/57	23/18670	0.002223	0.013505	0.008919	150/7124	2
BP	GO:0014068	positive regulation of phosphatidylinositol 3-kinase signaling	3/57	87/18670	0.002385	0.014443	0.009538	3630/7124/5290	3
BP	GO:0010881	regulation of cardiac muscle contraction by regulation of the release of sequestered calcium ion	2/57	24/18670	0.002421	0.014532	0.009597	801/775	2
BP	GO:0035588	G protein-coupled purinergic receptor signaling pathway	2/57	24/18670	0.002421	0.014532	0.009597	134/136	2
BP	GO:0086064	cell communication by electrical coupling involved in cardiac conduction	2/57	24/18670	0.002421	0.014532	0.009597	801/775	2
BP	GO:0045921	positive regulation of exocytosis	3/57	88/18670	0.002464	0.014703	0.00971	136/776/783	3
BP	GO:0061097	regulation of protein tyrosine kinase activity	3/57	88/18670	0.002464	0.014703	0.00971	148/150/134	3
BP	GO:0032410	negative regulation of transporter activity	3/57	89/18670	0.002544	0.01514	0.009998	150/88/801	3
BP	GO:0003071	renal system process involved in	2/57	25/18670	0.002626	0.015361	0.010144	185/134	2

		regulation of systemic arterial blood pressure							
BP	GO:0044247	cellular polysaccharide catabolic process	2/57	25/18670	0.002626	0.015361	0.010144	3630/801	2
BP	GO:0045956	positive regulation of calcium ion-dependent exocytosis	2/57	25/18670	0.002626	0.015361	0.010144	776/783	2
BP	GO:1904385	cellular response to angiotensin	2/57	25/18670	0.002626	0.015361	0.010144	185/88	2
BP	GO:0015844	monoamine transport	3/57	90/18670	0.002626	0.015361	0.010144	150/152/151	3
BP	GO:1901379	regulation of potassium ion transmembrane transport	3/57	90/18670	0.002626	0.015361	0.010144	88/776/3757	3
BP	GO:0062012	regulation of small molecule metabolic process	6/57	459/18670	0.002687	0.015671	0.010349	4513/5468/3630/136/7124/2033	6
BP	GO:0050731	positive regulation of peptidyl-tyrosine phosphorylation	4/57	192/18670	0.002799	0.016278	0.01075	148/150/134/7124	4
BP	GO:0031954	positive regulation of protein autophosphorylation	2/57	26/18670	0.002839	0.016421	0.010844	3630/801	2
BP	GO:0032800	receptor biosynthetic process	2/57	26/18670	0.002839	0.016421	0.010844	5468/7124	2
BP	GO:0051224	negative regulation of protein	4/57	193/18670	0.002852	0.016446	0.010861	150/3630/3767/7124	4

		transport							
BP	GO:0007589	body fluid secretion	3/57	93/18670	0.002882	0.016575	0.010946	4513/4879/134	3
BP	GO:0099504	synaptic vesicle cycle	4/57	194/18670	0.002905	0.01666	0.011002	148/136/776/783	4
BP	GO:0071375	cellular response to peptide hormone stimulus	5/57	321/18670	0.002935	0.016786	0.011085	5468/185/3630/88/5290	5
BP	GO:0000272	polysaccharide catabolic process	2/57	27/18670	0.003061	0.017265	0.011402	3630/801	2
BP	GO:0002021	response to dietary excess	2/57	27/18670	0.003061	0.017265	0.011402	154/153	2
BP	GO:0002026	regulation of the force of heart contraction	2/57	27/18670	0.003061	0.017265	0.011402	148/153	2
BP	GO:0007176	regulation of epidermal growth factor-activated receptor activity	2/57	27/18670	0.003061	0.017265	0.011402	150/134	2
BP	GO:1903076	regulation of protein localization to plasma membrane	3/57	95/18670	0.003061	0.017265	0.011402	3630/3767/7124	3
BP	GO:1904950	negative regulation of establishment of protein localization	4/57	197/18670	0.00307	0.017267	0.011403	150/3630/3767/7124	4
BP	GO:0050769	positive regulation of neurogenesis	6/57	474/18670	0.00315	0.017669	0.011668	5468/152/151/6324/7124/2033	6
BP	GO:0032770	positive regulation of monooxygenas	2/57	28/18670	0.00329	0.018253	0.012054	3630/7124	2

		e activity							
BP	GO:0045761	regulation of adenylate cyclase activity	2/57	28/18670	0.00329	0.018253	0.012054	775/776	2
BP	GO:0086013	membrane repolarization during cardiac muscle cell action potential	2/57	28/18670	0.00329	0.018253	0.012054	3764/3757	2
BP	GO:1990776	response to angiotensin	2/57	28/18670	0.00329	0.018253	0.012054	185/88	2
BP	GO:0050727	regulation of inflammatory response	6/57	485/18670	0.003526	0.019357	0.012784	5468/185/3630/134/136/7124	6
BP	GO:0010644	cell communication by electrical coupling	2/57	29/18670	0.003526	0.019357	0.012784	801/775	2
BP	GO:0010882	regulation of cardiac muscle contraction by calcium ion signaling	2/57	29/18670	0.003526	0.019357	0.012784	801/775	2
BP	GO:0045822	negative regulation of heart contraction	2/57	29/18670	0.003526	0.019357	0.012784	148/134	2
BP	GO:0007596	blood coagulation	5/57	336/18670	0.003568	0.019533	0.0129	150/152/151/5290/5340	5
BP	GO:0043405	regulation of MAP kinase activity	5/57	337/18670	0.003613	0.019729	0.013029	150/151/134/136/7124	5
BP	GO:1903426	regulation of reactive oxygen species biosynthetic process	3/57	101/18670	0.003638	0.019813	0.013084	4513/3630/7124	3
BP	GO:0099003	vesicle-mediated transport in synapse	4/57	207/18670	0.003664	0.019903	0.013144	148/136/776/783	4

BP	GO:0000002	mitochondrial genome maintenance	2/57	30/18670	0.003771	0.020373	0.013454	291/50484	2
BP	GO:0045940	positive regulation of steroid metabolic process	2/57	30/18670	0.003771	0.020373	0.013454	185/7124	2
BP	GO:0007599	hemostasis	5/57	341/18670	0.003799	0.020472	0.013519	150/152/151/5290/5340	5
BP	GO:0050817	coagulation	5/57	342/18670	0.003847	0.020673	0.013653	150/152/151/5290/5340	5
BP	GO:0045742	positive regulation of epidermal growth factor receptor signaling pathway	2/57	31/18670	0.004023	0.021453	0.014167	150/134	2
BP	GO:0090075	relaxation of muscle	2/57	31/18670	0.004023	0.021453	0.014167	134/136	2
BP	GO:1902253	regulation of intrinsic apoptotic signaling pathway by p53 class mediator	2/57	31/18670	0.004023	0.021453	0.014167	50484/6135	2
BP	GO:0032388	positive regulation of intracellular transport	4/57	215/18670	0.004193	0.022302	0.014728	88/136/776/783	4
BP	GO:0010880	regulation of release of sequestered calcium ion into cytosol by sarcoplasmic reticulum	2/57	32/18670	0.004283	0.022604	0.014927	801/775	2
BP	GO:0032228	regulation of synaptic	2/57	32/1867	0.00428	0.02260	0.014927	148/134	2

		transmission, GABAergic		0	3	4			
BP	GO:0035587	purinergic receptor signaling pathway	2/57	32/18670	0.004283	0.022604	0.014927	134/136	2
BP	GO:0019722	calcium-mediated signaling	4/57	218/18670	0.004404	0.023184	0.015311	185/801/775/7124	4
BP	GO:0019934	cGMP-mediated signaling	2/57	33/18670	0.00455	0.02371	0.015658	4879/136	2
BP	GO:0051194	positive regulation of cofactor metabolic process	2/57	33/18670	0.00455	0.02371	0.015658	4513/3630	2
BP	GO:1901186	positive regulation of ERBB signaling pathway	2/57	33/18670	0.00455	0.02371	0.015658	150/134	2
BP	GO:1901380	negative regulation of potassium ion transmembrane transport	2/57	33/18670	0.00455	0.02371	0.015658	88/3757	2
BP	GO:0043279	response to alkaloid	3/57	112/18670	0.00486	0.025258	0.016681	5468/779/6261	3
BP	GO:0043244	regulation of protein complex disassembly	3/57	113/18670	0.004982	0.025826	0.017056	154/88/7124	3
BP	GO:0071312	cellular response to alkaloid	2/57	35/18670	0.005108	0.026347	0.0174	779/6261	2
BP	GO:1901385	regulation of voltage-gated calcium channel activity	2/57	35/18670	0.005108	0.026347	0.0174	783/781	2
BP	GO:1904375	regulation of protein	3/57	115/186	0.00523	0.02691	0.017774	3630/3767/7124	3

		localization to cell periphery		70	1	4			
BP	GO:0006909	phagocytosis	5/57	369/18670	0.005297	0.02712	0.01791	5468/134/1401/7124/5290	5
BP	GO:0035690	cellular response to drug	5/57	369/18670	0.005297	0.02712	0.01791	3757/3767/779/7124/6261	5
BP	GO:0090322	regulation of superoxide metabolic process	2/57	36/18670	0.005398	0.027429	0.018114	1401/7124	2
BP	GO:1901020	negative regulation of calcium ion transmembrane transporter activity	2/57	36/18670	0.005398	0.027429	0.018114	150/801	2
BP	GO:2001171	positive regulation of ATP biosynthetic process	2/57	36/18670	0.005398	0.027429	0.018114	4513/3630	2
BP	GO:1900371	regulation of purine nucleotide biosynthetic process	3/57	117/18670	0.005487	0.027816	0.01837	4513/3630/136	3
BP	GO:0030808	regulation of nucleotide biosynthetic process	3/57	118/18670	0.005618	0.02841	0.018762	4513/3630/136	3
BP	GO:0007173	epidermal growth factor receptor signaling pathway	3/57	119/18670	0.005751	0.029011	0.019159	150/134/5290	3
BP	GO:0010939	regulation of necrotic cell death	2/57	38/18670	0.006	0.029969	0.019791	4513/291	2
BP	GO:0046676	negative regulation of insulin secretion	2/57	38/18670	0.006	0.029969	0.019791	150/3767	2

BP	GO:0051590	positive regulation of neurotransmitter transport	2/57	38/18670	0.006	0.029969	0.019791	776/783	2
BP	GO:1903523	negative regulation of blood circulation	2/57	38/18670	0.006	0.029969	0.019791	148/134	2
BP	GO:1903409	reactive oxygen species biosynthetic process	3/57	122/18670	0.006162	0.030703	0.020276	4513/3630/7124	3
BP	GO:0009165	nucleotide biosynthetic process	5/57	386/18670	0.006388	0.031756	0.020972	4513/4879/3630/136/50484	5
BP	GO:0014066	regulation of phosphatidylinositol 3-kinase signaling	3/57	124/18670	0.006444	0.031881	0.021054	3630/7124/5290	3
BP	GO:0051209	release of sequestered calcium ion into cytosol	3/57	124/18670	0.006444	0.031881	0.021054	801/775/6261	3
BP	GO:2000008	regulation of protein localization to cell surface	2/57	40/18670	0.006631	0.032724	0.021611	88/7124	2
BP	GO:1901293	nucleoside phosphate biosynthetic process	5/57	390/18670	0.006667	0.032821	0.021675	4513/4879/3630/136/50484	5
BP	GO:0051283	negative regulation of sequestering of calcium ion	3/57	126/18670	0.006735	0.033079	0.021845	801/775/6261	3
BP	GO:0043267	negative regulation of potassium ion transport	2/57	41/18670	0.006957	0.033927	0.022405	88/3757	2
BP	GO:1901381	positive regulation of potassium ion transmembrane transport	2/57	41/18670	0.006957	0.033927	0.022405	88/3757	2

BP	GO:1903170	negative regulation of calcium ion transmembrane transport	2/57	41/18670	0.006957	0.033927	0.022405	150/801	2
BP	GO:0045926	negative regulation of growth	4/57	249/18670	0.007017	0.034055	0.02249	4879/5468/154/153	4
BP	GO:0002576	platelet degranulation	3/57	128/18670	0.007033	0.034055	0.02249	88/801/5340	3
BP	GO:0051282	regulation of sequestering of calcium ion	3/57	128/18670	0.007033	0.034055	0.02249	801/775/6261	3
BP	GO:0006509	membrane protein ectodomain proteolysis	2/57	42/18670	0.007291	0.035137	0.023204	150/7124	2
BP	GO:0010677	negative regulation of cellular carbohydrate metabolic process	2/57	42/18670	0.007291	0.035137	0.023204	3630/2033	2
BP	GO:1903532	positive regulation of secretion by cell	5/57	399/18670	0.007323	0.035211	0.023253	3630/136/776/783/7124	5
BP	GO:0051208	sequestering of calcium ion	3/57	131/18670	0.007495	0.035953	0.023743	801/775/6261	3
BP	GO:0032459	regulation of protein oligomerization	2/57	43/18670	0.007632	0.036353	0.024007	3630/2033	2
BP	GO:0050850	positive regulation of calcium-mediated signaling	2/57	43/18670	0.007632	0.036353	0.024007	801/7124	2
BP	GO:1904646	cellular response to amyloid-beta	2/57	43/18670	0.007632	0.036353	0.024007	154/781	2
BP	GO:0045598	regulation of fat cell	3/57	132/186	0.00765	0.03637	0.024019	5468/3630/7124	3

		differentiation		70	3	1			
BP	GO:0050730	regulation of peptidyl-tyrosine phosphorylation	4/57	256/18670	0.007723	0.036616	0.024181	148/150/134/7124	4
BP	GO:0071333	cellular response to glucose stimulus	3/57	133/18670	0.007813	0.036959	0.024408	150/3767/5290	3
BP	GO:0035307	positive regulation of protein dephosphorylation	2/57	44/18670	0.00798	0.037574	0.024813	801/134	2
BP	GO:0070266	necroptotic process	2/57	44/18670	0.00798	0.037574	0.024813	291/7124	2
BP	GO:0071331	cellular response to hexose stimulus	3/57	135/18670	0.008139	0.038235	0.02525	150/3767/5290	3
BP	GO:0051091	positive regulation of DNA-binding transcription factor activity	4/57	261/18670	0.008255	0.038624	0.025507	5468/3630/7124/2033	4
BP	GO:0071326	cellular response to monosaccharide stimulus	3/57	136/18670	0.008305	0.038624	0.025507	150/3767/5290	3
BP	GO:0044275	cellular carbohydrate catabolic process	2/57	45/18670	0.008334	0.038624	0.025507	3630/801	2
BP	GO:0051932	synaptic transmission, GABAergic	2/57	45/18670	0.008334	0.038624	0.025507	148/134	2
BP	GO:0090278	negative regulation of peptide hormone secretion	2/57	45/18670	0.008334	0.038624	0.025507	150/3767	2
BP	GO:19002	regulation of	2/57	45/1	0.00	0.03	0.02550	3630/134	2

	71	long-term synaptic potentiation		8670	8334	8624	7		
BP	GO:0031330	negative regulation of cellular catabolic process	4/57	264/18670	0.008585	0.039697	0.026216	148/3630/5290/6135	4
BP	GO:0035304	regulation of protein dephosphorylation	3/57	139/18670	0.008814	0.040662	0.026853	801/134/7124	3
BP	GO:0060078	regulation of postsynaptic membrane potential	3/57	140/18670	0.008988	0.041371	0.027321	154/153/134	3
BP	GO:0031952	regulation of protein autophosphorylation	2/57	47/18670	0.009065	0.041542	0.027434	3630/801	2
BP	GO:1903580	positive regulation of ATP metabolic process	2/57	47/18670	0.009065	0.041542	0.027434	4513/3630	2
BP	GO:0009896	positive regulation of catabolic process	5/57	423/18670	0.009295	0.042406	0.028004	154/150/3630/134/7124	5
BP	GO:0032386	regulation of intracellular transport	5/57	423/18670	0.009295	0.042406	0.028004	148/88/136/776/783	5
BP	GO:0038127	ERBB signaling pathway	3/57	142/18670	0.009341	0.042428	0.028019	150/134/5290	3
BP	GO:1900542	regulation of purine nucleotide metabolic process	3/57	142/18670	0.009341	0.042428	0.028019	4513/3630/136	3
BP	GO:0006111	regulation of gluconeogenesis	2/57	48/18670	0.009441	0.042692	0.028194	3630/2033	2
BP	GO:0045912	negative regulation of	2/57	48/1867	0.00944	0.04269	0.028194	3630/2033	2

		carbohydrate metabolic process		0	1	2			
BP	GO:0071322	cellular response to carbohydrate stimulus	3/57	144/18670	0.009703	0.043779	0.028912	150/3767/5290	3
BP	GO:0035094	response to nicotine	2/57	49/18670	0.009823	0.04413	0.029144	3767/7124	2
BP	GO:0097300	programmed necrotic cell death	2/57	49/18670	0.009823	0.04413	0.029144	291/7124	2
BP	GO:0006140	regulation of nucleotide metabolic process	3/57	146/18670	0.010072	0.04515	0.029817	4513/3630/136	3
BP	GO:0014065	phosphatidylinositol 3-kinase signaling	3/57	148/18670	0.01045	0.046639	0.0308	3630/7124/5290	3
BP	GO:0051592	response to calcium ion	3/57	148/18670	0.01045	0.046639	0.0308	6331/801/6261	3
BP	GO:0043434	response to peptide hormone	5/57	436/18670	0.010503	0.046776	0.03089	5468/185/3630/88/5290	5
BP	GO:0050777	negative regulation of immune response	3/57	150/18670	0.010836	0.048153	0.0318	5468/3630/7124	3
BP	GO:0055088	lipid homeostasis	3/57	151/18670	0.011032	0.048918	0.032305	5468/3630/134	3
BP	GO:0000187	activation of MAPK activity	3/57	152/18670	0.011230	0.049582	0.032744	151/136/7124	3
BP	GO:0001678	cellular glucose homeostasis	3/57	152/18670	0.011230	0.049582	0.032744	150/3767/5290	3
CC	GO:0034703	cation channel complex	24/57	220/19717	2.10E-32	3.65E-30	2.05E-30	6337/3764/6340/6338/11280/6327/55800/6331/6336/6329/6330/6324/801/1006	24

								0/6833/775/776/3757/783/3767/779/774/6261/781	
CC	GO:0034702	ion channel complex	24/57	301/19717	4.87E-29	4.24E-27	2.39E-27	6337/3764/6340/6338/11280/6327/55800/6331/6336/6329/6330/6324/801/10060/6833/775/776/3757/783/3767/779/774/6261/781	24
CC	GO:1902495	transmembrane transporter complex	24/57	324/19717	2.94E-28	1.71E-26	9.61E-27	6337/3764/6340/6338/11280/6327/55800/6331/6336/6329/6330/6324/801/10060/6833/775/776/3757/783/3767/779/774/6261/781	24
CC	GO:1990351	transporter complex	24/57	332/19717	5.33E-28	2.32E-26	1.30E-26	6337/3764/6340/6338/11280/6327/55800/6331/6336/6329/6330/6324/801/10060/6833/775/776/3757/783/3767/779/774/6261/781	24
CC	GO:0034706	sodium channel complex	11/57	23/19717	5.56E-23	1.93E-21	1.09E-21	6337/6340/6338/11280/6327/55800/6331/6336/6329/6330/6324	11
CC	GO:0001518	voltage-gated sodium channel complex	8/57	14/19717	8.66E-18	2.51E-16	1.41E-16	11280/6327/55800/6331/6336/6329/6330/6324	8
CC	GO:0030315	T-tubule	10/57	52/19717	2.55E-16	6.35E-15	3.57E-15	148/6331/6324/775/776/783/3767/779/6261/781	10
CC	GO:00423	sarcolemma	12/57	136/	3.84	8.36	4.70E-1	148/6331/6324/	12

	83			19717	E-15	E-14	4	10060/6833/775/776/783/3767/779/6261/781	
CC	GO:0034704	calcium channel complex	8/57	66/19717	1.48E-11	2.85E-10	1.60E-10	801/775/776/783/779/774/6261/781	8
CC	GO:0005891	voltage-gated calcium channel complex	7/57	43/19717	3.43E-11	5.96E-10	3.35E-10	775/776/783/779/774/6261/781	7
CC	GO:1990454	L-type voltage-gated calcium channel complex	5/57	12/19717	1.32E-10	2.08E-09	1.17E-09	775/776/783/779/781	5
CC	GO:0030017	sarcomere	10/57	204/19717	3.19E-10	4.62E-09	2.60E-09	148/88/55800/6331/801/10060/775/776/779/6261	10
CC	GO:0044449	contractile fiber part	10/57	221/19717	6.96E-10	9.32E-09	5.24E-09	148/88/55800/6331/801/10060/775/776/779/6261	10
CC	GO:0030016	myofibril	10/57	224/19717	7.93E-10	9.86E-09	5.55E-09	148/88/55800/6331/801/10060/775/776/779/6261	10
CC	GO:0043292	contractile fiber	10/57	234/19717	1.21E-09	1.41E-08	7.91E-09	148/88/55800/6331/801/10060/775/776/779/6261	10
CC	GO:0098803	respiratory chain complex	7/57	85/19717	4.78E-09	5.20E-08	2.92E-08	1349/4512/4513/4514/6390/6391/6389	7
CC	GO:0031674	I band	8/57	143/19717	7.70E-09	7.88E-08	4.43E-08	148/88/55800/6331/775/776/779/6261	8
CC	GO:0070469	respiratory chain	7/57	100/19717	1.50E-08	1.45E-07	8.15E-08	1349/4512/4513/4514/6390/6391/6389	7
CC	GO:0030018	Z disc	7/57	132/19717	1.03E-07	9.41E-07	5.30E-07	148/88/55800/6331/775/776/6261	7
CC	GO:00452	respiratory	4/57	20/1	2.94	2.56	1.44E-0	1349/4512/4513	4

	77	chain complex IV		9717	E-07	E-06	6	/4514	
CC	GO:0070069	cytochrome complex	4/57	34/19717	2.73E-06	2.26E-05	1.27E-05	1349/4512/4513/4514	4
CC	GO:0005746	mitochondrial respiratory chain	5/57	88/19717	5.51E-06	4.36E-05	2.45E-05	1349/4512/6390/6391/6389	5
CC	GO:0034705	potassium channel complex	5/57	96/19717	8.44E-06	6.39E-05	3.59E-05	3764/10060/6833/3757/3767	5
CC	GO:0014704	intercalated disc	4/57	50/19717	1.31E-05	9.49E-05	5.34E-05	6331/6330/6324/3767	4
CC	GO:0044455	mitochondrial membrane part	6/57	229/19717	5.09E-05	0.000353	0.000199	1349/4512/291/6390/6391/6389	6
CC	GO:0044291	cell-cell contact zone	4/57	71/19717	5.28E-05	0.000353	0.000199	6331/6330/6324/3767	4
CC	GO:0099056	integral component of presynaptic membrane	4/57	73/19717	5.89E-05	0.000378	0.000213	148/6336/134/776	4
CC	GO:0005743	mitochondrial inner membrane	8/57	473/19717	6.09E-05	0.000378	0.000213	1349/4512/4513/4514/291/6390/6391/6389	8
CC	GO:0099699	integral component of synaptic membrane	5/57	152/19717	7.73E-05	0.000464	0.000261	148/152/6336/134/776	5
CC	GO:0098889	intrinsic component of presynaptic membrane	4/57	82/19717	9.28E-05	0.000538	0.000303	148/6336/134/776	4
CC	GO:0099240	intrinsic component of synaptic membrane	5/57	164/19717	0.000111	0.000621	0.000349	148/152/6336/134/776	5
CC	GO:0097060	synaptic membrane	7/57	432/19717	0.000237	0.00129	0.000726	148/152/88/6336/134/775/776	7
CC	GO:1990204	oxidoreductase complex	4/57	112/197	0.00030	0.00162	0.000917	4512/6390/6391/6389	4

				17	9	9			
CC	GO:0097470	ribbon synapse	2/57	10/19717	0.000364	0.001863	0.001048	776/783	2
CC	GO:0098793	presynapse	7/57	491/19717	0.000513	0.002549	0.001434	148/152/6336/134/776/783/774	7
CC	GO:0098800	inner mitochondrial membrane protein complex	4/57	135/19717	0.000627	0.00303	0.001705	4512/6390/6391/6389	4
CC	GO:0030673	axolemma	2/57	14/19717	0.000731	0.003436	0.001933	134/3767	2
CC	GO:0044304	main axon	3/57	68/19717	0.001005	0.004601	0.002589	6324/134/3767	3
CC	GO:0042734	presynaptic membrane	4/57	161/19717	0.001208	0.00539	0.003033	148/6336/134/776	4
CC	GO:0005901	caveola	3/57	80/19717	0.001607	0.006992	0.003934	148/147/6331	3
CC	GO:0045211	postsynaptic membrane	5/57	323/19717	0.002386	0.010125	0.005697	148/152/88/134/775	5
CC	GO:0043025	neuronal cell body	6/57	497/19717	0.003043	0.012608	0.007094	152/6324/134/775/3767/774	6
CC	GO:0098978	glutamatergic synapse	5/57	349/19717	0.003328	0.013465	0.007576	148/152/88/6336/136	5
CC	GO:0044853	plasma membrane raft	3/57	109/19717	0.003871	0.01531	0.008614	148/147/6331	3
CC	GO:0099055	integral component of postsynaptic membrane	3/57	117/19717	0.00472	0.01825	0.010268	148/152/134	3
CC	GO:0098936	intrinsic component of postsynaptic membrane	3/57	122/19717	0.005303	0.020058	0.011285	148/152/134	3

CC	GO:0098798	mitochondrial protein complex	4/57	262/19717	0.006934	0.025669	0.014442	4512/6390/6391/6389	4
CC	GO:0032589	neuron projection membrane	2/57	57/19717	0.011836	0.042906	0.024139	134/3767	2
CC	GO:0045121	membrane raft	4/57	315/19717	0.012999	0.045721	0.025723	148/147/6331/7124	4
CC	GO:0098857	membrane microdomain	4/57	316/19717	0.013138	0.045721	0.025723	148/147/6331/7124	4
CC	GO:0016324	apical plasma membrane	4/57	318/19717	0.013419	0.045781	0.025757	6337/6340/154/6338	4
CC	GO:0014069	postsynaptic density	4/57	324/19717	0.014283	0.047028	0.026458	152/88/134/775	4
CC	GO:0022625	cytosolic large ribosomal subunit	2/57	63/19717	0.014325	0.047028	0.026458	6135/6138	2
CC	GO:0032279	asymmetric synapse	4/57	328/19717	0.014879	0.047071	0.026483	152/88/134/775	4
CC	GO:0098589	membrane region	4/57	328/19717	0.014879	0.047071	0.026483	148/147/6331/7124	4
MF	GO:0005261	cation channel activity	23/57	319/17697	1.06E-25	2.51E-23	1.63E-23	6337/3764/6340/6338/11280/6327/55800/6331/6336/6329/6330/6324/10060/6833/775/776/3757/783/3767/779/774/6261/781	23
MF	GO:0005216	ion channel activity	23/57	416/17697	4.80E-23	5.69E-21	3.69E-21	6337/3764/6340/6338/11280/6327/55800/6331/6336/6329/6330/6324/10060/6833/775/776/3757/783/3767/779/774/6261/781	23
MF	GO:00228	substrate-speci	23/57	428/	9.19	7.26	4.71E-2	6337/3764/6340	23

	38	fic channel activity		176 97	E-23	E-21	1	/6338/11280/63 27/55800/6331/ 6336/6329/6330 /6324/10060/68 33/775/776/375 7/783/3767/779/ 774/6261/781	
MF	GO:00468 73	metal ion transmembran e transporter activity	23/57	438/ 176 97	1.56 E-22	9.22 E-21	5.98E-2 1	6337/3764/6340 /6338/11280/63 27/55800/6331/ 6336/6329/6330 /6324/10060/68 33/775/776/375 7/783/3767/779/ 774/6261/781	23
MF	GO:00228 39	ion gated channel activity	21/57	334/ 176 97	3.81 E-22	1.38 E-20	8.97E-2 1	6337/3764/6340 /6338/11280/63 27/55800/6331/ 6336/6329/6330 /6324/775/776/3 757/783/3767/7 79/774/6261/78 1	21
MF	GO:00152 67	channel activity	23/57	456/ 176 97	3.89 E-22	1.38 E-20	8.97E-2 1	6337/3764/6340 /6338/11280/63 27/55800/6331/ 6336/6329/6330 /6324/10060/68 33/775/776/375 7/783/3767/779/ 774/6261/781	23
MF	GO:00228 03	passive transmembran e transporter activity	23/57	457/ 176 97	4.09 E-22	1.38 E-20	8.97E-2 1	6337/3764/6340 /6338/11280/63 27/55800/6331/ 6336/6329/6330 /6324/10060/68 33/775/776/375 7/783/3767/779/ 774/6261/781	23
MF	GO:00052 44	voltage-gated ion channel activity	18/57	197/ 176 97	6.65 E-22	1.58 E-20	1.02E-2 0	3764/11280/632 7/55800/6331/6 336/6329/6330/ 6324/775/776/3 757/783/3767/7	18

								79/774/6261/781	
MF	GO:0022832	voltage-gated channel activity	18/57	197/17697	6.65E-22	1.58E-20	1.02E-20	3764/11280/6327/55800/6331/6336/6329/6330/6324/775/776/3757/783/3767/779/774/6261/781	18
MF	GO:0022836	gated channel activity	21/57	343/17697	6.66E-22	1.58E-20	1.02E-20	6337/3764/6340/6338/11280/6327/55800/6331/6336/6329/6330/6324/775/776/3757/783/3767/779/774/6261/781	21
MF	GO:0015077	monovalent inorganic cation transmembrane transporter activity	20/57	382/17697	1.72E-19	3.70E-18	2.40E-18	1349/4512/4513/4514/6337/3764/6340/6338/11280/6327/55800/6331/6336/6329/6330/6324/10060/6833/3757/3767	20
MF	GO:0005272	sodium channel activity	11/57	41/17697	4.07E-19	8.05E-18	5.22E-18	6337/6340/6338/11280/6327/55800/6331/6336/6329/6330/6324	11
MF	GO:0004935	adrenergic receptor activity	8/57	17/17697	1.65E-16	3.01E-15	1.95E-15	148/154/150/153/146/147/152/151	8
MF	GO:0005248	voltage-gated sodium channel activity	8/57	20/17697	8.48E-16	1.44E-14	9.31E-15	11280/6327/55800/6331/6336/6329/6330/6324	8
MF	GO:0008227	G protein-coupled amine receptor activity	9/57	59/17697	2.14E-13	3.38E-12	2.19E-12	148/154/150/153/146/147/152/151/3360	9
MF	GO:0015081	sodium ion transmembrane transporter	11/57	149/17697	1.37E-12	2.03E-11	1.32E-11	6337/6340/6338/11280/6327/55800/6331/6336/	11

		activity						6329/6330/6324	
MF	GO:0044325	ion channel binding	10/57	124/17697	6.45E-12	9.00E-11	5.84E-11	88/55800/6331/6336/6330/6324/801/10060/6833/3767	10
MF	GO:0022843	voltage-gated cation channel activity	10/57	142/17697	2.52E-11	3.31E-10	2.15E-10	3764/775/776/3757/783/3767/779/774/6261/781	10
MF	GO:0008331	high voltage-gated calcium channel activity	5/57	10/17697	7.21E-11	8.99E-10	5.83E-10	775/776/783/779/774	5
MF	GO:0005245	voltage-gated calcium channel activity	7/57	48/17697	1.63E-10	1.93E-09	1.25E-09	775/776/783/779/774/6261/781	7
MF	GO:1901338	catecholamine binding	5/57	20/17697	4.33E-09	4.88E-08	3.17E-08	154/150/153/152/151	5
MF	GO:0099094	ligand-gated cation channel activity	7/57	105/17697	4.38E-08	4.72E-07	3.06E-07	6337/3764/6340/6338/3757/3767/6261	7
MF	GO:0009055	electron transfer activity	7/57	114/17697	7.75E-08	7.98E-07	5.18E-07	1349/4512/4513/4514/6390/6391/6389	7
MF	GO:0005262	calcium channel activity	7/57	123/17697	1.31E-07	1.29E-06	8.37E-07	775/776/783/779/774/6261/781	7
MF	GO:0015276	ligand-gated ion channel activity	7/57	138/17697	2.87E-07	2.62E-06	1.70E-06	6337/3764/6340/6338/3757/3767/6261	7
MF	GO:0022834	ligand-gated channel activity	7/57	138/17697	2.87E-07	2.62E-06	1.70E-06	6337/3764/6340/6338/3757/3767/6261	7
MF	GO:0015085	calcium ion transmembrane transporter activity	7/57	139/17697	3.02E-07	2.65E-06	1.72E-06	775/776/783/779/774/6261/781	7
MF	GO:0016247	channel regulator activity	7/57	144/17697	3.84E-07	3.25E-06	2.11E-06	154/6327/55800/6330/6324/801/10060	7
MF	GO:00041	cytochrome-c	4/57	28/1	1.87	1.43	9.27E-0	1349/4512/4513	4

	29	oxidase activity		7697	E-06	E-05	6	/4514	
MF	GO:0015002	heme-copper terminal oxidase activity	4/57	28/17697	1.87E-06	1.43E-05	9.27E-06	1349/4512/4513/4514	4
MF	GO:0016676	oxidoreductase activity, acting on a heme group of donors, oxygen as acceptor	4/57	28/17697	1.87E-06	1.43E-05	9.27E-06	1349/4512/4513/4514	4
MF	GO:0016675	oxidoreductase activity, acting on a heme group of donors	4/57	29/17697	2.16E-06	1.60E-05	1.04E-05	1349/4512/4513/4514	4
MF	GO:0015280	ligand-gated sodium channel activity	3/57	10/17697	3.74E-06	2.69E-05	1.74E-05	6337/6340/6338	3
MF	GO:0017080	sodium channel regulator activity	4/57	36/17697	5.27E-06	3.68E-05	2.38E-05	6327/55800/6330/6324	4
MF	GO:0031690	adrenergic receptor binding	3/57	19/17697	2.96E-05	0.0002	0.00013	150/153/152	3
MF	GO:0005242	inward rectifier potassium channel activity	3/57	20/17697	3.47E-05	0.000223	0.000144	3764/3757/3767	3
MF	GO:0030506	ankyrin binding	3/57	20/17697	3.47E-05	0.000223	0.000144	6331/776/3767	3
MF	GO:0099106	ion channel regulator activity	5/57	118/17697	3.84E-05	0.00024	0.000155	154/55800/6324/801/10060	5
MF	GO:0005267	potassium channel activity	5/57	123/17697	4.69E-05	0.000285	0.000185	3764/10060/6833/3757/3767	5
MF	GO:00506	WW domain	3/57	31/1	0.00	0.00	0.00051	6337/6340/6338	3

	99	binding		7697	0134	0791	3		
MF	GO:0015079	potassium ion transmembrane transporter activity	5/57	159/17697	0.000158	0.000913	0.000592	3764/10060/6833/3757/3767	5
MF	GO:0008200	ion channel inhibitor activity	3/57	37/17697	0.000228	0.001255	0.000814	55800/6324/801	3
MF	GO:0051393	alpha-actinin binding	3/57	37/17697	0.000228	0.001255	0.000814	5468/775/776	3
MF	GO:0016248	channel inhibitor activity	3/57	38/17697	0.000247	0.001329	0.000862	55800/6324/801	3
MF	GO:0042805	actinin binding	3/57	46/17697	0.000436	0.002296	0.001489	5468/775/776	3
MF	GO:1902282	voltage-gated potassium channel activity involved in ventricular cardiac muscle cell action potential repolarization	2/57	12/17697	0.000659	0.003395	0.002201	3764/3757	2
MF	GO:0008179	adenylate cyclase binding	2/57	13/17697	0.000777	0.003919	0.002541	154/801	2
MF	GO:0016627	oxidoreductase activity, acting on the CH-CH group of donors	3/57	58/17697	0.000862	0.004257	0.00276	6390/6391/6389	3
MF	GO:0015078	proton transmembrane transporter activity	4/57	133/17697	0.000885	0.00428	0.002775	1349/4512/4513/4514	4
MF	GO:0031432	titin binding	2/57	14/17697	0.000905	0.004289	0.002781	88/801	2
MF	GO:00860	voltage-gated	2/57	15/1	0.00	0.00	0.00313	3764/3757	2

	08	potassium channel activity involved in cardiac muscle cell action potential repolarization		7697	1042	4841	9		
MF	GO:0001664	G protein-coupled receptor binding	5/57	280/17697	0.002047	0.009328	0.006049	150/153/152/185/134	5
MF	GO:0005249	voltage-gated potassium channel activity	3/57	87/17697	0.002773	0.012398	0.008039	3764/3757/3767	3
MF	GO:0035586	purinergic receptor activity	2/57	25/17697	0.002916	0.012797	0.008298	134/136	2
MF	GO:0008022	protein C-terminus binding	4/57	187/17697	0.003082	0.01328	0.008611	5468/3767/774/2033	4
MF	GO:0005516	calmodulin binding	4/57	200/17697	0.003918	0.016583	0.010753	6331/775/779/6261	4
MF	GO:0030594	neurotransmitter receptor activity	3/57	117/17697	0.006357	0.026432	0.01714	153/3360/134	3
MF	GO:0015399	primary active transmembrane transporter activity	3/57	125/17697	0.007626	0.030975	0.020086	4514/10060/6833	3
MF	GO:0099528	G protein-coupled neurotransmitter receptor activity	2/57	41/17697	0.007711	0.030975	0.020086	153/3360	2
MF	GO:0002020	protease binding	3/57	128/17697	0.008138	0.032144	0.020844	3630/7124/6261	3
MF	GO:0015459	potassium channel regulator	2/57	52/17697	0.012191	0.047364	0.030714	154/10060	2

	activity

Table S10 KEGG pathway enrichment

ID	Description	GeneRatio	BgRatio	pvalue	p.adjust	qvalue	geneID	Count
hsa04022	cGMP-PKG signaling pathway	18/50	167/8073	1.94E-18	3.75E-16	2.72E-16	4879/3764/148/154/150/153/146/147/152/185/151/3630/801/134/291/775/776/779	18
hsa04261	Adrenergic signaling in cardiomyocytes	15/50	150/8073	6.90E-15	6.66E-13	4.83E-13	148/154/153/146/147/185/6331/6330/6324/801/775/776/783/779/781	15
hsa04020	Calcium signaling pathway	15/50	201/8073	5.43E-13	3.50E-11	2.54E-11	148/154/153/146/147/185/3360/801/136/291/775/776/779/774/6261	15
hsa05020	Prion disease	15/50	273/8073	4.59E-11	2.21E-09	1.61E-09	1349/4512/4513/4514/291/775/776/779/774/7124/6261/5290/6390/6391/6389	15
hsa04930	Type II diabetes mellitus	8/50	46/8073	2.63E-10	1.02E-08	7.37E-09	3630/6833/775/776/3767/774/7124/5290	8
hsa04260	Cardiac muscle contraction	9/50	87/8073	2.25E-09	7.23E-08	5.24E-08	1349/4512/4513/4514/775/776/783/779/781	9
hsa05010	Alzheimer disease	15/50	369/8073	3.12E-09	8.60E-08	6.24E-08	1349/4512/4513/4514/3630/801/291/775/776/779/7124/5290/6390/6391/6389	15
hsa04270	Vascular smooth muscle contraction	10/50	135/8073	7.12E-09	1.63E-07	1.18E-07	4879/148/146/147/185/801/136/775/776/779	10
hsa04924	Renin secretion	8/50	69/8073	7.58E-09	1.63E-07	1.18E-07	154/153/185/801/134/775/776/779	8
hsa04932	Non-alcoholic fatty liver disease	10/50	150/8073	1.97E-08	3.81E-07	2.76E-07	1349/4512/4513/4514/3630/7124/5290/6390/6391/6389	10
hsa04080	Neuroactive ligand-recepto	13/50	341/80	9.34E-	1.64E-06	1.19E-06	148/154/150/153/146/147/152/185/151/336	13

	r interaction		73	08			0/134/136/5340	
hsa04024	cAMP signaling pathway	10/50	216/8073	6.15E-07	9.89E-06	7.18E-06	154/153/3360/801/134/775/776/779/5290/2033	10
hsa05414	Dilated cardiomyopathy	7/50	96/8073	1.77E-06	2.63E-05	1.91E-05	153/775/776/783/779/7124/781	7
hsa05016	Huntington disease	11/50	306/8073	1.93E-06	2.66E-05	1.93E-05	1349/4512/4513/4514/5468/291/774/6390/6391/6389/2033	11
hsa04960	Aldosterone-regulated sodium reabsorption	5/50	37/8073	2.79E-06	3.59E-05	2.60E-05	6337/6340/6338/3630/5290	5
hsa05412	Arrhythmogenic right ventricular cardiomyopathy	6/50	77/8073	7.04E-06	8.49E-05	6.16E-05	88/775/776/783/779/781	6
hsa04714	Thermogenesis	9/50	231/8073	9.91E-06	0.000113	8.16E-05	1349/4512/4513/4514/4879/5468/6390/6391/6389	9
hsa04911	Insulin secretion	6/50	86/8073	1.34E-05	0.000143	0.000104	3630/6833/775/776/3767/779	6
hsa00190	Oxidative phosphorylation	7/50	133/8073	1.56E-05	0.000158	0.000115	1349/4512/4513/4514/6390/6391/6389	7
hsa05410	Hypertrophic cardiomyopathy	6/50	90/8073	1.74E-05	0.000167	0.000121	775/776/783/779/7124/781	6
hsa05012	Parkinson disease	9/50	249/8073	1.81E-05	0.000167	0.000121	1349/4512/4513/4514/801/291/6390/6391/6389	9
hsa04970	Salivary secretion	6/50	93/8073	2.10E-05	0.000184	0.000134	148/154/153/146/147/801	6
hsa04923	Regulation of lipolysis in adipocytes	5/50	56/8073	2.24E-05	0.000188	0.000136	154/153/3630/134/5290	5
hsa04921	Oxytocin signaling pathway	7/50	154/8073	4.03E-05	0.000324	0.000235	801/775/776/783/779/6261/781	7
hsa04929	GnRH	5/50	64/	4.3	0.000	0.000	775/776/3767/779/52	5

	secretion		8073	0E-05	332	241	90	
hsa04925	Aldosterone synthesis and secretion	5/50	98/8073	0.000327	0.002428	0.001761	185/801/775/776/779	5
hsa04010	MAPK signaling pathway	8/50	294/8073	0.000398	0.002845	0.002064	3630/775/776/783/779/774/7124/781	8
hsa04725	Cholinergic synapse	5/50	113/8073	0.000631	0.004347	0.003153	775/776/779/774/5290	5
hsa04927	Cortisol synthesis and secretion	4/50	65/8073	0.000668	0.004395	0.003188	185/775/776/779	4
hsa04726	Serotonergic synapse	5/50	115/8073	0.000683	0.004395	0.003188	3360/775/776/779/774	5
hsa04935	Growth hormone synthesis, secretion and action	5/50	119/8073	0.000798	0.004866	0.00353	775/776/779/5290/2033	5
hsa00020	Citrate cycle (TCA cycle)	3/50	30/8073	0.000807	0.004866	0.00353	6390/6391/6389	3
hsa05014	Amyotrophic lateral sclerosis	8/50	364/8073	0.001611	0.009421	0.006834	1349/4512/4513/4514/7124/6390/6391/6389	8
hsa04742	Taste transduction	4/50	86/8073	0.001904	0.010806	0.007839	6337/6340/6338/775	4
hsa04727	GABAergic synapse	4/50	89/8073	0.002159	0.011907	0.008637	775/776/779/774	4
hsa04912	GnRH signaling pathway	4/50	93/8073	0.002536	0.013594	0.009861	801/775/776/779	4
hsa04713	Circadian entrainment	4/50	97/8073	0.002955	0.015414	0.011181	801/775/776/6261	4
hsa05164	Influenza A	5/50	171/8073	0.003944	0.020033	0.014532	291/7124/5290/5340/2033	5
hsa04152	AMPK	4/50	120	0.0	0.031	0.022	5468/148/3630/5290	4

	signaling pathway		/8073	06314	245	665		
hsa04720	Long-term potentiation	3/50	67/8073	0.008109	0.039124	0.02838	801/775/2033	3
hsa05031	Amphetamine addiction	3/50	69/8073	0.008792	0.040426	0.029325	801/775/776	3
hsa04728	Dopaminergic synapse	4/50	132/8073	0.008797	0.040426	0.029325	801/775/776/774	4
hsa04723	Retrograde endocannabinoid signaling	4/50	148/8073	0.013001	0.058353	0.042329	775/776/779/774	4
hsa04934	Cushing syndrome	4/50	155/8073	0.015184	0.066537	0.048265	185/775/776/779	4
hsa04218	Cellular senescence	4/50	156/8073	0.015514	0.066537	0.048265	801/291/776/5290	4
hsa04211	Longevity regulating pathway	3/50	89/8073	0.017484	0.073355	0.053211	5468/3630/5290	3
hsa05215	Prostate cancer	3/50	97/8073	0.021932	0.090063	0.065331	3630/5290/2033	3
hsa04933	AGE-RAGE signaling pathway in diabetic complications	3/50	100/8073	0.023746	0.095479	0.06926	185/7124/5290	3
hsa04625	C-type lectin receptor signaling pathway	3/50	104/8073	0.026288	0.103543	0.075109	801/7124/5290	3
hsa04940	Type I diabetes mellitus	2/50	43/8073	0.028886	0.109636	0.079529	3630/7124	2
hsa04931	Insulin resistance	3/50	108/8073	0.028971	0.109636	0.079529	3630/7124/5290	3
hsa04066	HIF-1 signaling pathway	3/50	109/8073	0.029664	0.110099	0.079864	3630/5290/2033	3

hsa02010	ABC transporters	2/50	45/8073	0.031423	0.114425	0.083003	10060/6833	2
hsa04973	Carbohydrate digestion and absorption	2/50	47/8073	0.034044	0.121677	0.088263	776/5290	2
hsa01200	Carbon metabolism	3/50	118/8073	0.036293	0.127354	0.092381	6390/6391/6389	3
hsa04071	Sphingolipid signaling pathway	3/50	119/8073	0.037073	0.127768	0.092681	134/7124/5290	3
hsa04015	Rap1 signaling pathway	4/50	210/8073	0.040404	0.136805	0.099237	3630/801/136/5290	4
hsa04380	Osteoclast differentiation	3/50	128/8073	0.04448	0.14801	0.107365	5468/7124/5290	3
hsa05166	Human T-cell leukemia virus 1 infection	4/50	219/8073	0.045963	0.150354	0.109065	291/7124/5290/2033	4
hsa04068	FoxO signaling pathway	3/50	131/8073	0.047102	0.15151	0.109904	3630/5290/2033	3
hsa04371	Apelin signaling pathway	3/50	137/8073	0.052571	0.163649	0.118709	185/801/6261	3
hsa04910	Insulin signaling pathway	3/50	137/8073	0.052571	0.163649	0.118709	3630/801/5290	3
hsa05418	Fluid shear stress and atherosclerosis	3/50	139/8073	0.05446	0.166839	0.121023	801/7124/5290	3
hsa04213	Longevity regulating pathway - multiple species	2/50	62/8073	0.056182	0.169423	0.122898	3630/5290	2
hsa05017	Spinocerebellar ataxia	3/50	143/8073	0.058336	0.173213	0.125647	291/6261/5290	3
hsa04072	Phospholipase	3/50	148	0.0	0.185	0.134	185/3630/5290	3

	D signaling pathway		/8073	63361	283	402		
hsa04664	Fc epsilon RI signaling pathway	2/50	68/8073	0.066127	0.190486	0.138176	7124/5290	2
hsa05211	Renal cell carcinoma	2/50	69/8073	0.067839	0.192542	0.139668	5290/2033	2
hsa04917	Prolactin signaling pathway	2/50	70/8073	0.069565	0.19458	0.141146	3630/5290	2
hsa04150	mTOR signaling pathway	3/50	155/8073	0.070724	0.194997	0.141449	3630/7124/5290	3
hsa05214	Glioma	2/50	75/8073	0.078409	0.210315	0.15256	801/5290	2
hsa05161	Hepatitis B	3/50	162/8073	0.078459	0.210315	0.15256	7124/5290/2033	3
hsa05133	Pertussis	2/50	76/8073	0.080219	0.212086	0.153845	801/7124	2
hsa05152	Tuberculosis	3/50	180/8073	0.099959	0.260704	0.189112	801/7124/2033	3
hsa05032	Morphine addiction	2/50	91/8073	0.10882	0.280029	0.20313	134/774	2
hsa04350	TGF-beta signaling pathway	2/50	94/8073	0.114831	0.29161	0.211531	7124/2033	2
hsa05167	Kaposi sarcoma-associated herpesvirus infection	3/50	193/8073	0.116817	0.292801	0.212395	801/5290/2033	3
hsa04070	Phosphatidylinositol signaling system	2/50	97/8073	0.120927	0.299217	0.217049	801/5290	2
hsa04750	Inflammatory mediator regulation of	2/50	100/8073	0.127103	0.303981	0.220504	801/5290	2

	TRP channels							
hsa04914	Progesterone-mediated oocyte maturation	2/50	100/8073	0.127103	0.303981	0.220504	3630/5290	2
hsa04916	Melanogenesis	2/50	101/8073	0.129178	0.303981	0.220504	801/2033	2
hsa05142	Chagas disease	2/50	102/8073	0.131262	0.303981	0.220504	7124/5290	2
hsa05146	Amoebiasis	2/50	102/8073	0.131262	0.303981	0.220504	7124/5290	2
hsa04614	Renin-angiotensin system	1/50	23/8073	0.133323	0.303981	0.220504	185	1
hsa04620	Toll-like receptor signaling pathway	2/50	104/8073	0.135453	0.303981	0.220504	7124/5290	2
hsa04660	T cell receptor signaling pathway	2/50	104/8073	0.135453	0.303981	0.220504	7124/5290	2
hsa04977	Vitamin digestion and absorption	1/50	24/8073	0.138706	0.307704	0.223205	10560	1
hsa04922	Glucagon signaling pathway	2/50	107/8073	0.141796	0.310595	0.225302	801/2033	2
hsa05170	Human immunodeficiency virus 1 infection	3/50	212/8073	0.143228	0.310595	0.225302	801/7124/5290	3
hsa04950	Maturity onset diabetes of the young	1/50	26/8073	0.149374	0.320324	0.23236	3630	1
hsa04668	TNF signaling pathway	2/50	112/8073	0.152507	0.32345	0.234627	7124/5290	2
hsa04724	Glutamatergic synapse	2/50	114/8073	0.156837	0.329017	0.238665	775/776	2
hsa04744	Phototransduc	1/50	28/	0.1	0.331	0.240	801	1

	tion		8073	59913	862	729		
hsa05163	Human cytomegalovirus infection	3/50	225/8073	0.162362	0.33336	0.241815	801/7124/5290	3
hsa04722	Neurotrophin signaling pathway	2/50	119/8073	0.167764	0.340826	0.247232	801/5290	2
hsa04919	Thyroid hormone signaling pathway	2/50	121/8073	0.172173	0.342099	0.248155	5290/2033	2
hsa04014	Ras signaling pathway	3/50	232/8073	0.172979	0.342099	0.248155	3630/801/5290	3
hsa01523	Antifolate resistance	1/50	31/8073	0.175481	0.342099	0.248155	7124	1
hsa05310	Asthma	1/50	31/8073	0.175481	0.342099	0.248155	7124	1
hsa04114	Oocyte meiosis	2/50	129/8073	0.189994	0.366689	0.265992	3630/801	2
hsa05135	Yersinia infection	2/50	130/8073	0.192241	0.367351	0.266473	7124/5290	2
hsa04650	Natural killer cell mediated cytotoxicity	2/50	131/8073	0.194491	0.368008	0.266949	7124/5290	2
hsa05143	African trypanosomiasis	1/50	37/8073	0.205774	0.376557	0.27315	7124	1
hsa05216	Thyroid cancer	1/50	37/8073	0.205774	0.376557	0.27315	5468	1
hsa04210	Apoptosis	2/50	136/8073	0.205794	0.376557	0.27315	7124/5290	2
hsa04140	Autophagy - animal	2/50	137/8073	0.208064	0.376557	0.27315	3630/5290	2
hsa04915	Estrogen signaling	2/50	138/80	0.2103	0.376557	0.27315	801/5290	2

	pathway		73	37				
hsa05330	Allograft rejection	1/50	38/8073	0.210716	0.376557	0.27315	7124	1
hsa05033	Nicotine addiction	1/50	40/8073	0.220509	0.390442	0.283222	774	1
hsa05332	Graft-versus-host disease	1/50	42/8073	0.230183	0.403866	0.29296	7124	1
hsa05160	Hepatitis C	2/50	157/8073	0.253913	0.441488	0.32025	7124/5290	2
hsa03010	Ribosome	2/50	158/8073	0.256219	0.441521	0.320274	6135/6138	2
hsa04217	Necroptosis	2/50	159/8073	0.258526	0.441554	0.320298	291/7124	2
hsa04630	JAK-STAT signaling pathway	2/50	162/8073	0.265451	0.449297	0.325915	5290/2033	2
hsa05144	Malaria	1/50	50/8073	0.267716	0.449297	0.325915	7124	1
hsa04913	Ovarian steroidogenesis	1/50	51/8073	0.27228	0.453017	0.328613	3630	1
hsa04330	Notch signaling pathway	1/50	53/8073	0.281323	0.464063	0.336626	2033	1
hsa00240	Pyrimidine metabolism	1/50	57/8073	0.299082	0.481023	0.348929	50484	1
hsa00480	Glutathione metabolism	1/50	57/8073	0.299082	0.481023	0.348929	50484	1
hsa05134	Legionellosis	1/50	57/8073	0.299082	0.481023	0.348929	7124	1
hsa05213	Endometrial cancer	1/50	58/8073	0.303454	0.484022	0.351104	5290	1
hsa04370	VEGF	1/50	59/	0.3	0.486	0.353	5290	1

	signaling pathway		8073	07799	928	212		
hsa04730	Long-term depression	1/50	60/8073	0.312118	0.489746	0.355256	6261	1
hsa05034	Alcoholism	2/50	187/8073	0.323026	0.497622	0.360969	801/136	2
hsa05321	Inflammatory bowel disease	1/50	65/8073	0.333318	0.497622	0.360969	7124	1
hsa05165	Human papillomavirus infection	3/50	331/8073	0.337121	0.497622	0.360969	7124/5290/2033	3
hsa05221	Acute myeloid leukemia	1/50	67/8073	0.341618	0.497622	0.360969	5290	1
hsa05223	Non-small cell lung cancer	1/50	68/8073	0.34573	0.497622	0.360969	5290	1
hsa04920	Adipocytokine signaling pathway	1/50	69/8073	0.349817	0.497622	0.360969	7124	1
hsa04622	RIG-I-like receptor signaling pathway	1/50	70/8073	0.353878	0.497622	0.360969	7124	1
hsa05230	Central carbon metabolism in cancer	1/50	70/8073	0.353878	0.497622	0.360969	5290	1
hsa05169	Epstein-Barr virus infection	2/50	202/8073	0.357137	0.497622	0.360969	7124/5290	2
hsa04520	Adherens junction	1/50	71/8073	0.357915	0.497622	0.360969	2033	1
hsa05203	Viral carcinogenesis	2/50	204/8073	0.361645	0.497622	0.360969	5290/2033	2
hsa05218	Melanoma	1/50	72/8073	0.361927	0.497622	0.360969	5290	1
hsa05205	Proteoglycans	2/50	205	0.3	0.497	0.360	7124/5290	2

	in cancer		/8073	63895	622	969		
hsa00562	Inositol phosphate metabolism	1/50	73/8073	0.365914	0.497622	0.360969	5290	1
hsa01524	Platinum drug resistance	1/50	73/8073	0.365914	0.497622	0.360969	5290	1
hsa04115	p53 signaling pathway	1/50	73/8073	0.365914	0.497622	0.360969	50484	1
hsa05100	Bacterial invasion of epithelial cells	1/50	73/8073	0.365914	0.497622	0.360969	5290	1
hsa04971	Gastric acid secretion	1/50	76/8073	0.377731	0.497622	0.360969	801	1
hsa05212	Pancreatic cancer	1/50	76/8073	0.377731	0.497622	0.360969	5290	1
hsa05220	Chronic myeloid leukemia	1/50	76/8073	0.377731	0.497622	0.360969	5290	1
hsa03320	PPAR signaling pathway	1/50	77/8073	0.381621	0.497622	0.360969	5468	1
hsa05140	Leishmaniasis	1/50	77/8073	0.381621	0.497622	0.360969	7124	1
hsa05132	Salmonella infection	2/50	213/8073	0.381791	0.497622	0.360969	7124/5290	2
hsa04810	Regulation of actin cytoskeleton	2/50	214/8073	0.384015	0.497622	0.360969	3630/5290	2
hsa04612	Antigen processing and presentation	1/50	78/8073	0.385488	0.497622	0.360969	7124	1
hsa04721	Synaptic vesicle cycle	1/50	78/8073	0.385488	0.497622	0.360969	774	1
hsa00983	Drug metabolism -	1/50	79/807	0.3893	0.497622	0.360969	50484	1

	other enzymes		3	31				
hsa01521	EGFR tyrosine kinase inhibitor resistance	1/50	79/8073	0.389331	0.497622	0.360969	5290	1
hsa04662	B cell receptor signaling pathway	1/50	82/8073	0.40072	0.508809	0.369084	5290	1
hsa04012	ErbB signaling pathway	1/50	85/8073	0.4119	0.516213	0.374455	5290	1
hsa04610	Complement and coagulation cascades	1/50	85/8073	0.4119	0.516213	0.374455	5340	1
hsa05210	Colorectal cancer	1/50	86/8073	0.415581	0.517466	0.375364	5290	1
hsa04540	Gap junction	1/50	88/8073	0.422876	0.523174	0.379504	153	1
hsa05235	PD-L1 expression and PD-1 checkpoint pathway in cancer	1/50	89/8073	0.42649	0.524284	0.380309	5290	1
hsa05222	Small cell lung cancer	1/50	92/8073	0.437199	0.529934	0.384408	5290	1
hsa04666	Fc gamma R-mediated phagocytosis	1/50	93/8073	0.440725	0.529934	0.384408	5290	1
hsa05323	Rheumatoid arthritis	1/50	93/8073	0.440725	0.529934	0.384408	7124	1
hsa04657	IL-17 signaling pathway	1/50	94/8073	0.444229	0.529934	0.384408	7124	1
hsa05131	Shigellosis	2/50	242/8073	0.444815	0.529934	0.384408	7124/5290	2

hsa05150	Staphylococcus aureus infection	1/50	96/8073	0.451173	0.534211	0.38751	5340	1
hsa01522	Endocrine resistance	1/50	98/8073	0.458032	0.535758	0.388633	5290	1
hsa05231	Choline metabolism in cancer	1/50	98/8073	0.458032	0.535758	0.388633	5290	1
hsa04640	Hematopoietic cell lineage	1/50	99/8073	0.46143	0.536482	0.389158	7124	1
hsa04061	Viral protein interaction with cytokine and cytokine receptor	1/50	100/8073	0.464807	0.537172	0.389658	7124	1
hsa04064	NF-kappa B signaling pathway	1/50	104/8073	0.478109	0.549256	0.398424	7124	1
hsa05145	Toxoplasmosis	1/50	112/8073	0.503748	0.575286	0.417306	7124	1
hsa04670	Leukocyte transendothelial migration	1/50	114/8073	0.509962	0.578957	0.419969	5290	1
hsa04110	Cell cycle	1/50	124/8073	0.539908	0.605827	0.43946	2033	1
hsa04611	Platelet activation	1/50	124/8073	0.539908	0.605827	0.43946	5290	1
hsa04926	Relaxin signaling pathway	1/50	129/8073	0.5542	0.617829	0.448166	5290	1
hsa00230	Purine metabolism	1/50	130/8073	0.557006	0.617829	0.448166	50484	1
hsa05322	Systemic lupus erythematosus	1/50	136/8073	0.573482	0.632469	0.458785	7124	1
hsa05206	MicroRNAs in cancer	2/50	310/8073	0.57776	0.633566	0.459582	5290/2033	2

hsa05162	Measles	1/50	139/8073	0.581493	0.634057	0.459938	5290	1
hsa04550	Signaling pathways regulating pluripotency of stem cells	1/50	143/8073	0.591945	0.641828	0.465575	5290	1
hsa05224	Breast cancer	1/50	147/8073	0.602142	0.649237	0.470949	5290	1
hsa05226	Gastric cancer	1/50	149/8073	0.607146	0.650995	0.472224	5290	1
hsa04310	Wnt signaling pathway	1/50	160/8073	0.633586	0.675591	0.490066	2033	1
hsa04530	Tight junction	1/50	162/8073	0.638202	0.676774	0.490924	776	1
hsa04151	PI3K-Akt signaling pathway	2/50	354/8073	0.651025	0.683588	0.495867	3630/5290	2
hsa05225	Hepatocellular carcinoma	1/50	168/8073	0.651711	0.683588	0.495867	5290	1
hsa04360	Axon guidance	1/50	181/8073	0.679308	0.704873	0.511307	5290	1
hsa04621	NOD-like receptor signaling pathway	1/50	181/8073	0.679308	0.704873	0.511307	7124	1
hsa04062	Chemokine signaling pathway	1/50	192/8073	0.700976	0.717748	0.520646	5290	1
hsa05202	Transcriptional misregulation in cancer	1/50	192/8073	0.700976	0.717748	0.520646	5468	1
hsa05130	Pathogenic Escherichia coli infection	1/50	193/8073	0.702873	0.717748	0.520646	7124	1
hsa04510	Focal	1/50	201	0.7	0.728	0.528	5290	1

	adhesion		/8073	17631	962	781		
hsa05168	Herpes simplex virus 1 infection	2/50	498/8073	0.823249	0.831869	0.603428	7124/5290	2
hsa04060	Cytokine-cytokine receptor interaction	1/50	294/8073	0.844425	0.848823	0.615727	7124	1
hsa04740	Olfactory transduction	1/50	448/8073	0.942937	0.942937	0.683996	801	1

Supplementary File S5

S5.1 Experimental method of pharmacodynamics of PRP for treating arrhythmia zebrafish

The pharmacodynamics experiments were carried out from three aspects: Cardiovascular morphological observation, sinus venosus - bulbus arteriosus (SV-BA) distance measurement and Acridine orange (AO) staining for apoptosis evaluation.

S5.2 Experimental results of pharmacodynamics of PRP for treating arrhythmia zebrafish

The original data of cardiac congestion area of zebrafish in each group and the data analysis results were shown in **Table S11, Table S11-1, Table S11-2.** The original data of SV-BA distance measurement of zebrafish in each group and the data analysis results were shown in **Table S12, Table S12-1, Table S12-2**. The original data of average fluorescence density of zebrafish in each group and the data analysis results were shown in **Table S13, Table S13-1, Table S13-2**.

Table S11 Original data of congestion area of zebrafish in each group

	Control	Model	Positive	PRP-H	PRP-M	PRP-L
1	4547	31067	5357	6086	7340	14687
2	4921	32411	5904	6900	7905	15902
3	3408	30998	5187	6599	7470	14600
4	5175	29975	5824	7085	8024	19562
5	3721	27546	5431	7132	8321	16771
6	4025	29980	6078	6134	7856	13952
Means	4299.5	30329.5	5630.167	6656	7819.333	15912.333
SD	695.529	1632.123	353.212	462.771	361.673	2055.434

Means ± SD	4299.5 ±695.529	30329.5 ±1632.123	5630.167 ±353.212	6656 ±462.771	7819.333 ±361.673	15912.333 ±2055.434

Table S11-1 Homogeneity of variance results

expression quantity	Levene statistical magnitude	df1	df2	significance
	3.457	5	30	0.014<0.05

Table S11-2 Multiple comparison results

	(I) Grouping	(J) Grouping	The mean difference (I-J)	standard error	significance
Tamhane	1	2	-26030.000*	724.291	0
		3	-1330.667	318.465	0.053
		4	-2356.500*	341.057	0.001
		5	-3519.833*	320.044	0
		6	-11612.833*	885.868	0
	2	1	26030.000*	724.291	0
		3	24699.333*	681.736	0
		4	23673.500*	692.578	0
		5	22510.167*	682.475	0
		6	14417.167*	1071.497	0
	3	1	1330.667	318.465	0.053
		2	-24699.333*	681.736	0
		4	-1025.833*	237.668	0.026
		5	-2189.167*	206.384	0
		6	-10282.167*	851.427	0.001
	4	1	2356.500*	341.057	0.001
		2	-23673.500*	692.578	0
		3	1025.833*	237.668	0.026
		5	-1163.333*	239.779	0.012
		6	-9256.333*	860.132	0.001
	5	1	3519.833*	320.044	0
		2	-22510.167*	682.475	0
		3	2189.167*	206.384	0
		4	1163.333*	239.779	0.012
		6	-8093.000*	852.019	0.002
	6	1	11612.833*	885.868	0
		2	-14417.167*	1071.497	0
		3	10282.167*	851.427	0.001
		4	9256.333*	860.132	0.001
		5	8093.000*	852.019	0.002

Table S12 Original data of SV-BA interval of zebrafish in each group

	Control	Model	Positive	PRP-H	PRP-M	PRP-L
1	139.98	218.26	149.74	156.47	167.26	187.23

2	141.25	219.89	152.37	160.32	160.57	184.76
3	138.27	214.32	150.86	163.45	161.59	190.14
4	140.25	220.57	158.4	157.81	170.68	187.35
5	143.22	226.73	154.31	164.94	169.96	179.4
6	140.77	234.2	150.72	159.74	158.2	180.69
Means	140.623	222.328	152.733	160.455	164.71	184.928
SD	1.628	7.072	3.202	3.24	5.272	4.168
Means ± SD	140.623 ±1.628	222.328 ±7.072	152.733 ±3.202	160.455 ±3.24	164.71 ±5.272	184.928 ±4.168

Table S12-1 Homogeneity of variance results

Expression quantity	Levene statistical magnitude	df1	df2	significance
	3.074	5	30	0.023<0.05

Table S12-2 Multiple comparison results

	(I) Grouping	(J) Grouping	The mean difference (I-J)	standard error	significance
Tamhane	1	2	-81.705*	2.962	0
		3	-12.110*	1.466	0.001
		4	-19.832*	1.48	0
		5	-24.087*	2.252	0.001
		6	-44.305*	1.827	0
	2	1	81.705*	2.962	0
		3	69.595*	3.169	0
		4	61.873*	3.176	0
		5	57.618*	3.601	0
		6	37.400*	3.351	0
	3	1	12.110*	1.466	0.001
		2	-69.595*	3.169	0
		4	-7.722*	1.86	0.029
		5	-11.977*	2.518	0.02
		6	-32.195*	2.146	0
	4	1	19.832*	1.48	0
		2	-61.873*	3.176	0
		3	7.722*	1.86	0.029
		5	-4.255	2.526	0.874
		6	-24.473*	2.155	0
	5	1	24.087*	2.252	0.001
		2	-57.618*	3.601	0
		3	11.977*	2.518	0.02
		4	4.255	2.526	0.874
		6	-20.218*	2.744	0
	6	1	44.305*	1.827	0
		2	-37.400*	3.351	0

	3	32.195*	2.146	0
	4	24.473*	2.155	0
	5	20.218*	2.744	0

Table S13 Original data of average fluorescence density of zebrafish in each group

	Control	Model	Positive	PRP-H	PRP-M	PRP-L
1	0.119	0.178	0.123	0.134	0.139	0.154
2	0.121	0.192	0.125	0.137	0.14	0.157
3	0.115	0.173	0.121	0.1311	0.143	0.151
4	0.132	0.182	0.12	0.142	0.145	0.158
5	0.126	0.179	0.126	0.13	0.14	0.15
6	0.108	0.186	0.125	0.142	0.141	0.162
Means	0.12	0.182	0.123	0.136	0.141	0.155
SD	0.008	0.007	0.002	0.005	0.002	0.005
Means ± SD	0.12 ±0.008	0.182 ±0.007	0.123 ±0.002	0.136 ±0.005	0.141 ±0.002	0.155 ±0.005

Table S13-1 Homogeneity of variance results

Expression quantity	Levene statistical magnitude	df1	df2	significance
	2.080	5	30	0.096>0.05

Table S13-2 Multiple comparison results

	(I) Grouping	(J) Grouping	The mean difference (I-J)	standard error	significance
LSD	1	2	-.061*	0.003	0
		3	-0.003	0.003	0.316
		4	-.016*	0.003	0
		5	-.021*	0.003	0
		6	-.035*	0.003	0
	2	1	.061*	0.003	0
		3	.058*	0.003	0
		4	.046*	0.003	0
		5	.040*	0.003	0
		6	.026*	0.003	0
	3	1	0.003	0.003	0.316
		2	-.058*	0.003	0
		4	-.013*	0.003	0
		5	-.018*	0.003	0
		6	-.032*	0.003	0
	4	1	.016*	0.003	0
		2	-.046*	0.003	0
		3	.013*	0.003	0
		5	-0.005	0.003	0.097
		6	-.019*	0.003	0

5	1	.021*	0.003	0
	2	-.040*	0.003	0
	3	.018*	0.003	0
	4	0.005	0.003	0.097
	6	-.014*	0.003	0
6	1	.035*	0.003	0
	2	-.026*	0.003	0
	3	.032*	0.003	0
	4	.019*	0.003	0
	5	.014*	0.003	0

Supplementary File S6

S6.1 Quantitative Reverse Transcriptase Polymerase Chain Reaction Analysis (qRT-PCR analysis) and Quantitative determination of adenosine and cGMP metabolites

After treatment at 96 hpf, zebrafish embryos were randomly selected (n=30 each group) for qRT-PCR analysis. Thirty milligrams of zebrafish embryos were homogenized in TRIzol reagentto extract total RNA. The extracted RNA was used as a template, and cDNA was synthesized by reverse transcription with appropriate primers according to the instructions of the Servicebio®RT First Strand cDNA Synthesis kit. qRT-PCR was performed in an ABI 7900HT FastReal-Time PCR system. The primer sequences were as follows: TTCTGACCCAAAGTTCCATCCT (forward) and CTCAAACTGGCAGGTGACGAT (reverse). Levels of the ADORA1 mRNAs were normalized to the GAPDH mRNA and determined using the $2^{-\Delta\Delta Ct}$ method, and all reactions were performed in triplicate.

adenosine and cGMP metabolite markers were quantitated by comparing to the areas of the peaks for the external standards.

S6.2 PRP rescues metabolite profiles from the ADORA1-mediated cGMP-PKG signalling pathway

By quantifying the levels of adenosine and cGMP in embryo samples using LC-MS in multiple reaction monitoring (MRM) mode, the results were shown in **Table S14, Table S14-1, Table S14-2 and Table S15, Table S15-1, Table S15-2**. ADORA1 expression was determined using qRT-PCR assays to examine how the PRP treatment influenced barium chloride induced arrhythmia in zebrafish embryos, the results were shown in **Table S16,**

Table S16-1, Table S16-2.

Table S14 Original data of adenosine of in each group

	Control	Model	Positive	PRP-H	PRP-M	PRP-L
1	3686	1154	2616	2236	2116	1832
2	3474	1236	2384	1934	2127	1833
3	2482	1168	2498	1973	1504	1487
4	3368	1012	2332	2097	1983	1558
5	3293	1233	2483	2191	2075	1498
6	3097	1317	2117	2089	2065	1882
Means	3233.333	1186.667	2405	2086.667	1978.333	1681.667
SD	416.56	103.432	172.028	117.92	237.875	185.773
Means ± SD	3233.333± 416.56	1186.667± 103.432	2405± 172.028	2086.667± 117.92	1978.333± 237.875	1681.667± 185.773

Table S14-1 Homogeneity of variance results

Expression quantity	Levene statistical magnitude	df1	df2	significance
	1.979	5	30	0.111>0.05

Table S14-2 Multiple comparison results

	(I) Grouping	(J) Grouping	The mean difference (I-J)	standard error	significance
LSD	1	2	2046.667*	133.086	0
		3	828.333*	133.086	0
		4	1146.667*	133.086	0
		5	1255.000*	133.086	0
		6	1551.667*	133.086	0
	2	1	-2046.667*	133.086	0
		3	-1218.333*	133.086	0
		4	-900.000*	133.086	0
		5	-791.667*	133.086	0
		6	-495.000*	133.086	0.001
	3	1	-828.333*	133.086	0
		2	1218.333*	133.086	0
		4	318.333*	133.086	0.023
		5	426.667*	133.086	0.003
		6	723.333*	133.086	0
	4	1	-1146.667*	133.086	0
		2	900.000*	133.086	0
		3	-318.333*	133.086	0.023
		5	108.333	133.086	0.422
		6	405.000*	133.086	0.005
	5	1	-1255.000*	133.086	0
		2	791.667*	133.086	0

		3	-426.667*	133.086	0.003
		4	-108.333	133.086	0.422
		6	296.667*	133.086	0.033
	6	1	-1551.667*	133.086	0
		2	495.000*	133.086	0.001
		3	-723.333*	133.086	0
		4	-405.000*	133.086	0.005
		5	-296.667*	133.086	0.033

Table S15 Original data of cGMP of in each group

	Control	Model	Positive	PRP-H	PRP-M	PRP-L
1	59280	11820	46560	39330	27340	24350
2	84000	11050	50220	37710	30680	21630
3	87420	11280	50840	38870	29500	20480
4	91930	12310	51080	39520	30460	19670
5	94870	12490	50250	40390	31420	19920
6	97500	11550	51050	37780	33600	20050
Means	85833.33	11750	50000	38933.33	30500	21016.67
SD	13898.92	568.683	1727.368	1044.426	2072.486	1773.467
Means ± SD	85833.333±13898.92	11750±568.683	50000±1727.368	38933.333±1044.426	30500±2072.486	21016.667±1773.467

Table S15-1 Homogeneity of variance results

Expression quantity	Levene statistical magnitude	df1	df2	significance
	4.765	5	30	0.003<0.05

Table S15-2 Multiple comparison results

	(I) Grouping	(J) Grouping	The mean difference (I-J)	standard error	significance
Tamhane	1	2	74083.333*	5678.958	0.001
		3	35833.333*	5717.864	0.02
		4	46900.000*	5690.208	0.006
		5	55333.333*	5736.944	0.002
		6	64816.667*	5720.215	0.001
	2	1	-74083.333*	5678.958	0.001
		3	-38250.000*	742.428	0
		4	-27183.333*	485.494	0
		5	-18750.000*	877.363	0
		6	-9266.667*	760.327	0
	3	1	-35833.333*	5717.864	0.02
		2	38250.000*	742.428	0
		4	11066.667*	824.078	0

	5	19500.000*	1101.438	0
	6	28983.333*	1010.692	0
4	1	-46900.000*	5690.208	0.006
	2	27183.333*	485.494	0
	3	-11066.667*	824.078	0
	5	8433.333*	947.455	0
	6	17916.667*	840.239	0
5	1	-55333.333*	5736.944	0.002
	2	18750.000*	877.363	0
	3	-19500.000*	1101.438	0
	4	-8433.333*	947.455	0
	6	9483.333*	1113.582	0
6	1	-64816.667*	5720.215	0.001
	2	9266.667*	760.327	0
	3	-28983.333*	1010.692	0
	4	-17916.667*	840.239	0
	5	-9483.333*	1113.582	0

Table S16 Original data of qRT-PCR of zebrafish in each group

	Control	Model	Positive	PRP-H	PRP-M	PRP-L
1	2.15	0.558	1.394	1.169	1.269	0.886
2	1.808	0.323	1.513	1.235	1.176	0.891
3	1.985	0.21	1.68	1.544	0.659	0.999
Means	1.981	0.364	1.529	1.316	1.035	0.925
SD	0.171	0.178	0.144	0.2	0.329	0.064
Means ± SD	1.981 ±0.171	0.364± 0.178	1.529 ±0.144	1.316 ±0.200	1.035 ±0.329	0.925 ±0.064

Table S16-1 Homogeneity of variance results

Expression quantity	Levene statistical magnitude	df1	df2	significance
	2.008	5	30	0.150>0.05

Table S16-2 Multiple comparison results

	(I) Grouping	(J) Grouping	The mean difference (I-J)	standard error	significance
LSD	1	2	1.620*	0.161	0
		3	.457*	0.161	0.015
		4	.667*	0.161	0.001
		5	.947*	0.161	0
		6	1.057*	0.161	0
	2	1	-1.620*	0.161	0
		3	-1.163*	0.161	0
		4	-.953*	0.161	0
		5	-.673*	0.161	0.001
		6	-.563*	0.161	0.004

3	1	-.457*	0.161	0.015
	2	1.163*	0.161	0
	4	0.21	0.161	0.217
	5	.490*	0.161	0.01
	6	.600*	0.161	0.003
4	1	-.667*	0.161	0.001
	2	.953*	0.161	0
	3	-0.21	0.161	0.217
	5	0.28	0.161	0.108
	6	.390*	0.161	0.032
5	1	-.947*	0.161	0
	2	.673*	0.161	0.001
	3	-.490*	0.161	0.01
	4	-0.28	0.161	0.108
	6	0.11	0.161	0.507
6	1	-1.057*	0.161	0
	2	.563*	0.161	0.004
	3	-.600*	0.161	0.003
	4	-.390*	0.161	0.032
	5	-0.11	0.161	0.507
